# A Broadly Neutralizing Antibody Targets the Dynamic HIV Envelope Trimer Apex via a Long, Rigidified, and Anionic β-Hairpin Structure

**DOI:** 10.1016/j.immuni.2017.03.017

**Published:** 2017-04-18

**Authors:** Jeong Hyun Lee, Raiees Andrabi, Ching-Yao Su, Anila Yasmeen, Jean-Philippe Julien, Leopold Kong, Nicholas C. Wu, Ryan McBride, Devin Sok, Matthias Pauthner, Christopher A. Cottrell, Travis Nieusma, Claudia Blattner, James C. Paulson, Per Johan Klasse, Ian A. Wilson, Dennis R. Burton, Andrew B. Ward

**Affiliations:** 1 Department of Integrative Structural and Computational Biology, The Scripps Research Institute, La Jolla, CA 92037, USA; 2 Center for HIV/AIDS Vaccine Immunology and Immunogen Discovery, International AIDS Vaccine Initiative Neutralizing Antibody Center and Collaboration for AIDS Vaccine Discovery, The Scripps Research Institute, La Jolla, CA 92037, USA; 3 Department of Immunology and Microbiology, The Scripps Research Institute, La Jolla, CA 92037, USA; 4 Weill Medical College of Cornell University, New York, New York 10065, USA; 5 Program in Molecular Structure and Function, Hospital for Sick Children Research Institute, and Departments of Biochemistry and Immunology, University of Toronto, Toronto, Ontario M5S 1A8, Canada; 6 Department of Cell and Molecular Biology and Chemical Physiology, The Scripps Research Institute, La Jolla, California 92037, USA; 7 Skaggs Institute for Chemical Biology, The Scripps Research Institute, La Jolla, CA 92037, USA; 8 Ragon Institute of MGH, MIT and Harvard, Boston, MA 02139, USA

**Keywords:** HIV, envelope glycoprotein, PGT145, broadly neutralizing antibody, trimer apex, cryo-electron microscopy

## Abstract

Broadly neutralizing antibodies (bnAbs) to HIV delineate vaccine targets and are prophylactic and therapeutic agents. Some of the most potent bnAbs target a quaternary epitope at the apex of the surface HIV envelope (Env) trimer. Using cryo-electron microscopy, we solved the atomic structure of an apex bnAb, PGT145, in complex with Env. We showed that the long anionic HCDR3 of PGT145 penetrated between glycans at the trimer 3-fold axis, to contact peptide residues from all three Env protomers, and thus explains its highly trimer-specific nature. Somatic hypermutation in the other CDRs of PGT145 were crucially involved in stabilizing the structure of the HCDR3, similar to bovine antibodies, to aid in recognition of a cluster of conserved basic residues hypothesized to facilitate trimer disassembly during viral entry. Overall, the findings exemplify the creative solutions that the human immune system can evolve to recognize a conserved motif buried under a canopy of glycans.

## Highlights

• Apex binding antibody PGT145 engages all three gp120 protomers simultaneously
• Epitope recognition is chemical-feature specific
• PGT145-class antibodies exhibit structural features that reflect bovine antibodies
• PGT145-class antibody maturation is dependent on structural stabilization of HCDR3

## Teaser

Broadly neutralizing antibodies of the PGT145-family target the HIV-1 Env trimer apex via a long β-hairpin HCDR3, but the molecular basis of recognition is unknown. Using cryoEM, Lee et al. (2017) reveal how PGT145 binds its quaternary epitope and the importance of HCDR2 evolution despite its lack of contacts with Env.

## Introduction

Numerous antibodies that target and neutralize a broad range of different human immunodeficiency virus (HIV) isolates have been found in chronically infected HIV donors. Some of these bnAbs inhibit HIV Env with remarkable breadth and potency by recognizing conserved “supersites” of vulnerability (Burton and Hangartner, 2016). One of these epitope clusters is located at the trimer apex, consisting of the variable loops 1 and 2 (V1/V2) that hold together the gp120 subunits of the trimer through inter-protomer interactions (Doria-Rose et al., 2014, McLellan et al., 2011, Sok et al., 2014, Walker et al., 2009).

True to its name, the V1/V2 region varies greatly in sequence and length. All HIV isolates nevertheless retain two notable features in this region. The V2 contains N-linked glycosylation sites at positions N160 and N156 (or the less common compensatory position N173), and a cluster of positively charged amino acids around the trimer 3-fold symmetry axis (Andrabi et al., 2015). In this manner, the trimer apex forms an immunogenic, structurally conserved motif consisting of an electropositive hole surrounded by N-linked glycans. Examples of patient-derived bnAbs that belong to this class include PG9, PG16, CH01-CH04, the CAP256-VRC26 lineage, PGT141-145, and PGDM1400-1412 (Doria-Rose et al., 2014, McLellan et al., 2011, Sok et al., 2014, Walker et al., 2011, Walker et al., 2009). PGDM1400 (83% breadth, 0.003 μg/mL median IC_50_) and CAP256-VRC26.25 (57% breadth, 0.001 μg/mL median IC_50_), in particular, are remarkably potent (Doria-Rose et al., 2015, Sok et al., 2014).

Partial descriptions of paratope-epitope interactions have been obtained using epitope scaffolds with PG9 (McLellan et al., 2011), PG16 (Pancera et al., 2013), and the CH01-CH04 apex bnAbs (Gorman et al., 2016). Hybrid-modeling approaches employing low-resolution negative-stain EM (Julien et al., 2013b) and X-ray structures of scaffolds indicate these bnAbs bind at or near the trimer 3-fold axis with a binding stoichiometry of one antigen-binding fragment (Fab) per trimer. This binding mode results in a symmetry mismatch, unique to this class of antibodies, and glycan heterogeneity makes them difficult targets for structural studies (Sok et al., 2014). All characterized apex bnAbs, except for some CAP256-VRC26 lineage antibodies (Doria-Rose et al., 2014), depend on glycans at N160 and N156/N173, and often fail to bind viruses produced in the presence of α-mannosidase-I inhibitor kifunensine (Kif) that results in homogeneous oligomannose glycans with 8-9 mannose (Man) residues (Andrabi et al., 2015, Sok et al., 2014). The structural basis of Env recognition for the PGT145-class of antibodies is highly sought after because its quaternary specificity is now widely exploited to detect and isolate properly formed Env trimers (de Taeye et al., 2015, Pugach et al., 2015), including under GMP conditions for human vaccine trials. Using cryo-electron microscopy (cryoEM), we determined the structure of PGT145 Fab in complex with the soluble, recombinant Env trimer, BG505 SOSIP.664 (Sanders et al., 2013) to elucidate key molecular interactions at the Env apex. Our structural and biochemical analyses revealed that PGT145-class bnAbs utilize their CDR loops, especially HCDR2 to stabilize a long anti-parallel β-hairpin HCDR3. This structural rigidity allows the antibody to penetrate through the tightly packed N160 glycan shield network, to recognize the electropositive sink generated by the protein elements at the core of the trimer apex. Therefore, despite nearly all epitope contacts coming from the HCDR3, additional maturation of the remaining CDR loops influences the HCDR3 and is crucial for generating a potent PGT145-like antibody.

## Results

### PGT145 Recognizes a Quaternary Epitope at the Apical 3-fold Symmetry Axis of the Env Trimer

**Figure 1:**
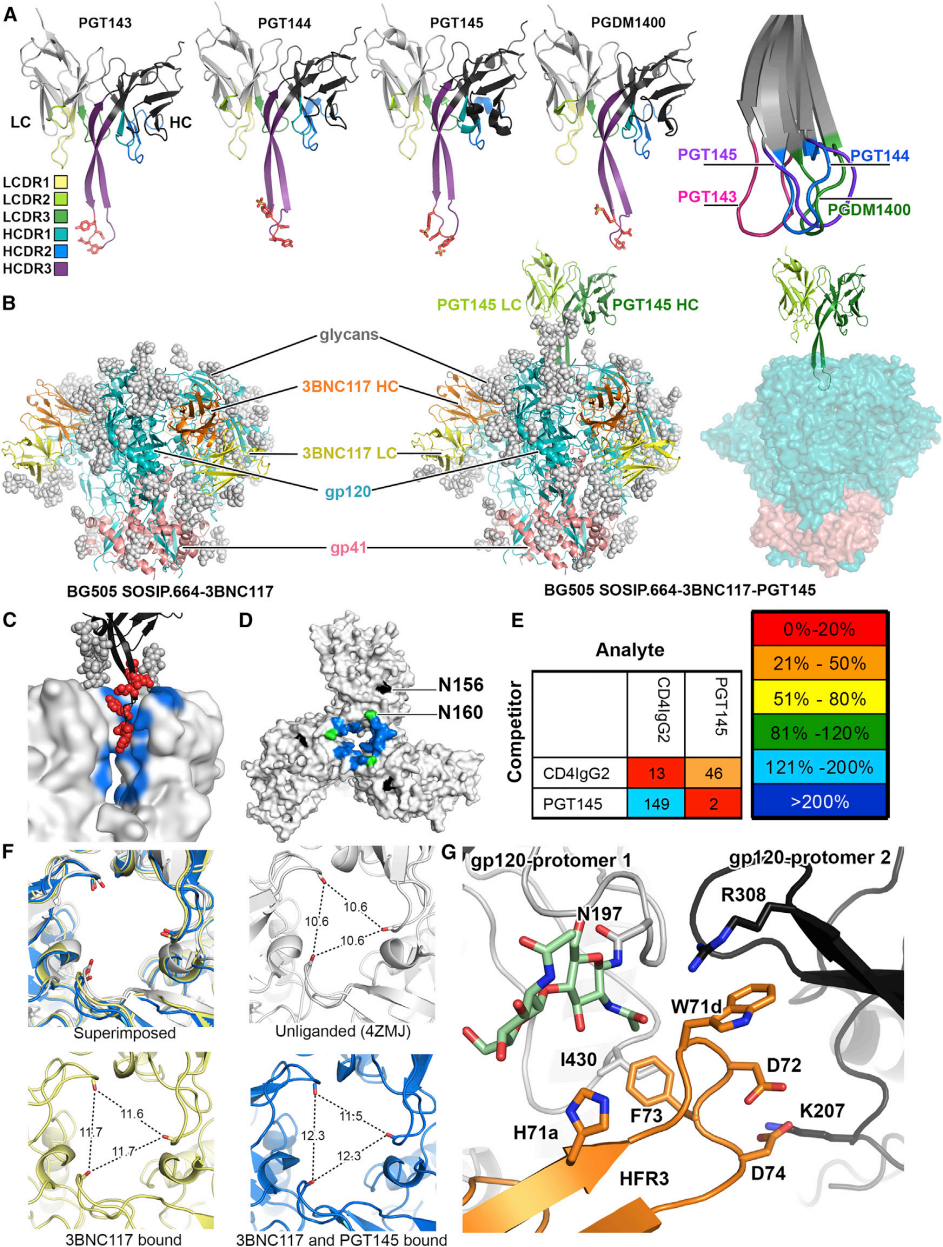
BG505 SOSIP Trimer in Complex with PGT145 Reveals HCDR3 Guided Epitope Recognition at the Center of the Trimer Apex (A) X-ray structures of PGT145-family Fabs. Red sticks: acidic residues and observed or predicted sulfated tyrosines in the β-turn. The CDRs are colored according to the key shown on the far left. HC and LC are shown in dark and light gray respectively. Far right: superimposition of all HCDR3s shows different β-loop conformations. (B) Models of 3BNC117 (left) and 3BNC117+PGT145 (middle) bound trimers. Glycans are shown as white spheres. PGT145 binds at a distance from the trimer as shown in the trimer model with glycans removed (right). (C) PGT145 HCDR3 (black) inserts into a pit created by N160 glycans (gray spheres) and gp120 (gray surface), where its electronegative side chains (red) contact the electropositive epitope (blue). (D) Env residues within a 4 Å radius of the PGT145 CDR loops (blue). (E) SPR competition assay illustrating the non-reciprocal relationship between CD4 and PGT145 binding. PGT145 enhances CD4 binding while CD4 induces a conformational change that destroys the PGT145 epitope. (F) 3-fold apex regions of 3BNC117-bound (yellow), 3BNC117^+^PGT145-bound (blue) and unliganded (gray) trimer. Inter-V2 distances (Å) are measured between the Cα residues of R166 (dashed lines). (G) 3BNC117 HFR3 interaction with the proximal (gray) and adjacent (black) gp120 protomers.

Apex bnAbs discovered so far can be grouped according to their heavy chain (HC) complementarity determining region (CDR) 3 topology: (1) PG9-like with—or predicted to have—a hammerhead motif (Doria-Rose et al., 2015, Doria-Rose et al., 2014, Gorman et al., 2016, McLellan et al., 2011, Pancera et al., 2013); or (2) PGT145-like with a long, anti-parallel β-hairpin (McLellan et al., 2011, Sok et al., 2014). Here, we solved X-ray structures of unliganded PGT143 and PGT144 Fabs, and they too exhibit the β-hairpin HCDR3 motif as expected (Figure 1A, Table 1, Figure S1). The elongated HCDR3 conformation of this PGT145-class bnAbs results in a paratope that projects a long distance away from the surface of the Fab and enables epitope recognition at the C3 axis of the trimer apex via a long-range interaction (Figures 1B and 1C, S2A) (Sok et al., 2014). To define the molecular interactions of an apex antibody, we generated the structures of BG505 SOSIP.664-3BNC117-PGT145 and BG505 SOSIP.664-3BNC117 by single particle cryoEM at global resolutions of ∼4.3 Å and ∼4.4 Å, respectively (Figures 1B, S2B–S2G). The PGT145 HCDR3 inserted perpendicularly to the V1/V2 β sheets between the triad of N160 glycans into a pocket formed at the trimer apex, to simultaneously engage all three gp120s (Figures 1B–1D). This quaternary epitope is only present in the correctly folded pre-fusion Env conformation (Sok et al., 2014).

The trimer-specific PGT145 is one of the few antibodies with which we could previously extract cleaved wild-type JR-FL E168K EnvΔCT trimers from cell membranes (Figure S2A) (Blattner et al., 2014). PGT145 also increases the thermal stability of BG505 SOSIP.664 upon binding (Cheng et al., 2015) and is predicted to neutralize HIV by stabilizing the prefusion conformation of the Env trimer. However, surface plasmon resonance (SPR) showed improved binding of the Env-receptor mimic CD4-IgG2 (Liu et al., 2008), when PGT145 Fab had pre-bound the trimer (Figures 1E, S3A). A subtle opening of the trimer apex induced by PGT145 HCDR3 (Figure 1F) might improve access to the CD4-binding site, albeit shown only in vitro with soluble components. In vivo, PGT145 IgG binding at the Env apex would however provide a steric block to CD4 binding on the host cell membrane.

In the BG505-3BNC117 complex, we also observed a subtle opening of the trimer apex (Kwon et al., 2015) (Figure 1F) that appeared to be mediated by HC framework region 3 (HFR3) of 3BNC117. 3BNC117 and its clonal relative, 3BNC60, have a ^71d^WDFD^74^ insertion in HFR3, which is critical for neutralizing activity (Klein et al., 2013). In 3BNC117, the HFR3 interacts with the N-acetylglucosamine (GlcNAc) core of the N197 glycan with H71a, and residue W71d interacted with V3 R308 of the adjacent gp120 (Figure 1G). Env residue 308 is typically Arg or His (Arg: 32%, His: 39%), both of which provide favorable interactions with tryptophan. Thus, while PGT145 recognizes the closed conformation of the Env trimer, the trimer apex is very subtly open in the PGT145 bound conformation. Induced allosteric effects, such as 3BNC117 binding at the CD4 binding site, that lead to an increase in spacing between the N160 glycan triad, likely make the apical binding site more accessible to PGT145.

### PGT145 Recognizes Oligomannose Glycans

**Figure 2:**
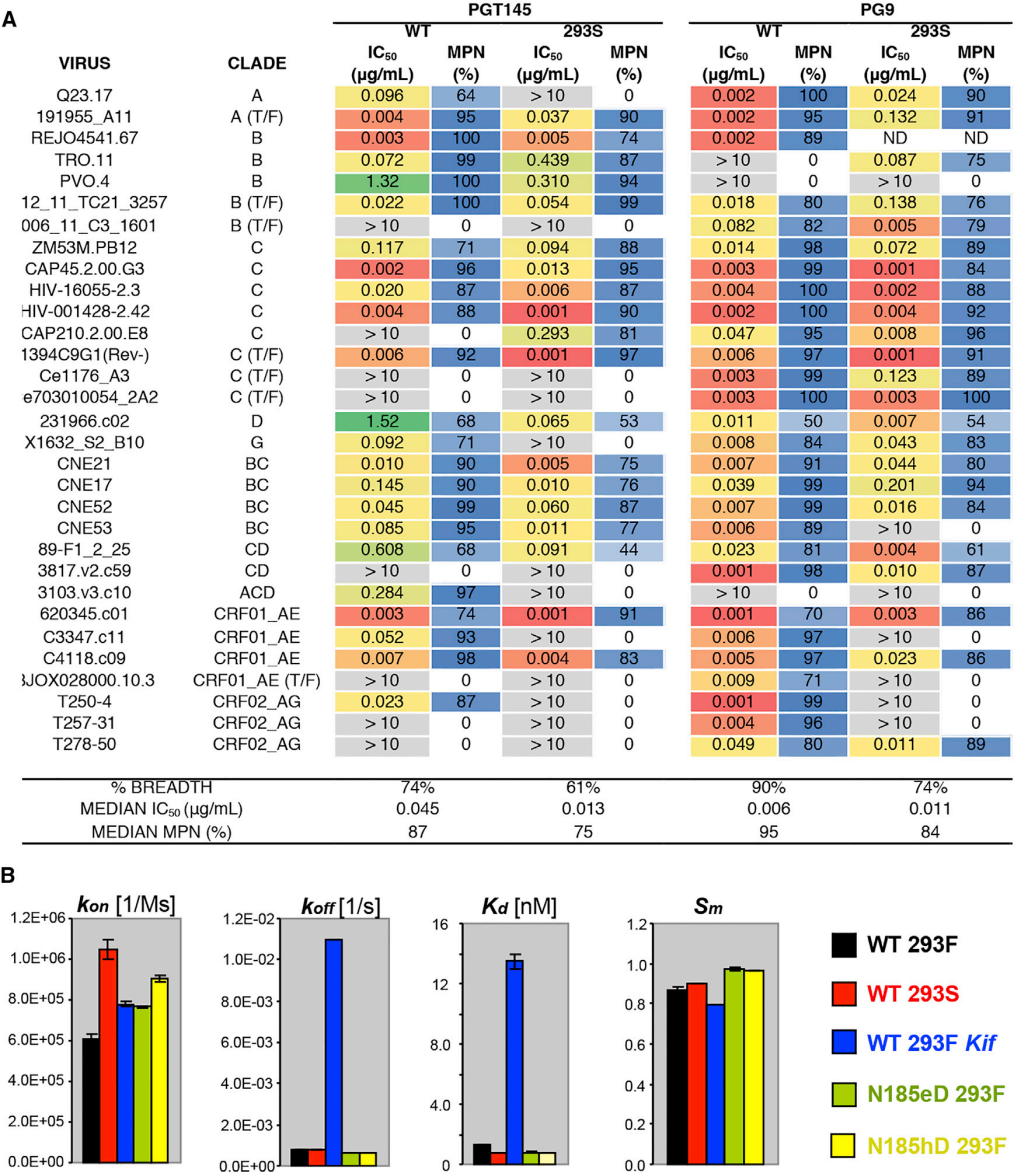
PGT145 Favorably Binds Oligomannose Glycans (A) IC_50_ and maximum percentage neutralization (MPN) values for PGT145 and PG9 neutralization of pseudoviruses produced in 293T or 293S cells. ND: Not determined. (B) A comparison of kinetic parameters derived from SPR analyses of PGT145 binding to BG505 SOSIP.664 trimers produced in trimers expressing different glycoforms and N-linked glycosylation site mutations. Also see Figures S3B and S3C.

The glycoforms to which apex antibodies bind have been a matter of debate. Studies have shown that apex antibodies such as PG16 bind sialylated hybrid glycans with much higher affinity than oligomannose glycans (Andrabi et al., 2015, Pancera et al., 2013). Similarly, PG9 showed increased binding affinity, neutralization potency, and maximum percentage neutralization (MPN) in the presence of complex/hybrid glycans (Figure 2A). PGT145-family antibodies have also been shown to preferentially bind to wild-type viruses and trimers compared to Kif treated Envs (Sok et al., 2014). However, SPR analysis of PGT145 Fab binding to BG505 SOSIP.664 trimers demonstrated that PGT145 has the highest affinity for glucose N-acetyltransferase 1 (GnT1)-deficient 293S-produced trimers that only have oligomannose Man_5-9_ glycans, although the larger Man_8-9_ glycans in the +Kif condition are somewhat inhibitory (Figures 2B, S3B and S3C). Further, a 31-pseudovirus neutralization panel showed a ∼3.5-fold improved median IC_50_ of PGT145 in 293S compared to wild-type glycan producing 293T cell produced pseudoviruses (Figure 2A), suggesting that, while PGT145 can accommodate glycan heterogeneity, there is a preference for oligomannose forms.

Previously, glycan arrays failed to detect PGT145 binding across a wide range of glycans (Andrabi et al., 2015). Here, we employed a dendrimer format of glycan array slides (Wang et al., 2008) that creates a higher density of glycans. PGT145 and PGDM1400 bound the C-isomer of a Man_8_ glycan (M8C) (Figures S3D–S3F), but not the Man_8_ B-isomer (M8B) indicating that the terminal Man(α1-2) residue of the D3 arm may sterically clash with PGT145-family Fabs. These data suggested that not only the correct glycoform, but also close spacing of glycans is essential for PGT145 binding.

### The PGT145 Epitope Consists of Glycans from Two Protomers and Peptide Contacts from all Three Protomers

**Figure 3:**
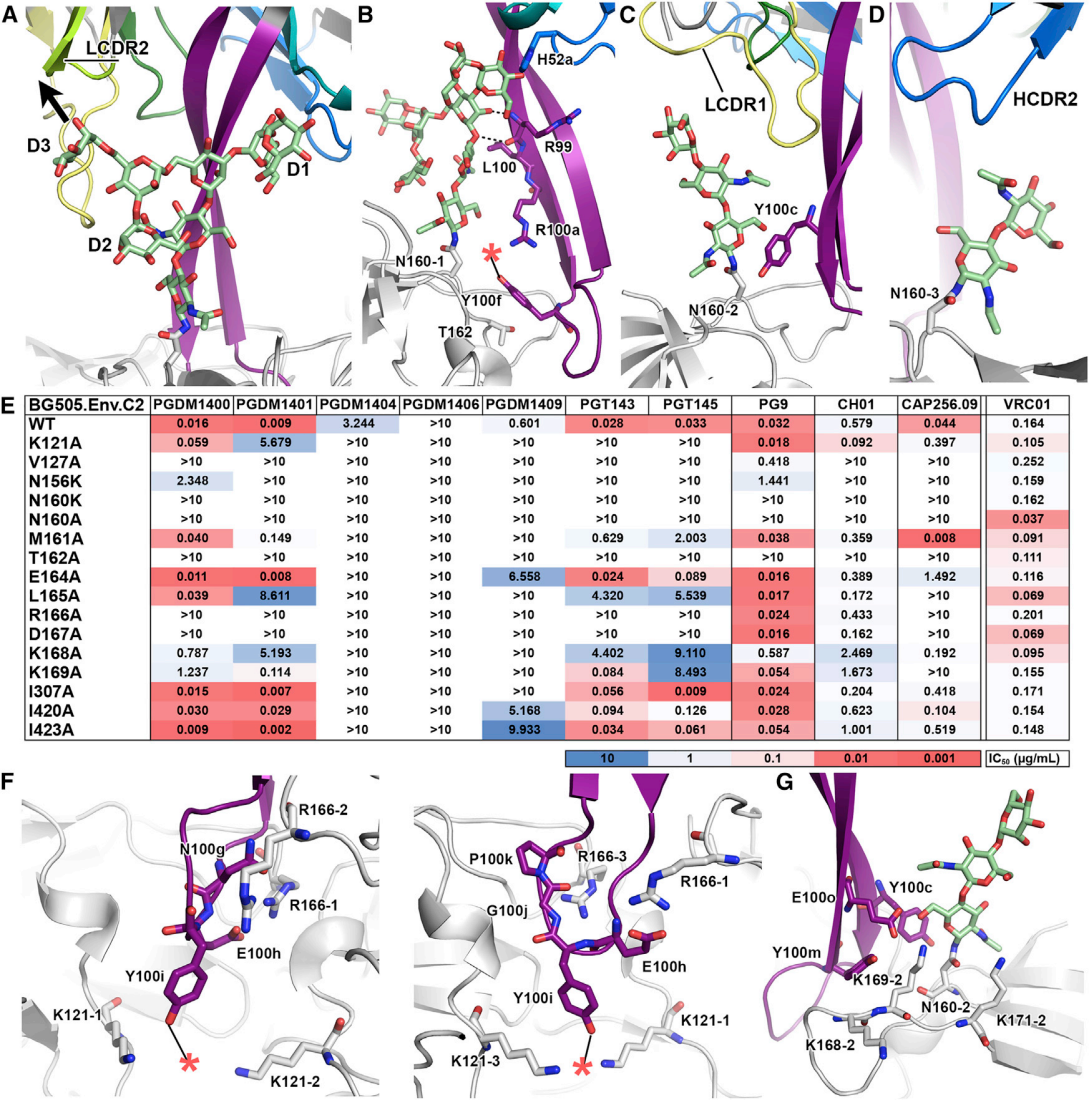
Simultaneous Engagement of Glycans and Protein Residues from Multiple gp120 Protomers Is Required for PGT145-Class bnAb Binding (A) PGT145 interaction with N160_glycan1_. Glycans are shown as green sticks, and the CDRs are colored as in Figure 1A. The arrow indicates the direction of the D3 branch of the glycan. (B) N160_glycan1_ interacts with the PGT145 HCDR3 peptide backbone. The red star indicates a sulfated tyrosine. (C) PGT145 interaction with N160_glycan2._ (D) PGT145 interaction with N160_glycan3_. (E) Neutralization IC_50_ values of 293T cell-produced BG505 pseudovirus mutants by PGT145-family variants. (F) Left and right panels: R166 and K121 from all three gp120 protomers (white) interact with the electronegative moieties in HCDR3. Red star indicates tyrosine sulfation observed in the X-ray structure of the Fab. (G) Residues in HCDR3 along with K169_gp120_ support interactions with the base of N160_glycan2_.

In the cryoEM map, the primary interacting N160 glycan appeared as oligomannose, consistent with our glycan array analysis. None of the asymmetric N160 glycans revealed density corresponding to core fucosylation (Figures S4A and S4B), often found in complex glycans. The total buried surface area between PGT145 and the modeled N160 glycans was ∼555 Å^2^ (glycan 1: 384 Å^2^, glycan 2: 131 Å^2^, glycan 3: 40 Å^2^). PGT145 most prominently interacted with a single N160 glycan (N160_glycan1_), less extensively with a second N160 glycan (N160_glycan2_), and likely not at all with the third N160 glycan (N160_glycan3_) (Figures 3A–3D, S4A–S4C). The density resolved for N160_glycan1_ is approximately the size of Man_6_ (Figure S4A) and its core GlcNAc sugars bound the peptide backbone of the HCDR3 descending strand, while the D1 arm packed between HCDR2 and HCDR3, making polar contacts with highly conserved H52a_HCDR2_ (Figure 3B). Y100f_HCDR3_—a tyrosine known to be sulfated—orients R100a _HCDR3_, and the sulfate might also interact with N160_glycan1_ Asn, while the benzene ring face of Y100f _HCDR3_ is packed against T162 (Figure 2B). Although the D2 and D3 arms of N160_glycan1_ are not fully resolved, we infer from glycan array data that the terminal D3 arm would clash with the LC of PGT145 in its orientation in the structure (Figures 3A and S3E). Together, the data predicted that much of the glycan recognition is driven by interactions made with the GlcNAc stalk, with steric accommodation for a Man_6_, Man_7_, Man_8_, or even a hybrid glycan at N160_glycan1_. In contrast, a complex glycan with its bulkier Gal(β1-4)GlcNAc(β1-2/4/6) branching units would likely clash with PGT145. Thus, the mode of N160 glycan recognition differed from that of the PG9-like antibodies, in which the N160 glycan branches bury into a pocket formed by the hammerhead-shaped HCDR3 and the LCDRs (Figure S4D). The GlcNAc base of N160_glycan2_ interacted with Y100c_HCDR3_ and to some extent with LCDR1, although not well resolved in our structure (Figure 3C). LCDR1 is the most variable CDR segment among the PGT145-family variants (Figure S1B), suggesting that the PGT145-family antibodies might accommodate N160_glycan2_ in different ways. N160_glycan3_ projected away from the antibody likely because it is sterically inhibitory to PGT145 binding in its native conformation (Figures 3D, S4C). Consequently, the N160 glycan is both essential (on one, probably two protomers), as well as inhibitory (on the third protomer) to binding of PGT145, presenting a unique challenge to epitope recognition of an asymmetric antibody molecule at a symmetry axis.

In contrast to hammerhead HCDR3-type apex antibodies, which are dependent on the N156 glycan and bind complex glycoforms on glycan arrays (Andrabi et al., 2015, Gorman et al., 2016, McLellan et al., 2011, Pancera et al., 2013), none of the N156 glycans directly contacted PGT145 (Figures S4D and S4E). Ablation of the N156 glycosylation site by an N156K substitution abrogated neutralization by PGT145 (Figure 3E). We therefore looked for an indirect effect that could be ascribed to differential processing of N160 glycans in N156 glycan-lacking Env variants expressed in different cell-lines. SPR showed negligible binding of PGT145 Fab to all of the N156D substitution variants (Figure S3B), and inspection of the N156D mutant by negative stain EM revealed that this mutation enriched for non-native-like trimers (Figure S4F). Thus, the reduced binding of PGT145 to N156 glycan knockouts most likely arose from disruption of the conformational epitope through the loss of the inter-V2-strand interaction between Y173 and N156 core GlcNAc. Residue 173 is a Tyr, His, or Phe in >86% of Envs, all of which are suitable for stabilizing the N156 glycan. Indirect glycan effects on the PGT145 epitope might also result from variability in the length and number of glycosylation sites on V1 and V2 loops, as such viral attributes have been noted to increase with disease progression and correlate with reduced neutralization sensitivity (Curlin et al., 2010, Sagar et al., 2006). The most variable segment of V2, which is distal to the 3-fold axis, is flexible and disordered in the majority of high-resolution SOSIP.664 structures. The density corresponding to the projection of the V2 loop in BG505, however, suggested that its glycans would be close to the bound PGT145 Fab, and the N-linked glycosylation sites at N185e and N185h could restrict accessibility to the epitope (Figure S4G). Removal of the N185h glycan site in particular, led to a ∼1.5-fold increase in the association constant of PGT145 Fab, and an increase in the stoichiometry (S_m_) from 0.87 to 0.96 (Figures 2B, S3B and S3C), confirming that outer-V2 region glycans can partially shield the apex bnAb epitope.

Peptide contacts between PGT145 and Env (total buried surface area ∼729 Å^2^) are largely restricted to interactions between the tip of HCDR3 with the C-strand of V1/V2 and the C1 region near the base of V1 including K121 (Figure 3F). K121 from each protomer interacted with sulfated tyrosine Y100i, while R166 from each protomer interacted with various electronegative moieties in HCDR3 of PGT145 (Figure 3F). Finally, Y100m_HCDR3_ stacked up against the hydrocarbon segment of K169, which probably helps engage the N160_glycan2_ via a network of interactions along with E100o (Figure 3G).

To confirm our structural observations, we conducted alanine-scanning neutralization assays on BG505 pseudovirus mutants (Figures 3E, S5A). Neutralization was reduced when the N160 or N156 glycan sites were knocked out (N156K, N160K, N160A, T162A), or direct peptide contact residues were substituted (K121A, R166A). Point mutations in V1/V2 that caused BG505 SOSIP.664 trimers to adopt aberrant trimer forms (M161A, L165A, D167A) when observed by EM, selectively decreased neutralization potencies of PGT145-family bnAbs versus hammerhead-group, PG9 and CH01 (Figures 3E, S5). Neutralization by CAP256-VRC26.09 was affected mostly by the same Env mutations that affected the PGT145 class. Thus, despite CAP256-VRC26.09 having a HCDR3 structure that more closely resembles the hammerhead-class (Doria-Rose et al., 2014), it is dependent on proper quaternary apex formation like PGT145, and engages all three protomers upon binding. Overall, our results further classified PGT145 as a distinct class of apex bnAb, compared to PG9, and that it is an N160 glycan-dependent antibody that relies on simultaneous recognition of peptide contacts from the apex central residues of all three gp120 V1V2 loops, and is indirectly affected by the glycan at N156.

### PGT145 Recognizes an Electropositive Sink at the Trimer Apex

Apex-bnAbs recognize the conserved electropositive V2, particularly strand C (Doria-Rose et al., 2012). We looked for additional residues near the central electropositive layer that could add to the overall local charge. K117, which is 97% conserved, is deeply buried under the trimer apex and is likely important for PGT145 recognition. Additionally, 84% of clade B Envs have an arginine or lysine at V3 position 315, instead of glutamine commonly found in other clades, effectively increasing the positive charge potential at the center of the trimer apex (Figure S6). Residue 315 is located between the electropositive layer created by strand C and K121, which is part of the PGT145 epitope, and thus the additional electropositive layer might interact favorably with the electronegative HCDR3 (Figure S6E). However, PGDM1400- and CAP256-VRC26-lineage bnAbs have lower clade B breadth (Doria-Rose et al., 2015, Sok et al., 2014). Therefore, this extra positive residue likely influences trimer apex dynamics through electrostatic repulsion, as often observed in clade B SOSIP trimers (de Taeye et al., 2015, Pugach et al., 2015). An increased tendency to “breathe” and open up at the apex would both alter the apex epitope but also enhance accessibility to the CD4bs, consistent with clade B Env infected-individuals preferentially generating CD4bs bnAb responses, but reduced apex responses (Rusert et al., 2016).

**Figure 4:**
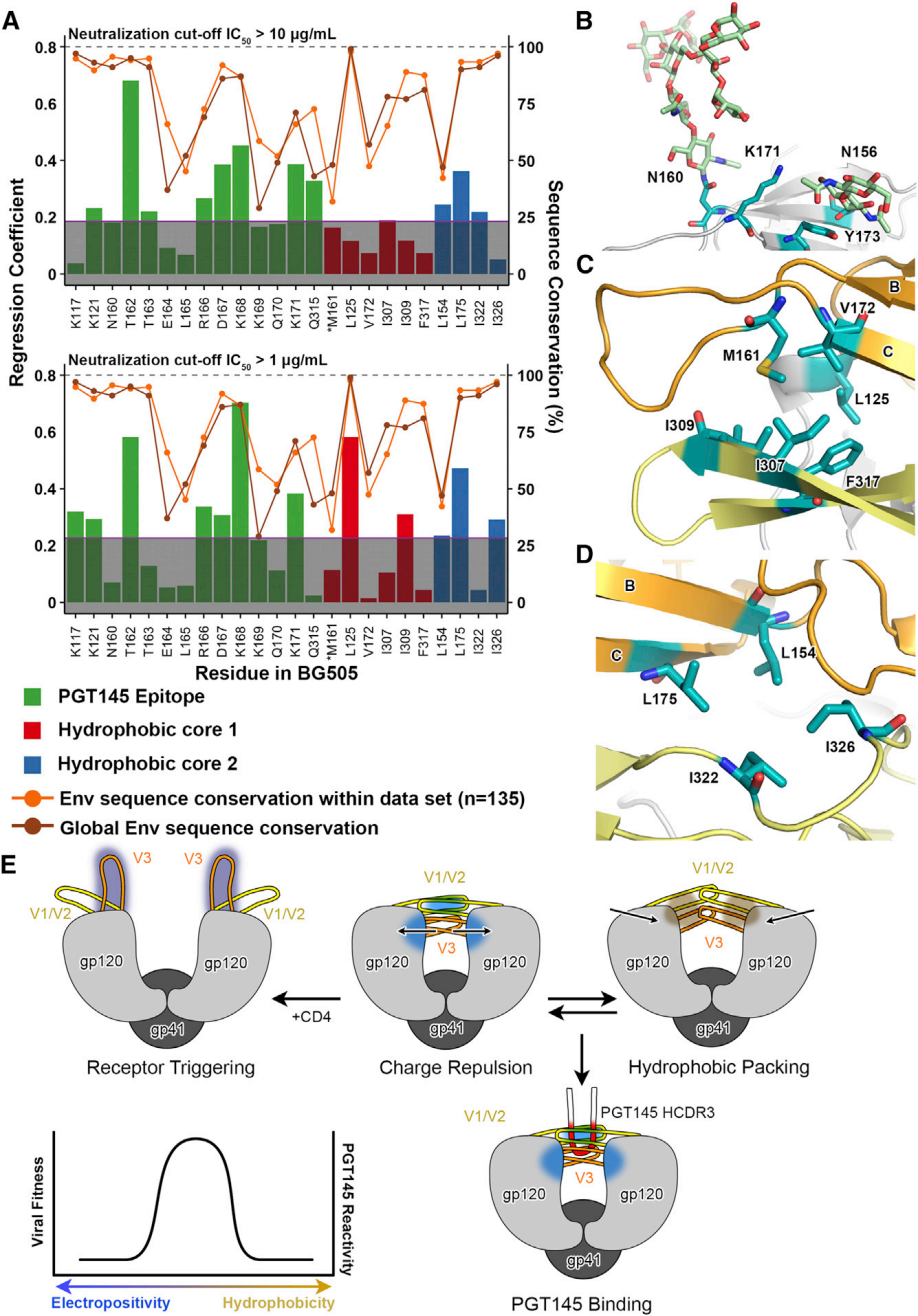
PGT145 IC_50_ Correlates with gp120 Residues Involved in Trimer Stability (A) Logistic regression analysis was used to model neutralization escape based on amino acid sequence information. An IC_50_ of > 10 μg/mL (top) or > 1 μg/mL (bottom) was used to define escape. The relative conservation of the given residue is shown as a line graph. Residue-161 (asterisk) is also part of the direct PGT145 epitope. (B) Y173 and K171 interact with N156 and N160 to stabilize V1/V2 and restrict glycan conformations. (C) Hydrophobic packing interactions in hydrophobic core 1. (D) Stabilizing interactions in hydrophobic core 2. (E) Env is a meta-stable complex that transitions from a closed (top center and right) to an open unliganded trimer (top left) configuration. CD4 triggers a transition to the CD4-bound configuration (left) with an exposed co-receptor binding site (purple patch). The electronegative paratope of PGT145 (red patch, lower right) recognizes an electropositive pocket (blue), which is only present on a stable trimer. Both viral fitness and PGT145 reactivity require balance between electropositivity (blue) and hydrophobicity (tan) at the trimer apex (bottom left graph).

To determine whether there is any correlation between PGT145 IC_50_ and electropositive charges near the PGT145 epitope, we analyzed sequence variability at select positions in relation to neutralization using a linear regression model (Figure 4A). The goal of the model was to weigh the contribution of each residue to the escape phenotype defined by two different IC_50_ cut-off values, by considering the amino acid variation in each strain and their respective neutralization IC_50_ values. This weighting was reflected in the magnitude of the regression coefficient. The model predicts that the larger the magnitude of this coefficient, the more the residue influences resistance. In this analysis, residues that contact PGT145 and are important for neutralization, such as K121, R166, and T162, are also well conserved, having an exact match conservation score (relative to BG505) of ∼70% or higher (Figure 4A). This analysis also revealed that K171 is important even though it does not directly contact PGT145. K171 seemed to interact with the base of the N160 glycan and/or restrict the movement range of N156 and N160 glycans to indirectly affect PGT145 binding (Figures 3G and 4B). A subtler effect on PGT145 neutralization was observed among hydrophobic residues involved in stabilization of the trimer apex (Figures 4A, 4C, and 4D). Particular V1/V2 residues have neutralization regression coefficients that were more prominent with an IC_50_ > 1 μg/mL cut-off, such as L125, I309, L175, and I326, which also tend to be more conserved. In binding, the anionic HCDR3 of PGT145 stabilizes the trimer by offsetting the net charge at the apex, especially when Env contains additional cationic residues. Mechanistically, Env requires CD4 triggering to transition into an open state leading to exposure of the co-receptor binding site. The proximal localization of positively charged residues results in repulsion between the gp120 protomers, contributing to meta-stability and priming Env for CD4 induced conformational changes. Too much repulsion between protomers would result in a constitutively open trimer susceptible to degradation and therefore render the virus defunct. Thus, we propose that a fine balance between tight hydrophobic packing and localization of electropositive charge is required for viral fitness, which PGT145 exploits for epitope targeting (Figure 4E).

### PGT145 Potency Is Reliant on the Stability of Its HCDR3

**Figure 5:**
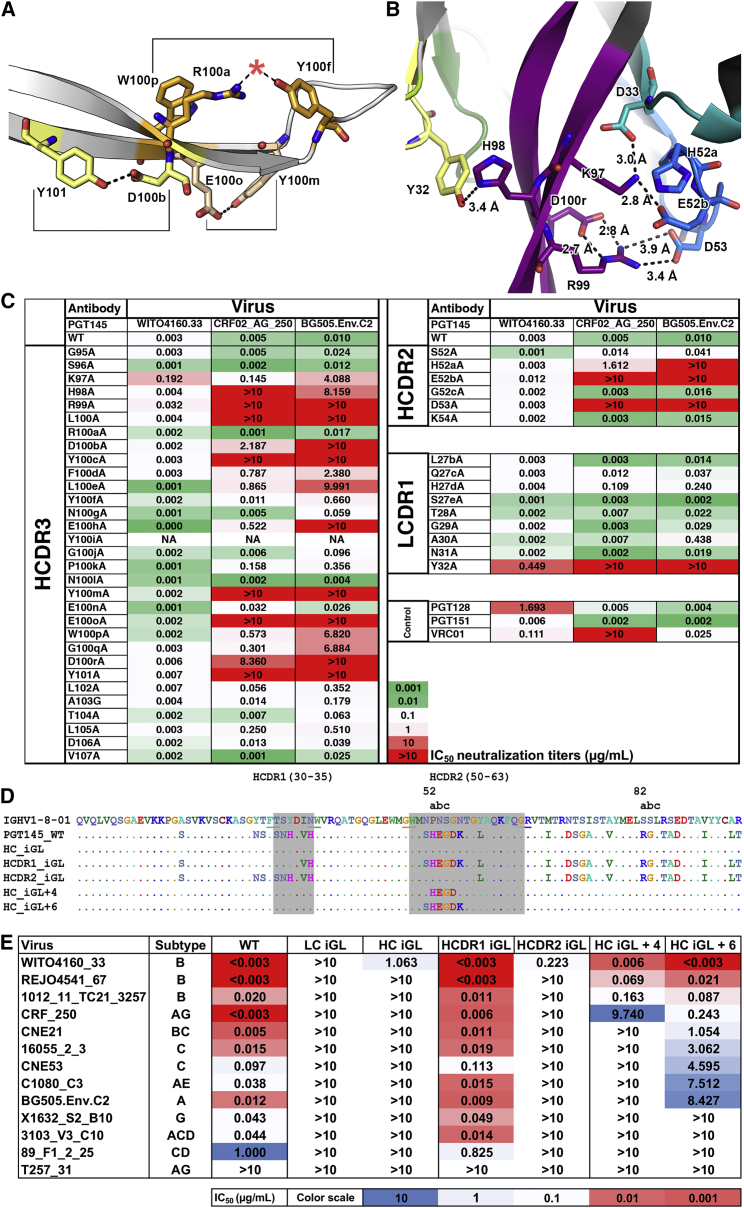
The Neutralization Potency of PGT145 Is Dependent on Stabilization of the HCDR3 (A) Interaction pairs within the HCDR3 are indicated by matching shades of color and brackets. The red star indicates the sulfated tyrosine found in the X-ray structure. (B) The PGT145 HCDR3 forms an electrostatic interaction network with side chains of surrounding CDRs (colored as in Figure 1A). The Fab shown is from the cryoEM model. (C) Neutralization of three different 293T-cell produced pseudoviruses by PGT145 Ala-substitution variants. (D) Definition of iGL variants tested in (E). (E) Neutralization of pseudoviruses by PGT145 iGL variants defined in (D).

**Figure 6:**
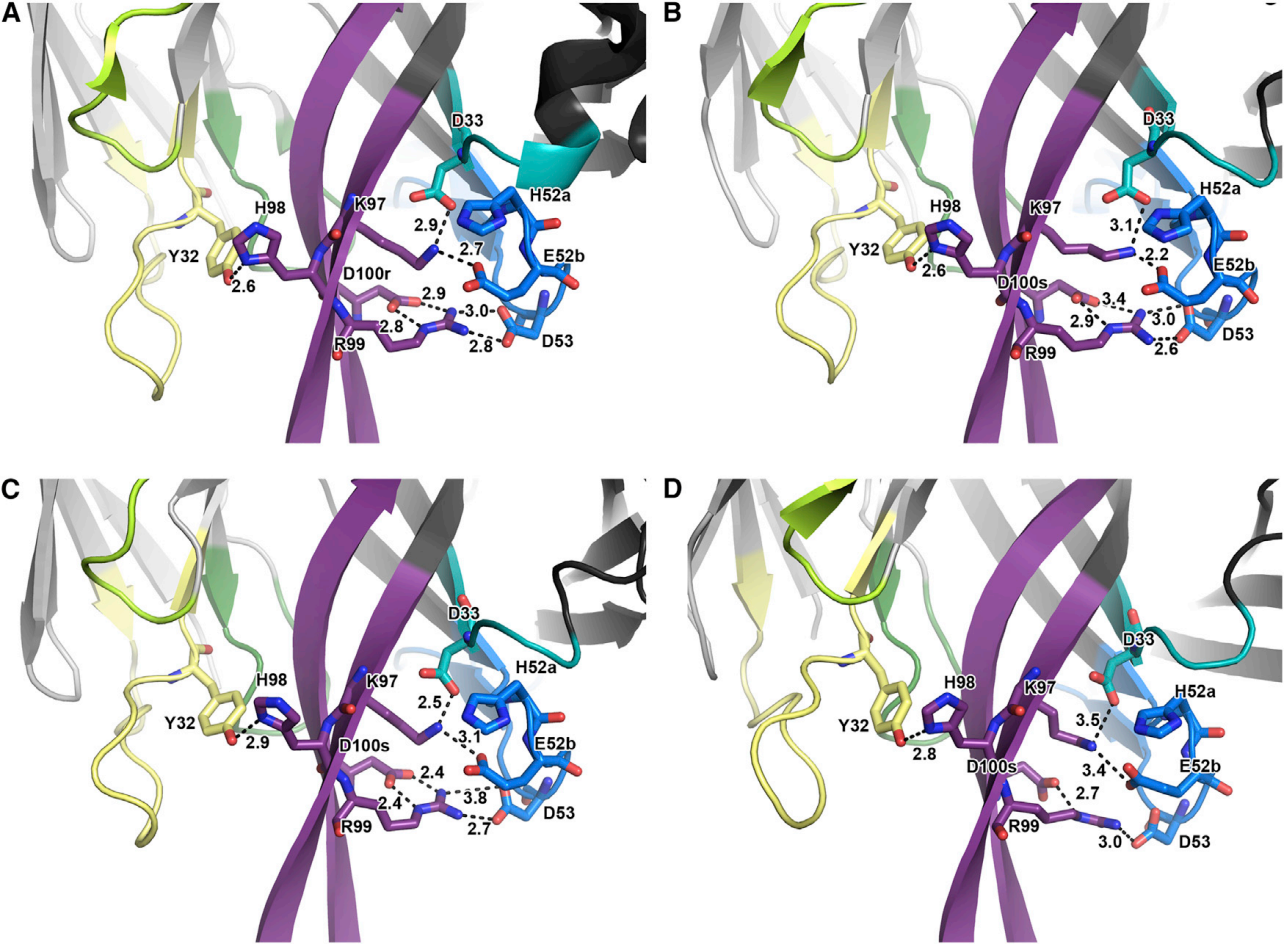
A Network of HCDR3 Stabilizing Interactions Is Found in Multiple PGT145-Class bnAbs Inter-CDR stabilizing interactions (dashed lines, numbers corresponding to measured distance in Å) shown in Figure 5B are also present in the following unliganded X-ray Fab structures: (A) PGT145. (B) PGT144. (C) PGT143. (D) PGDM1400. CDR loops are colored as in Figure 1A.

While HCDR3 comprises the majority of the PGT145 paratope, the other CDR loops play important roles in neutralization. For example, reversion of HCDR2 in PGT143 and PGT145 to their predicted germline sequences abrogates neutralization (Andrabi et al., 2015) despite few direct contacts with Env (Figure 3). Structural examination revealed that HCDR3 makes extensive intra- and inter-CDR contacts in PGT145-family Fabs. Within the HCDR3, Y101 and D100b, and E100o and Y100m form electrostatic pairing interactions. W100p packs against R100a, which interacts with Y100f to orient the tyrosine side chain. Eliminating these pairwise stabilizing interactions reduced PGT145 neutralization (Figures 5A–5C, 6), indicating that structural stabilization of the long HCDR3 via tertiary and quaternary contacts is necessary for neutralizing activity.

We found a ^52a^H-D/E-X-D/Q^53^ motif in HCDR2 that appeared to stabilize the HCDR3 stalk (Figures 5B, 6, S1A). PGT143-PGT145 and PGDM1400 all contained extensive electrostatic interactions between the HCDR2 and 3, such as the interaction between R99_HCDR3_ with D53_HCDR2_ (Q53 in PGDM1403-6) and D100r_HCDR3_. The R99A paratope substitution had the second largest increase in PGT145 IC_50_ against all three pseudoviruses tested (Figure 5C). The D100rA_HCDR3_ or D53A_HCDR2_ substitutions also reduced potency, although not as severely as a single R99A substitution, which simultaneously ablates two electrostatic interactions. K97_HCDR3_ formed electrostatic interactions with E52b_HCDR2_ and D33_HCDR1_, where H52a_HCDR2_, a residue that contacted the N160_glycan1_ also played a role in orienting the side chain of E52b_HCDR2_. K97 is conserved across all PGT145-family mAbs except for PGDM1411, a weak neutralizer that has K98 in place of K97 and R99, thereby incompletely substituting for two basic residues. Further, introduction of the 4- (^52a^H-E-G-D^53^) or 6-residue HCDR2 (^52^S-H-E-G-D-K^54^) stabilization motif back into inferred-germline (iGL; germine reversion with the exception of HCDR3) PGT145 partially rescued the neutralizing activity of PGT145 iGL variants (Figures 5D and 5E). LCDR1 exhibited the greatest variability in CDR length and sequence (Figure S1B). Nevertheless, a semi-conserved Y32_LCDR1_ (9 of 18 MAbs) when mutated to alanine in PGT145 resulted in a large drop in neutralization (Figure 5C). In our four Fab structures, Y32_LCDR1_ hydrogen bonded with the π-nitrogen of conserved H98_HCDR3_ (Figures 5B and 5C). The remaining nine PGDM14XX MAbs had an F32_LCDR1_-F98_HCDR3_ interaction pair instead that plausibly stabilizes the HCDR3 by π-stacking.

Based on the SHM patterns, B cell affinity selection during the Ab maturation process led to four distinct PGT145 Ab sub-lineages (Figures S1A and S1B), reminiscent of a fork, which contains a “shaft” consisting of mutations that accumulated early to give rise to the most recent common ancestor (MRCA) Ab. These mutations are shared across all the Ab lineage members, and the four “prongs” represent the sub-lineages. Conservation of the ^52a^H-D/E-X-D/Q^53^ motif suggests that certain HCDR2 mutations arose in the MRCA Ab and were critical for stabilizing a given HCDR3, then further diversified as the Ab sub-lineages diverged. Most of the prong mutations occurred in the C-term half of HCDR2, HCDR3, and LCDR1, likely due to strong viral selection pressure. The insertions in LCDR1 likely correspond to different N160_glycan2_ recognition strategies.

The overall matured HCDR3 β-motif in the PGT145 family is similar to bovine antibodies, which form an anti-parallel β sheet “stalk” (Wang et al., 2013) that support a globular domain at the turn of the β sheet, thought to recognize antigen. In humans, a cross-reactive influenza antibody called C05 uses a HCDR3 β-hairpin to bind the hemagglutinin receptor binding site and was shown to contain potential HCDR3 stabilizing mutations in HCDR1 and HCDR2 (Ekiert et al., 2012); although its HCDR3 reflects the hammerhead shape of PG9, rather than the long β sheet stalks in bovine antibodies and PGT145. Therefore the structural and sequence analysis reveals that the evolution of these types of antibodies is driven by somatic hypermutation in which substitutions stabilize an extended HCDR, thereby reducing the entropy for binding antigen.

## Discussion

HIV Env is remarkable in its ability to substantially alter its sequence and accommodate a high density of surface glycans and yet still fold and retain its conserved receptor binding and fusion functions. Here we described the structural basis of recognition and neutralization by a class of trimer apex-targeting bnAbs that exploit the intrinsic conservation in the trimer apex that enables Env to undergo receptor-induced conformational changes to attain its fusion active configuration. In the trimer ground state, the V1/V2 regions of the three protomers interact at the trimer apex to hold the gp120 subunits together and mask the co-receptor binding site. These interactions, however, cannot be overly stabilizing, as the V1/V2 region must remodel to enable the V3 base to bind the negatively charged N terminus of the CCR5/CXCR4 co-receptor for viral fusion to proceed (Liu et al., 2008, Rizzuto et al., 1998, Wu et al., 1996). We hypothesize that Env achieves this meta-stable balance by enriching the apex in basic amino acids, a subset of which are also important for co-receptor engagement. The PGT145-family bnAbs then exploit this cationic apex through extended, unusually stabilized HCDR3 loops that contain acidic residues and sulfated tyrosines distributed along the length of the β-hairpin loop, with most charge localized to the apical tip. The HCDR3 stalk is structurally conserved across the PGT145 family yet primarily interacts with a single glycan at N160. The stalk, stabilized by tertiary and quaternary interactions with the other CDR loops, serves as a platform to insert the somewhat variable tip of the β-hairpin into the glycan-shielded trimer apex. The 3-fold axis at the trimer apex comprises a large deep pocket that is permissive to a variety of negatively charged residues at the apical tip of the β-hairpin. This type of Env recognition is unique to this class of antibodies as most bnAb families, such as the VRC01-class (Scharf et al., 2016, Yacoob et al., 2016), PGT121-124 family (Steichen et al., 2016), or even PG9/PG16 (Andrabi et al., 2015), retain a few key specific peptide interactions that are conserved. Overall, our observations demonstrate that the potency of PGT145 is highly dependent on the CDR-stabilized HCDR3 structure and the acidic charge localization in the HCDR3 β-turn. We hypothesize that apex-directed bnAbs like PGT145 require less SHM to achieve breadth and potency because of the less-specific nature of binding to the apex; that is, the specific molecular determinants of binding are less stringent than for other bnAb classes. The steep angle of approach made possible by the long rigidified β-hairpin of HCDR3 also minimizes potential steric block by the hypervariable V1 and V2 loops. Viral fitness relies on preserving a fine balance between the closed and open conformations of the meta-stable, charged apical region of the Env trimer. By targeting these conserved properties, rather than sparsely conserved residues on the surface of Env, the PGT145-family of bnAbs have solved the HIV neutralization puzzle in a particularly creative fashion.

Our structural description of Env in complex with an apex targeting bnAb highlights the importance of an intact trimer apex for eliciting an appropriate immune response to this region. As germline targeting strategies gain more popularity the apex will likely require a trimeric immunogen that has the correct disposition of glycans and positive charges rather than a monomeric scaffold. Most rational vaccine design approaches involve engagement of appropriate germline precursors and biasing SHM to evolve specific molecular contacts with the antigen. The trimer apex presents a different challenge. Here, several mutations that do not contact the antigen are critical for bnAb evolution. One strategy for analyzing germline antibodies capable of evolving these types of responses would be to search for homologous HCDR3-stabilizing residues that reside in the other CDRs. These mutations can also act as early guideposts for evaluating if an immune response is going down the right path.

## Experimental Procedures

### Protein Expression and Purification

Untagged or C-term His_6_-tagged BG505 SOSIP.664 trimers were expressed in HEK293S or 293F cells and affinity purified using a 2G12 IgG cross-linked Sepharose column as described previously (Julien et al., 2013a). The affinity-purified trimers were size exclusion purified using a HiLoad 26/600 Superdex 200 pg column in 20 mM Tris pH 7.4, 150 mM NaCl (1× TBS), unless stated otherwise. The JR-FL EnvΔCT-PGT145 Fab complex was purified as described using PGT145 as the pull-down reagent (Blattner et al., 2014). Antibody IgGs and Fabs were expressed in 293F cells and purified as previously described (Sok et al., 2014) (Supplemental Experimental Procedures).

### Negative Stain EM Data Collection and Processing

All negative stain grids were prepared using 400 Cu mesh carbon coated grids as previously described (Julien et al., 2013b). PGT145 bound complexes were stained using NanoW for ∼30 s, and unliganded trimers were stained using 2% uranyl formate for 45 s to 1 min. The EnvΔCT-PGT145 Fab complex was imaged on a Tecnai T12 coupled with a Tietz PXL 2k × 2k CCD camera, at a magnification of 52,000× resulting in 2.65 Å/pix images. Images were collected using Leginon (Suloway et al., 2005). Particles were processed as previously described (Blattner et al., 2014). Unliganded trimer mutants were imaged on a Tecnai T12 microscope coupled with a Tietz TemCam F416 CMOS detector, at a magnification of 52,000× resulting in 2.05 Å/pix on the specimen plane. Reference-free 2D classes were generated using MSA/MRA (Ogura et al., 2003) to sort the different trimer forms. Because these trimers were SEC purified over a Superdex 200 Increase 10/300 GL column, a large number of the mutants contained a significant portion of particles corresponding to monomers and dimers. Thus monomer and dimer populations were included in the analysis. In the first round of classification, all classes that had more than one particle in the boxed class average were eliminated. From a second round of 2D classification, reference-free 2D class averages were sub-grouped into three populations; (1) “non-native” that includes monomers, dimers, and badly assembled trimers, (2) “native-like closed,” and (3) “native-like total” that includes both the closed (population [2]) and “breathing” (“native-like” but open) trimers (Pugach et al., 2015) (Supplemental Experimental Procedures).

### CryoEM Data Collection and Processing

BG505 SOSIP.664 trimers produced in 293F cells were pre-complexed with 6-molar excess of 3BNC117 Fab overnight at 4°C, and size exclusion purified. To break the pseudo-symmetry of PGT145, PGT145 Fab was pre-complexed with a mouse Fab obtained from a commercial hybridoma cell line ATCC CRL-1757 (1757) that binds the HC of human Fabs (Figures S2A and S2B). To make the BG505 SOSIP.664-3BNC117-PGT145-1757 complex, PGT145 was combined with 1757 Fab (1:2 molar ratio) and size exclusion purified. The PGT145-1757 complex was then incubated with the purified BG505 SOSIP-3BNC117 complex and size exclusion purified. Samples were concentrated and manually frozen in liquid ethane. Data were collected via Leginon (Suloway et al., 2005) on an FEI Titan Krios electron microscope operating at 300 KeV coupled with a K2 Summit direct electron detector camera (Gatan) in counting mode at a magnification of 22,500× resulting in a pixel size of 1.31 Å/pixel, using a total dose of ∼32 e^−^/Å^2^. Data were processed using RELION 1.4b1 (Scheres, 2012). The BG505 SOSIP.664-3BNC117 complex was refined using 22,625 particles with C3 symmetry imposed, to ∼4.4 Å resolution at a Fourier shell correlation (FSC) cut-off of 0.143. 1757 was found to be specific for the constant region of the Fab HC, near the protein G binding site (Derrick and Wigley, 1994) (Figure S2F) allowing for confidence in the PGT145 Fab orientation. The BG505 SOSIP.664-3BNC117-PGT145-1757 and BG505 SOSIP.664-3BNC117-PGT145 classes from 3D sorting were combined, resulting in a total of 65,060 particles that were refined without imposing symmetry to 4.7 Å resolution (FSC = 0.143). A soft edge mask masking out 1757 Fab and PGT145 Fab constant domains was applied for one additional iteration of focused refinement, resulting in the ∼4.3 Å resolution model (FSC = 0.143) (Figure S2D) (Supplemental Experimental Procedures).

### Model Building and Refinement into the CryoEM Maps

The crystal structures of BG505 SOSIP.664 (4TVP) and 3BNC117 Fab (4JPV) were used as templates to generate an initial atomic model using the Modeller plug-in in UCSF Chimera (Pettersen et al., 2004, Webb and Sali, 2016). The resulting model was iteratively fixed and refined in Coot (Emsley et al., 2010) and RosettaRelax (DiMaio et al., 2009) employing Ramachandran constraints. Final models were chosen based on a combination of the Rosetta energy score, MolProbity and clash scores (Chen et al., 2010), and EMRinger score (Barad et al., 2015). Glycans were modeled into the finalized protein model as previously described (Lee et al., 2015), with all glycans being modeled as oligomannose. The protein structure in the BG505-3BNC117 model was used as an initial model to refine the PGT145-bound structure. The sulfated tyrosines in the PGT145 Fab X-ray structure were replaced with regular tyrosines because Rosetta fails to recognize sulfated tyrosines. The complete BG505-3BNC117-PGT145 complex was refined and followed by glycan modeling as was done for the BG505-3BNC117 complex (Supplemental Experimental Procedures).

### Crystallization and X-ray Data Collection

PGT143 and PGT144 Fabs were concentrated to 4-24 mg/mL. Fab samples were screened for crystallization using the 384 conditions of the JCSG Core Suite at both 277 and 293 K using the TSRI/IAVI/JCSG robotic Crystalmation system as described previously (McLellan et al., 2011). Data collection was performed at cryogenic temperature (100 K) at beamline 23-ID of the Argonne Photon Source (APS), using a beam wavelength of 1.033 Å. The diffraction data were indexed, processed and scaled with HKL-2000 (Otwinowski and Minor, 1997) or XDS. Both structures were determined by molecular replacement using Phaser (McCoy et al., 2007) with PGT145 Fab as an initial model (3U1S). Model building and refinement was carried out using Coot-0.7 (Emsley et al., 2010) and Phenix (Adams et al., 2010) (Supplemental Experimental Procedures).

### Surface Plasmon Resonance

SPR analysis of PGT145 Fab binding to His-tagged BG505 SOSIP trimers was analyzed on a Biacore 3000 instrument at 25°C. Glycan knockout mutants were expressed in HEK293F cells unless otherwise indicated. All trimers were immobilized on the chip by His-tag capture, as previously described (Yasmeen et al., 2014). To study interference or enhancement of CD4 and PGT145 binding, sequential binding analyses were also performed as stated previously (Derking et al., 2015) (Supplemental Experimental Procedures).

### Neutralization Assays

For pseudovirus production, we cotransfected HEK293T or 293S cells with an Env encoding and an Env-deficient backbone (pSG3DEnv) plasmids using Fugene 6 (1:2 ratio). Pseudoviruses were harvested 48–72 hr post-transfection, filtered, and titrated for use in neutralization assays. Neutralization was measured in TZM-bl target cells, as described previously (Andrabi et al., 2015) (Supplemental Experimental Procedures).

### Glycan Array Assays

mAbs were screened on a custom high mannose array, consisting of 9 mannosides and 1 control sialylated N-glycan. The 10 amine-linked glycans were covalently immobilized onto custom NHS-ester dendron functionalized glass microscope slides (G3 and G4, ZBiotech). To assess mAb binding, the antibodies were pre-mixed with the detection antibody (anti-human-IgG R-PE). Following 15 min, the pre-complexed antibodies were applied directly to the slide surface and allowed to incubate for 1 hr and then washed. Washed arrays were dried by centrifugation and then scanned for RPE signal on a confocal microarray scanner (Supplemental Experimental Procedures).

### Identification of Key Residues by Regression Analysis

TZM-bl neutralization assay derived IC_50_ values of PGT145 from 135 strains was employed for linear regression analysis. Strains with IC_50_ > 10 μg/mL were classified as escape. Multiple sequence alignment was performed by MUSCLE using default parameters (Edgar, 2004). By comparing the amino acid identity of each residue of interest in each strain to that of BG505, a numeric value was assigned based on an adjusted BLOSUM62 matrix (Henikoff and Henikoff, 1992). For each substitution relative to a given amino acid at BG505, the numeric value was computed by subtracting the substitution score from the self-substitution score. No substitution is represented by a value of 0. A negative value would represent the conservativeness of the substitution, with less conservative being more negative. As a result, the amino-acid sequence for each strain was converted to a list of integers. Combining these sequences in the integer representation generated a matrix. A logistic regression model with L1 regularization was then fit to the matrix with the escape phenotype as the targets. Logistic regression was performed using “linear_model.LogisticRegressionCV” in scikit-learn (Pedregosa et al., 2011) in python. Each residue of interest would be assigned a coefficient. A larger magnitude of coefficient a residue implied more influence it has on the escape phenotype. The absolute value of the coefficient was reported (Supplemental Experimental Procedures).

## Author Contributions

J.H.L, R.A., and J.-P.J. designed the project. J.H.L. performed the EM work and structural analysis. J.-P.J. and L.K. performed the crystallization studies. J.H.L. and C.A.C. performed atomic modeling of the EM structures. R.A., C.S., D.S., and M.P. performed the neutralization assays. A.Y. and P.J.K. performed the SPR studies. R.M. performed the glycan array assays. N.C.W. performed the linear regression analysis. T.N. and C.B. provided reagents. J.H.L., R.A., P.J.K., D.R.B., and A.B.W. analyzed the data. R.A. contributed to figure generation. J.H.L., A.B.W., D.R.B., and I.A.W. wrote the manuscript. All authors were asked to comment on the manuscript.

## Figures and Tables

**Table 1:** X-ray Data Collection and Refinement Statistics

Beamline	PGT143 Fab	PGT144 Fab
	APS 23ID-B	APS 23-ID-D
Wavelength, Å	1.033	1.0332
Space group	P2_1_2_1_2_1_	P1
Unit cell a, b, c (Å)	70.9, 97.6, 126.2	57.6, 67.6, 69.6
α, β, γ (°)	90.00, 90.00, 90.00	82.01, 74.97, 81.28
Resolution (Å)	48.8 – 2.40 (2.44 – 2.40)^a^	40 – 2.9 (3.0 – 2.9)^a^
Completeness	95.1 (87.4)^a^	96.6 (96.9)^a^
Redundancy	3.5 (2.5)^a^	2.1 (2.1)^a^
No. total reflections	113,011	45,828
No. unique reflections	32,477	21,292
I/σ	9.6 (2.2)^a^	5.5 (1.9)^a^
R_sym_	0.11 (0.49)^a^^,^^b^	0.16 (0.39)^a^^,^^b^
R_pim_	0.06 (0.36)^a^	0.14 (0.35)^a^
*CC*_1/2_	97.0 (90.0)^a^	94.8 (59.1)^a^

**Refinement Statistics**		

Resolution (Å)	48.8-2.4	40 – 2.9
No. reflections total/R_free_	30,746/1,652	21,284/1,064
R_cryst_/R_free_	22.9^c^/ 26.7^d^	24.11^c^/28.2^d^
RMSD Bond Length (Å)	0.003	0.0025
RMSD Bond Angles (°)	0.900	0.620
Protein Atoms	6,913	6,856
Wilson B-value (Å^2^)	46.6	38.6
Overall average B-value (Å^2^)	38.6	42.5

**Ramachandran**		

Favored (%)	96.2	95.5
Allowed (%)	3.6	4.0
MolProbity all-atom clashscore	7.4^e^	8.7^e^
PDB ID	5UXQ	5UY3

a Numbers in parentheses refer to the highest resolution shell.

b *R*_sym_ = Σ_*hkl*_Σ_*i*_ | *I*_*hkl,i*_ - < *I*_*hkl*_> | / Σ_*hkl*_Σ_*i*_*I*_*hkl,I*_, where *I*_*hkl,i*_ is the scaled intensity of the *i*^th^ measurement of relection h, k, l, < *I*_*hkl*_> is the average intensity for that reflection, and *n* is the redundancy.

c *R*_cryst_ = Σ_*hkl*_ | *F*_o_ - *F*_c_ | / Σ_*hkl*_ | *F*_o_ | x 100

d *R*_free_ was calculated as for *R*_cryst_, but on a test set comprising 5% of the data excluded from refinement.

e These values were calculated using MolProbity.

## Accession Numbers

Atomic coordinates and structure factors for the X-ray structures have been deposited with the Protein Data Bank under accession codes 5UXQ (PGT143) and 5UY3 (PGT144). The cryoEM maps and models have been deposited with the Electron Microscopy Data Bank and the Protein Data Bank under accession codes EMD-8644, 5V8M (BG505 SOSIP.664-3BNC117) and EMD-8643, 5V8L (BG505 SOSIP.664-3BNC117-PGT145).

## Supplemental Information

Document S1. Figures S1–S7 and Supplemental Experimental Procedures

Document S2. Article plus Supplemental Information
