# Supplementary material for: A Broadly Neutralizing Antibody Targets the Dynamic HIV Envelope Trimer Apex via a Long, Rigidified, and Anionic β-Hairpin Structure
Source: Immunity. 2017 Apr 18;46(4):690–702. doi: 10.1016/j.immuni.2017.03.017 (PMC5400778; doi:10.1016/j.immuni.2017.03.017)
Supplement: Document S1. Figures S1–S7 and Supplemental Experimental Procedures [file mmc1.pdf]

Immunity, Volume *46*

Supplemental Information

A Broadly Neutralizing Antibody Targets the Dynamic HIV Envelope Trimer Apex via a Long, Rigidified, and Anionic β-Hairpin Structure

Jeong Hyun Lee, Raiees Andrabi, Ching-Yao Su, Anila Yasmeen, Jean-Philippe Julien, Leopold Kong, Nicholas C. Wu, Ryan McBride, Devin Sok, Matthias Pauthner, Christopher A. Cottrell, Travis Nieusma, Claudia Blattner, James C. Paulson, Per Johan Klasse, Ian A. Wilson, Dennis R. Burton, and Andrew B. Ward

SUPPLEMENTARY FIGURES

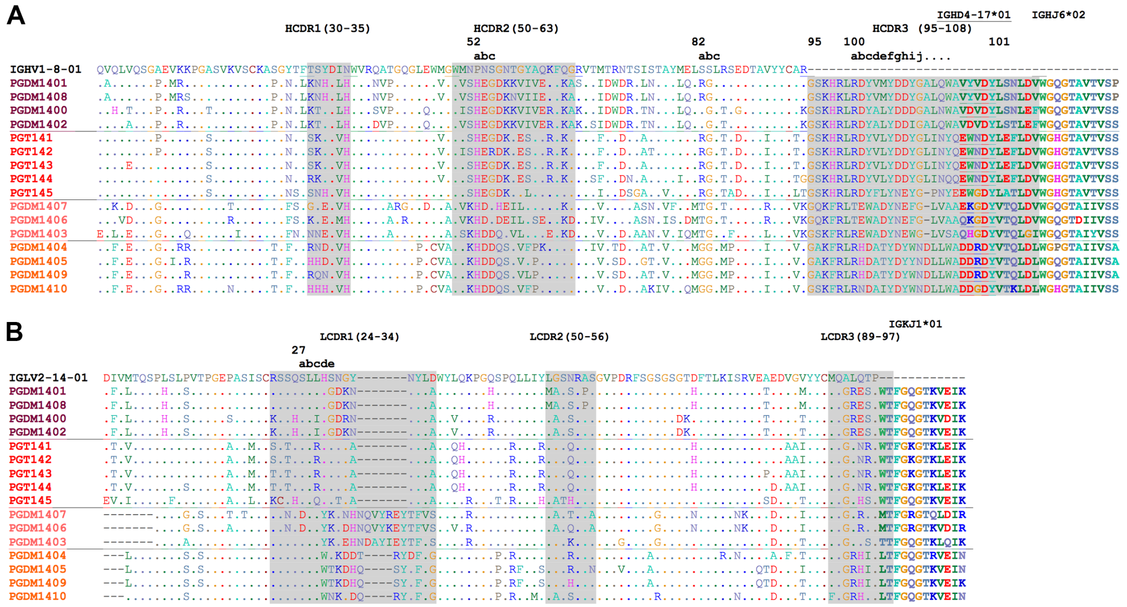

Figure S1. Sequence alignment of PGT145-family Abs. Related to Figure 1 and Table 1.

(A) Sequence alignment of HC variable regions of PGT145-family antibodies. The predicted germline gene is shown at the top. The insertions in CDRs are listed alphabetically (e.g. a,b,c, etc.). Due to the shorter HCDR3 in PGT145, PGDM1407, PGDM1406 and PGDM1403, the insertions beyond 100j are not listed in this figure.

(B) Sequence alignment of LC variable regions of PGT145-family antibodies.

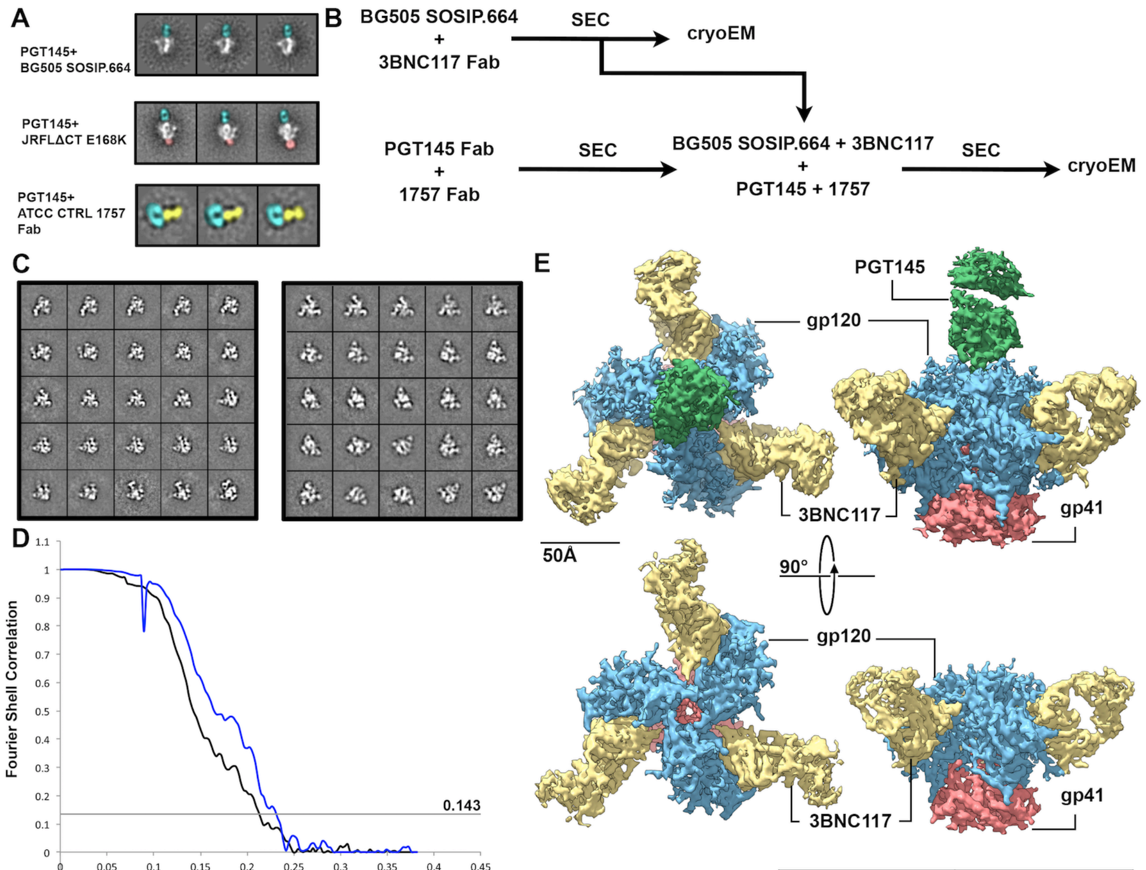

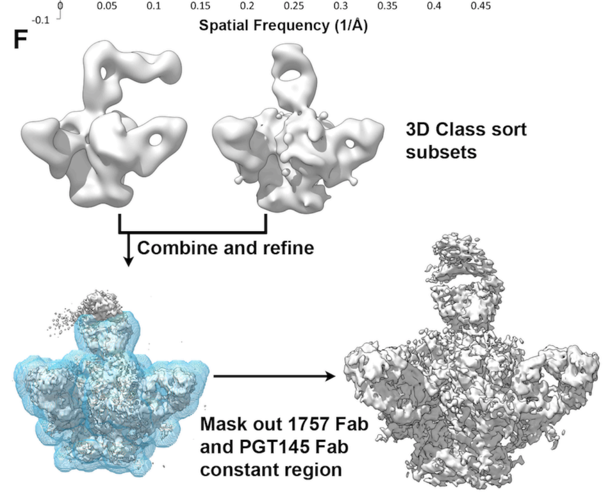

G

	BG505.664 in complex with 3BNC117	BG505.664 in complex with 3BNC117 and PGT145
**Clashscore, all atoms**	5.94 (91th percentile, all resolutions)	6.83 (87th percentile, all resolutions)
**Poor rotamers**	0.14%	0.30%
**Favored rotamers**	99.43%	98.70%
**Ramachandran outliers**	0%	0.19%
**Ramachandran favored**	97.74%	98.07%
**MolProbity score**	1.40 (97th percentile, all resolutions)	1.38 (98th percentile, all resolutions)
**Cβ deviations**	0%	0.04%
**Bad bonds**	0.24%	0.24%
**Bad angles**	0.33%	0.44%
**Cis prolines**	0	2
**EMRinger score**	1.41	1.33

Figure S2. Single particle EM analysis of the 3BNC117-BG505 SOSIP.664 and PGT145-3BNC117-BG505 SOSIP.664 complexes. Related to Figure 1.

(A) Reference free negative stain 2D class averages of BG505 SOSIP.664-PGT145 Fab complex, JR-FL EnvΔCT E168K-PGT145 Fab, and PGT145 Fab-1757 Fab complex. PGT145 and 1757 Fabs are false colored in blue and yellow, respectively. In the JR-FL EnvΔCT sample, the transmembrane domain is colored in pink.

(B) Purification scheme for the two complexes studied by cryoEM.

(C) Reference free 2D class averages of the BG505-3BNC117-PGT145-1757 complex (left), and BG505-3BNC117 complex (right).

(D) Fourier shell correlation curves of the final reconstructions of the 3BNC117-PGT145 (blue), and 3BNC117 (black) bound complexes.

(E) Segmented top (left) and side views (center) of the cryoEM reconstruction of BG505 SOSIP.664-3BNC117-PGT145 (top), and BG505 SOSIP.664-3BNC117 (bottom) complexes, colored according to subunit identity. Scale bar is shown.

(F) Sorting and refinement strategy of the BG505 SOSIP.664-3BNC117-PGT145-1757 complex. The blue mesh corresponds to the mask applied for final focused refinement iterations.

(G) MolProbity and EM Ringer statistics for the refined models.

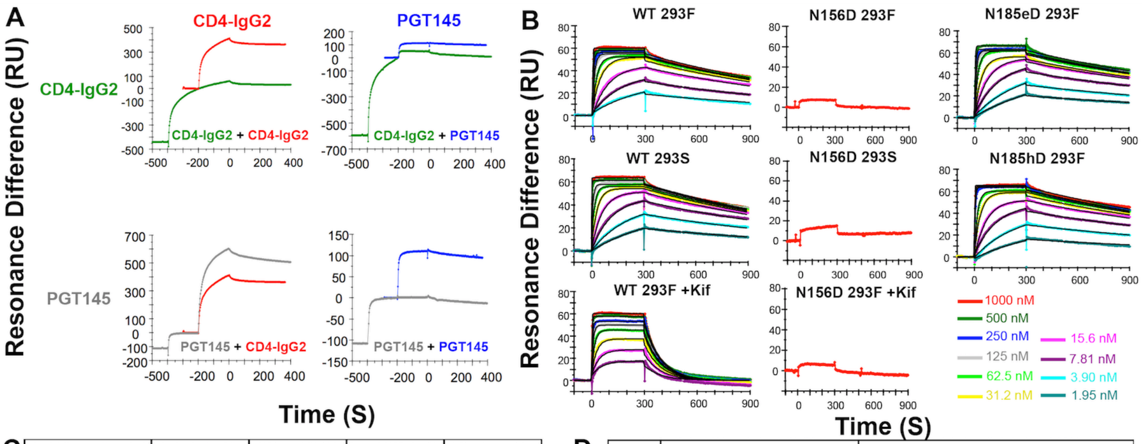

C

	k_{on} (1/Ms)	k_{off} (1/s)	K_d (nM)	S_m
**WT 293F** (n=2)	$6.1 \cdot 10^5$ $\pm 2.5 \cdot 10^4$	$7.7 \cdot 10^{-4}$ $\pm 5.5 \cdot 10^{-5}$	1.3 $\pm 5.0 \cdot 10^{-2}$	0.87 $\pm 2.1 \cdot 10^{-2}$
**WT 293S** (n=2)	$1.1 \cdot 10^6$ $\pm 5.0 \cdot 10^4$	$7.8 \cdot 10^{-4}$ $\pm 2.0 \cdot 10^{-5}$	0.76 $\pm 3.5 \cdot 10^{-2}$	0.90 $\pm 1.8 \cdot 10^{-3}$
**WT 293F Kif** (n=2)	$7.8 \cdot 10^5$ $\pm 1.0 \cdot 10^4$	$1.1 \cdot 10^{-2}$ ± 0	14 $\pm$ 0.50	0.79 $\pm 3.8 \cdot 10^{-3}$
**N185eD 293F** (n=2)	$7.7 \cdot 10^5$ $\pm 5.0 \cdot 10^3$	$6.2 \cdot 10^{-4}$ $\pm 5.0 \cdot 10^{-6}$	0.81 $\pm 1.5 \cdot 10^{-2}$	0.97 $\pm 8.9 \cdot 10^{-3}$
**N185hD 293F** (n=2)	$9.1 \cdot 10^5$ $\pm 1.5 \cdot 10^4$	$6.5 \cdot 10^{-4}$ $\pm 1.5 \cdot 10^{-5}$	0.72 $\pm 5.0 \cdot 10^{-3}$	0.96 $\pm 2.1 \cdot 10^{-3}$

D

1	Man4-O-C_5-NH_2	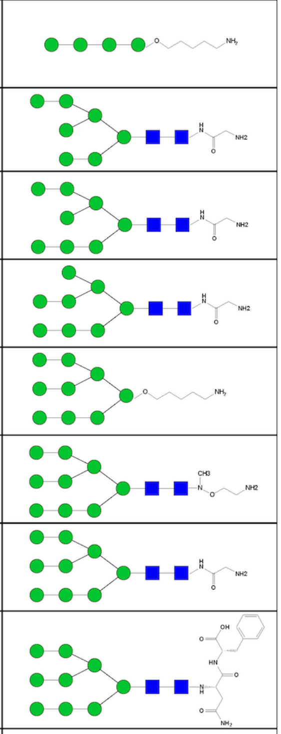
2	Man7-GlcNAc$_2$-Gly	
3	Man8(B)-GlcNAc$_2$-Gly	
4	Man8(C)GlcNAc$_2$-Gly	
5	Man9-O-C_5-NH_2	
6	Man9-GlcNAc$_2$-Oxime-NH_2	
7	Man9-GlcNAc$_2$-Gly	
8	Man9-GlcNAc$_2$-AsnPhe	
9	Man4-Dendron-NH_2	
10	Man9-Dendron-NH_2	

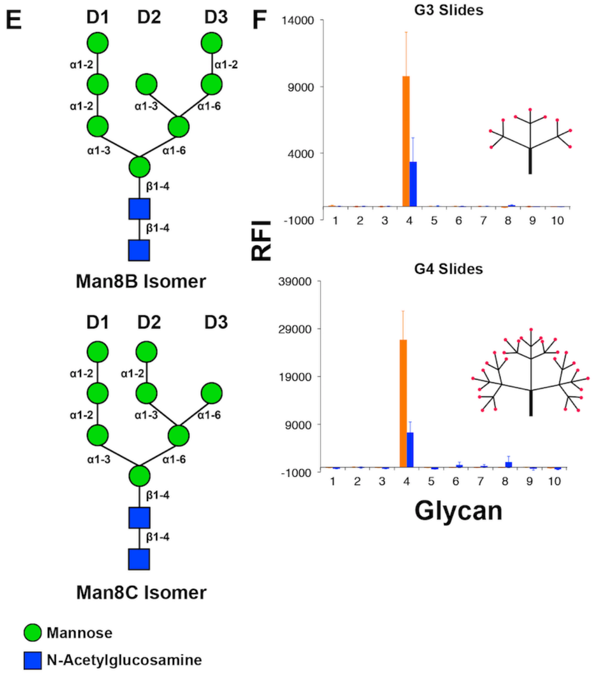

Figure S3. SPR analysis of PGT145 binding. Related to Figures 1 and 2.

(A) SPR sensograms showing association and dissociation phases of binding to PGT145 or CD4IgG2 in the presence of the other.

(B) SPR analysis of PGT145 binding to BG505 SOSIP.664 trimers produced in wild-type glycan producing 293F, 293S, or 293F cells in the presence of Kif, and glycan knockouts at the apex.

(C) SPR kinetic parameters of PGT145 Fab binding to various BG505 SOSIP.664 variants performed in (B). Means of n replicates and ± s.e.m. are given.

(D) The range of glycans tested on the oligomannose glycan array and their structures. The Man_4- and Man_9-Dendron-NH_2 are oligomannose residues pre-attached to a dendron prior to attachment to the G3 or G4 dendrimer slides.

(E) Definition of the M8B and M8C isomers and oligomannose branching nomenclature.

(F) PGT145 (blue) and PGDM1400 (orange) binding to the glycans shown in (E) on G3 (top) or G4 (bottom) slides. Error bars indicate s.e.m. To the right of the bar graphs show the dendrimer branching scheme for the G3 and G4 slides, with red dots indicate where glycans would be attached. Dendron image is not to scale.

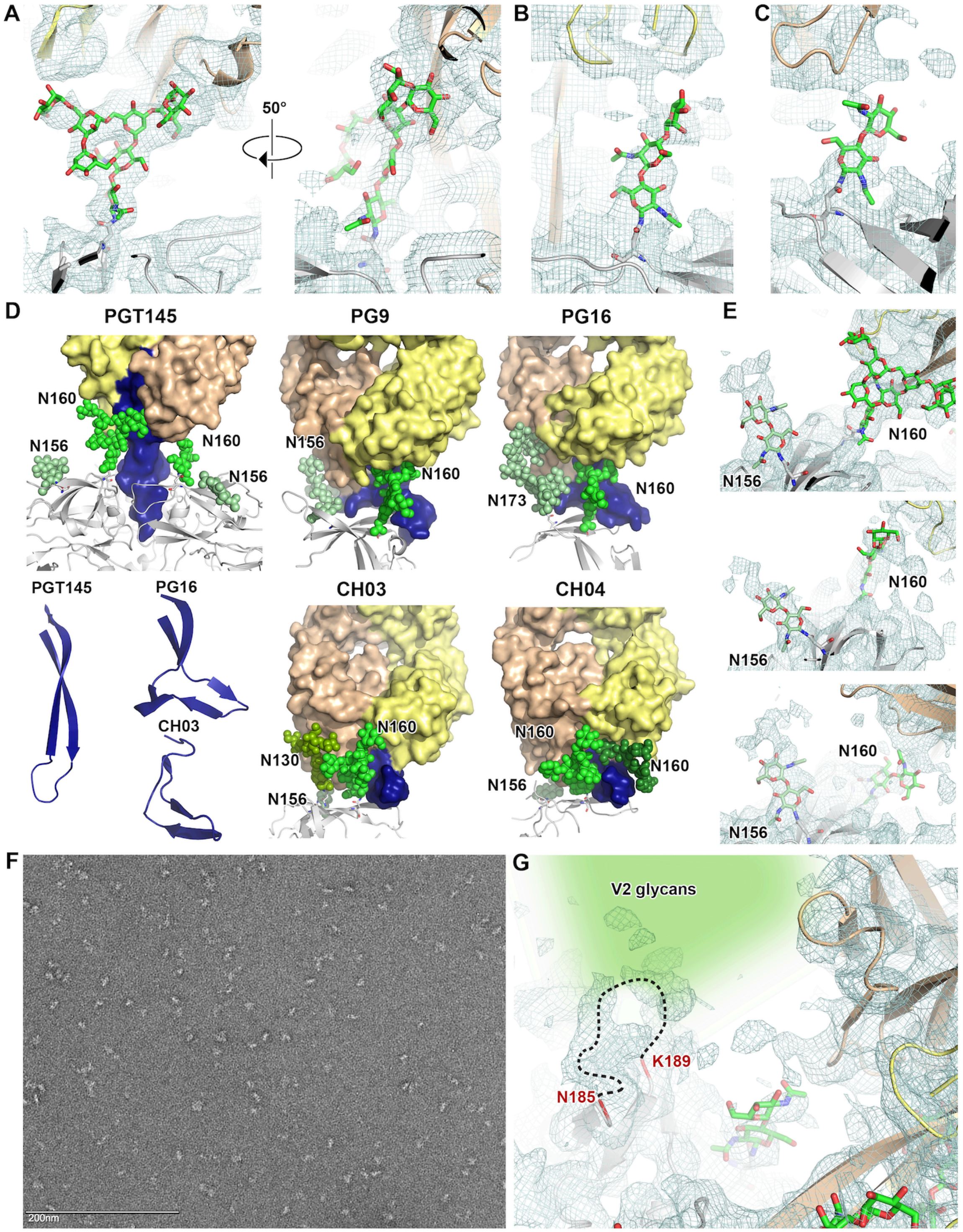

Figure S4. N160 glycan densities and comparison of glycan recognition by apex bnAbs. Related to Figure 3.

The modeled N160 glycans (green) and the cryoEM map (blue mesh). PGT145 HC and LC are shown in tan and yellow, respectively. Gp120 is shown in gray.

(A) $\mathrm{N160}_{\mathrm{glycan1}}$ in the BG505-PGT145 complex map.

(B) $N160_{glycan2}$ in the BG505-PGT145 complex map.

(C) $N160_{glycan3}$ in the BG505-PGT145 complex map.

(D) V2 binding antibodies PGT145, PG9 (3U4E), PG16 (4DQO), CH03 (5ESV) and CH04 (5ESZ), and their interaction with apex glycans. The N173 glycan is a compensatory glycan for N156. CH04 interacts with an additional glycan (dark green) predicted to represent the N160 glycan from an adjacent protomer. A comparison of the HCDR3 structural conformations is shown in the bottom left. The light and heavy chains are colored as in (A), with the HCDR3 shown in blue.

(E) The N156 glycans face away from PGT145. The figure is colored as in (A). The EM map is contoured at $\sigma=5.5$.

(F) A negative stain EM micrograph of BG505 SOSIP.664 N156D trimers. Very few intact trimers are visible in the image.

(G) The dashed line indicates the complete trajectory of the V2 loop. The two glycans in V2 of BG505 likely occupy space (shaded region) near the PGT145 antibody (tan). The EM map is contoured at $\sigma=5.5$ and glycans are shown as green sticks.

A

BG505.Env.C2	PGDM1400	PGDM1401	PGDM1404	PGDM1406	PGDM1409	PGT143	PGT145	PG9	CH01	CAP256.09	VRC01
K121A	3.6	608.7	>3	NA	>17	>353	>302	0.6	0.2	9.0	0.642
V127A	>613	>1072	>3	NA	>17	>353	>302	12.9	17.3	>226	1.535
N156K	144.0	>1072	>3	NA	>17	>353	>302	44.5	17.3	>226	0.967
N160K	>613	>1072	>3	NA	>17	>353	>302	>309	17.3	>226	0.987
N160A	>613	>1072	>3	NA	>17	>353	>302	>309	17.3	>226	0.223
M161A	2.5	15.9	>3	NA	>17	22.2	60.4	1.2	0.6	0.2	0.553
T162A	>613	>1072	>3	NA	>17	>353	>302	>309	17.3	>226	0.674
E164A	0.7	0.8	>3	NA	10.9	0.8	2.7	0.5	0.7	33.7	0.705
L165A	2.4	923.0	>3	NA	>17	152.4	167.1	0.5	0.3	>226	0.421
R166A	>613	>1072	>3	NA	>17	>353	>302	0.8	0.7	>226	1.225
D167A	>613	>1072	>3	NA	>17	>353	>302	0.5	0.3	>226	0.422
K168A	48.3	556.7	>3	NA	>17	155.3	274.8	18.1	4.3	4.3	0.579
K169A	75.9	12.2	>3	NA	>17	3.0	256.2	1.7	2.9	>226	0.942
I307A	0.9	0.7	>3	NA	>17	2.0	0.3	0.7	0.4	9.4	1.043
I420A	1.8	3.2	>3	NA	8.6	3.3	3.8	0.9	1.1	2.4	0.937
I423A	0.6	0.2	>3	NA	16.5	1.2	1.9	1.7	1.7	11.7	0.902

B

Mutation	% Non-Native	% Native-like Closed	% Native-like Total
I420A	100	0	0
L165A	80	19	20
D167A	70	26	30
I307A	58	42	42
I432A	46	36	54
K169A	39	11	61
M161A	38	34	62
T162A	36	53	64
P124A	34	24	66
K121A	30	25	70
R166A	28	56	72
V127A	26	43	74
T123A	21	59	79
WT	19	46	81
E164A	11	40	89
N160A	0	81	100
K168A	0	76	100

C

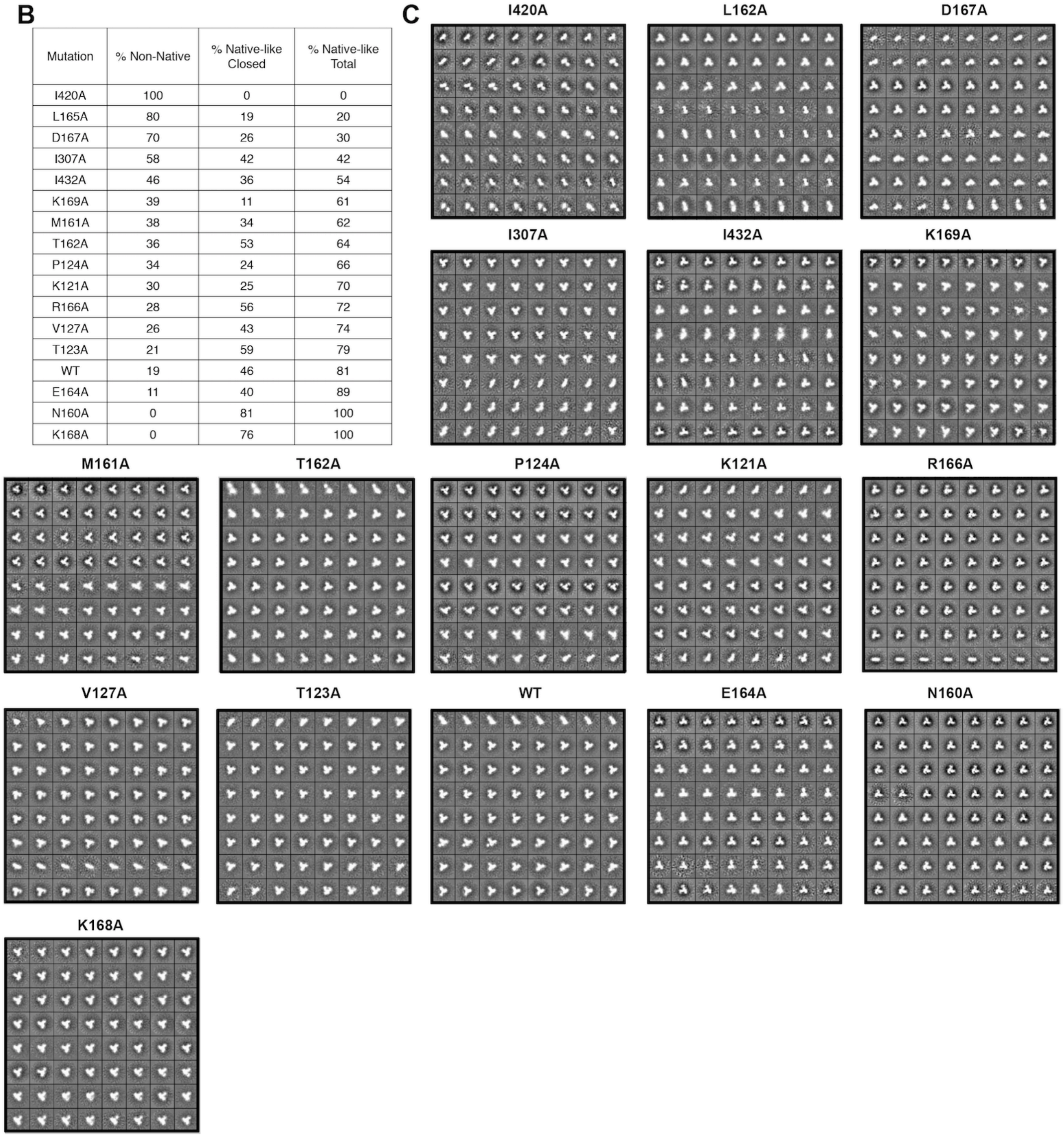

Figure S5. Trimer apex epitope mutations and the effect of point mutations on trimer integrity. Related to Figure 3.

(A) Fold changes in neutralization IC_{50} measured in Figure 2G, relative to the WT BG505 pseudovirus.

(B) Percentage population of open or closed-native like trimers, and non-native trimers (including monomers and dimers) observed in each of the BG505 mutants by negative stain EM, listed in order of lowest to highest % total native-like trimers.

(C) Equivalent point mutations as those in the BG505 pseudoviruses in (A) made in soluble BG505 SOSIP.664 trimers analyzed by negative stain EM. Shown in the same order as listed in (B).

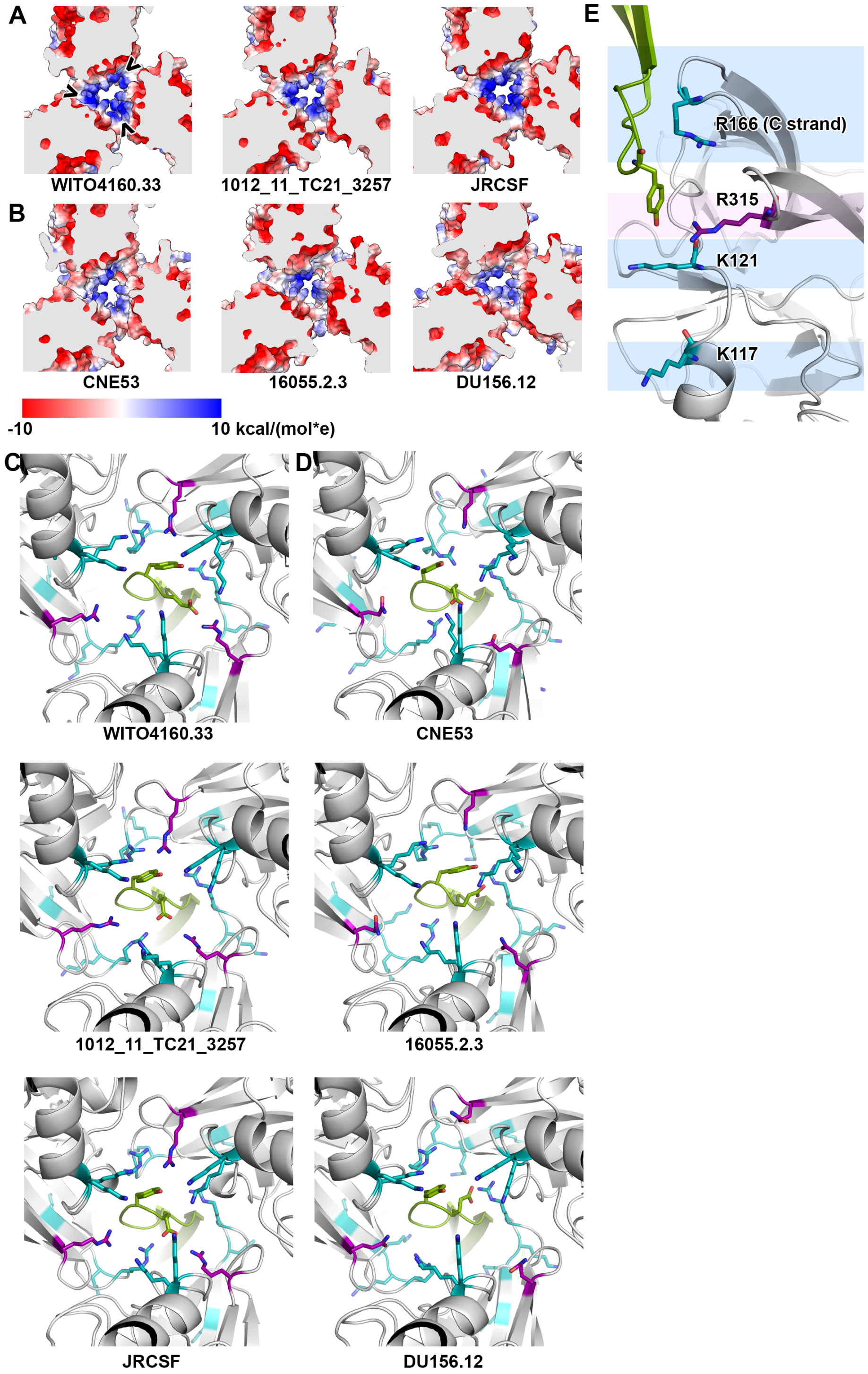

Figure S6. Clade B trimers have an additional electropositive layer in the trimer apex core. Related to Figure 4.

(A) Coulombic potential maps of clade B gp120 trimer homology models relaxed in the presence of PGT145 Fab shows the electrostatic potential at the core of the apex.

(B) Coulombic potential maps of clade C gp120 homology models.

(C) Close up of the regions shown in (A) in the presence of PGT145 HC (green), with positively charged gp120 residues at or close to the PGT145-epitope shown in teal. R315 is shown in purple, and is near the acidic HCDR3 tip side chains (E100h, Y100i, green sticks).

(D) Close up of the clade C apices shown in (B). The clade C trimers have a Q315 residue in place of R315.

(E) The R315 results in an extra electropositive layer (purple), in addition to the conserved C-strand, K121 and K117 layers (blue). Model shown is that of WITO4160.33. The HCDR3 of PGT145 is shown in green.

SUPPLEMENTARY EXPERIMENTAL PROCEDURES

Protein Expression and Purification

Untagged or C-term His_6-tagged BG505 SOSIP.664 trimers were expressed in HEK293S or 293F cells and affinity purified using a 2G12 IgG cross-linked sepharose column as described previously (Julien et al., 2013). Briefly, cells were co-transfected with BG505 SOSIP.664 and furin using a ratio of 4:1, and PEI as the transfection reagent. In some cases, 20 uM of kifunensine was added per L of media at time of transfection. After 5-6 days, the cells were harvested and the supernatant passed over a 2G12 affinity column. Trimers were eluted with 3 M $MgCl_2$ pH 7.4, and dialyzed into 50 mM Tris pH 8, 500 mM NaCl and stored at 4 °C until further use. The affinity-purified trimers were SEC purified a few days prior to structural or biophysical experiments using a HiLoad 26/600 Superdex 200 pg column (GE Healthcare) in 20 mM Tris pH 7.4, 150 mM NaCl (1x TBS), unless stated otherwise.

Antibody IgGs and Fabs were expressed in HEK293F cells with a ratio of 2:1 (HC:LC), using PEI. PGT145, PGDM1400, PGT144 and PGT143 IgGs or Fabs were co-transfected with TPST1 using a ratio of 2:1:1 (HC:LC:TPST1) to ensure tyrosine sulfation. After 5-6 days, cells were harvested and supernatant collected. IgGs were purified using a 5 mL MAb select column (GE Healthcare), and dialyzed into 1x TBS pH 7.4. Fabs were purified using Kappa select column (GE Healthcare). Correctly formed Fabs were separated from LC-LC dimers using a MonoS cation exchange column (GE Healthcare) in 20 mM NaOAc pH 5.6, via a gradient buffer of 20 mM NaOAc pH 5.6, 1M KCl. The correct Fab heterodimer fractions were pooled and further purified by SEC through a Superdex 200 Increase GL 10/30 column (GE Healthcare) in 1x TBS pH 7.4. For PGT143 and PGT144, multiple peaks corresponding to different tyrosine sulfation levels were observed. Protein from each peak was separately purified using size exclusion chromatography with a Superdex 200 10/300 column (GE Healthcare) for crystallization.

Expression and Purification of EnvΔCT-PGT145 Complex

JR-FL EnvΔCT containing the E168K mutant was expressed in 293F cells by co-transfecting the Env gene containing pSVIIIenv vector with Tat at a ratio of 1:3 using 293Fectin. The JR-FL EnvΔCT-PGT145 Fab complex was purified as previously described using PGT145 as the pull-down reagent (Blattner et al., 2014).

Negative Stain EM Data Collection and Processing

All negative stain grids were prepared using 400 Cu mesh carbon coated grids, glow discharged at 20 mAu for 30 seconds prior to usage. All samples were stained as follows: 3 µL of sample per grid, followed by staining with 3 uL NanoW (Nanoprobes) for ~30 sec (for PGT145

bound complexes), or 3 µL 2% uranyl formate (UF, for unliganded trimers) for 45 sec to 1 min. The JR-FL EnvΔCT-PGT145 Fab complex was diluted 1:50 using 1X TBS pH 7.4 without detergent immediately adsorption to grids, and stained using NanoW. All unliganded trimers were diluted to ~0.01 mg/mL with 1X TBS pH 7.4, and stained with 2% UF.

The EnvΔCT-PGT145 Fab complex was imaged on a Tecnai T12 coupled with a Tietz PXL 2k x 2k CCD camera, at a magnification of 52,000x resulting in 2.65 Å/pix images. Images were collected in Leginon (Suloway et al., 2005) using a total dose of ~32 $e^{-}/Å^{2}$ and defocus ranging between 0.5 and 1.0 µm. Particles were picked and stacked using the Appion pipeline (Lander et al., 2009). Reference-free 2D class averages were generated using sxali2d in the Sparx software suite and clustered using Adapt (Frank et al., 1992; Ogura et al., 2003; Ramey et al., 2009).

Unliganded trimer mutants were imaged on a Tecnai T12 microscope coupled with a Tietz TemCam F416 CMOS detector, at a magnification of 52,000x resulting in 2.05 Å/pix on the specimen plane. Images were collected at 1 µm defocus, and particles were stacked as described above. Reference-free 2D classes were generated using MSA/MRA (Ogura et al., 2003) to sort the different trimer forms. Because these trimers were SEC purified over a Superdex 200 Increase 10/300 GL column (GE Healthcare), a large number of the mutants contained a significant portion of particles that corresponded to monomers/dimers. Thus monomer/dimer populations were included in the analysis. In the first round of classification, all classes that had more than one particle in the boxed class average was eliminated. From a second round of 2D classification, reference-free 2D class averages were sub-grouped into three populations; [1] "non-native" that includes monomers, dimers and badly assembled trimers, [2] "native-like closed" and [3] "native-like total" that includes both the closed (population [2]) and "breathing" ("native-like" but open) trimers (Pugach et al., 2015).

CryoEM data collection and processing

BG505 SOSIP.664 trimers produced in 293F cells were pre-complexed with 6-molar excess of 3BNC117 Fab (1 trimer: 6 Fabs) overnight at 4 °C, and purified over a Superose 6 increase column (GE Healthcare). To break the pseudo-symmetry of PGT145, PGT145 Fab was pre-complexed with a mouse Fab obtained from a commercial hybridoma cell line ATCC CRL-1757 (hereon referred to as 1757) that binds the HC of human Fabs (Figures S2A-B). To make the BG505 SOSIP.664-3BNC117-PGT145-1757 complex, PGT145 was combined with 1757 Fab (1:2 molar ratio) and purified over a Superdex 200 increase column (GE Healthcare). The PGT145-1757 complex was then incubated with previously purified BG505 SOSIP-3BNC117 complex, and SEC purified over a Superose 6 increase column in 1x TBS pH 7.4 (GE Healthcare). BG505 SOSIP.664-3BNC117 was concentrated to ~1 mg/mL and supplemented with 0.005%

DDM just prior to freezing. BG505 SOSIP.664-3BNC117-PGT145-1757 was concentrated to ~0.4 mg/mL and supplemented with 0.02% amphipole A8-35 (Anatrace) prior to freezing. The 3 µL of the sample was adsorbed onto 5 second Ar/O_2 plasma cleaned CF-2/2-4C C-Flat holey grids, blotted, then frozen by manual plunging into liquid ethane. Data were collected on an FEI Titan Krios electron microscope operating at 300 KeV coupled with a K2 Summit direct electron detector camera (Gatan) in counting mode in Leginon, at a magnification of 22,500x resulting in a pixel size of 1.31 Å/pixel, using a total dose of ~32 $e^-/Å^2$. For the BG505 SOSIP.664-3BNC117 complex, a total of 1431 images were collected using a nominal defocus range of 1.2~3 µm. For the BG505 SOSIP.664-3BNC117-PGT145-1757 complex, a total of 1946 images were collected using a nominal defocus range of 1.22-2.5 µm. Data were processed as previously described (Lee et al., 2015) with using RELION 1.4b1, with the exception of the Fab constant domain-masking step in refining the BG505 SOSIP.664-3BNC117 complex. The BG505 SOSIP.664-3BNC117 complex was refined using 22,625 particles with C3 symmetry imposed, to ~4.4 Å resolution at a Fourier shell correlation (FSC) cut-off of 0.143. 1757 was found to be specific for the constant region of the Fab HC, near the protein G binding site (Derrick and Wigley, 1994) (Figure S2F) allowing for confidence in the PGT145 Fab orientation. The BG505 SOSIP.664-3BNC117-PGT145-1757, and BG505 SOSIP.664-3BNC117-PGT145 classes from 3D sorting were combined, resulting in a total of 65,060 particles that were refined without imposing symmetry to 4.7 Å resolution (FSC=0.143). A soft edge mask masking out 1757 Fab and PGT145 Fab constant domains was applied for one additional iteration of focused refinement, resulting in the ~4.3 Å resolution model (FSC=0.143) (Figure S2D).

Model building and Refinement into the CryoEM Maps

The crystal structures of BG505 SOSIP.664 (4TVP) and 3BNC117 Fab (4JPV) were used as templates to generate an initial atomic model using the Modeller plug-in in UCSF Chimera (Pettersen et al., 2004; Webb and Sali, 2016). Each domain was independently docked into the EM density map as a rigid body. The resulting The model was iteratively fixed and refined in Coot (Emsley et al., 2010) and RosettaRelax (DiMaio et al., 2009) employing Ramachandran constraints. Final models were chosen based on a combination of the Rosetta energy score, MolProbity and clash scores (Chen et al., 2010), and EMRinger score (Barad et al., 2015). Glycans were modeled into the finalized protein model as previously described (Lee et al., 2015), with all glycans being modeled as oligomannose. The protein structure in the BG505-3BNC117 model was used as an initial model to refine the PGT145-bound structure. Three copies of the BG505 gp140-3BNC117 subunit of the complex and the Fab variable region of PGT145 were rigid body docked into the BG505-PGT145 EM map and refined as described above. The sulfated tyrosines in the PGT145 Fab X-ray structure were replaced with regular tyrosines because

Rosetta fails to recognize sulfated tyrosines. The complete BG505-3BNC117-PGT145 complex was refined and followed by glycan modeling as was done for the BG505-3BNC117 complex.

Homology Modeling and Refinement

Homology models of various gp120s from clade B and C viruses were generated using SWISS-MODEL (Biasini et al., 2014), with 5CEZ (Garces et al., 2015) as the initial model, as this is the highest resolution Env structure in the PDB. Three copies of the gp120s and PGT145 Fab were docked into a 10 Å low-pass filtered model of the BG505 SOSIP.664-PGT145 complex. The docked pieces were relaxed by RosettaRelax to generate 159 models per gp120. The lowest energy models were analyzed. The electrostatic potential maps were generated in Chimera (Pettersen et al., 2004).

Crystallization

PGT143 and PGT144 Fabs containing different tyrosine sulfation levels were separated by MonoS ion exchange chromatography (GE Healthcare) and concentrated to 4-24 mg/mL. Fab samples were screened for crystallization using the 384 conditions of the JCSG Core Suite (Qiagen) at both 277 and 293 K using the TSRI/IAVI/JCSG robotic Crystalmation system (Rigaku) as described previously (McLellan et al., 2011). After approximately 3 days at 20°C, crystals of PGT143 Fab with no tyrosine sulfation formed in 65% (v/v) 2-methyl-2,4-pentanediol and 0.1 M Tris, pH 8.0 (JCSG Core Suite 4, well C11). Tyrosine sulfated PGT143 did not crystallize in any of the screened conditions. Only PGT144 Fab with one sulfated tyrosine crystallized, and the best diffracting crystals were obtained from crystals grown in JCSG Core Suite, well F8.

X-ray Data collection

Crystals were cryo-protected with glycerol (PGT143: 25%, PGT144: 20%) prior to flash freezing in liquid nitrogen. Data collection was performed at cryogenic temperature (100 K) at beamline 23-ID of the Argonne Photon Source (APS), using a beam wavelength of 1.033 Å. The diffraction data The optimized, hexagonal PGT143 Fab crystals diffracted to 2.4 Å and the diffraction data were indexed, processed and scaled with HKL-2000 (Otwinowski and Minor, 1997) to a completeness of 95.1% with an overall R_{sym} of 11% (49% in the high resolution shell). The optimized PGT144 Fab crystals diffracted to 2.9 Å and the diffraction data were indexed, processed and scaled with XDS to an overall completeness of 96.6% and R_{sym} of 16% (Kabsch, 2010).

Crystal Structure Refinement

Both structures were determined by molecular replacement using Phaser (McCoy et al., 2007) with PGT145 Fab as an initial model (3U1S). Model building was carried out using Coot-0.7 (Emsley et al., 2010) and refinement was implemented with Phenix (Adams et al., 2010). Final R_{cryst}/R_{free} values for PGT143 and PGT144 Fabs are 22.9%/26.7% and 24.1%/28.2%, respectively.

Surface Plasmon Resonance

SPR analysis of PGT145 Fab binding to His-tagged BG505 SOSIP trimers was analyzed on a Biacore 3000 instrument at 25°C. Glycan knockout mutants were expressed in 293F cells unless otherwise indicated. All trimers were purified on a 2G12-affinity column, and immobilized on the chip by His-tag capture, as previously described (Yasmeen et al., 2014). In brief, anti-histidine antibody (GE Healthcare) was amide-coupled to the dextran of a CM5 chip up to a level of 15000 RU. Trimers were captured to yield R_L = 520 RU (s.d. < 1.3 %). Throughout, HBS-EP (10 mM HEPES pH 7.4, 150 mM NaCl, 3 mM EDTA, 0.002 % P20 surfactant) was used as running buffer. The maximum flow rate (50 µL/min) was used during analyte binding to minimize mass-transport limitation, the near absence of which was confirmed by global fits of mass transfer coefficients. Association was monitored for 5 min and dissociation for 10 min, the Fab being titrated from 1000 to 2 nM in consecutive cycles. After each cycle, the capture-antibody surface was regenerated by an injection of 10 mM glycine pH 2 for 120 s at a flow rate of 30 µL/min. The signals from both 0-analyte injections and the parallel control channel were subtracted. A Langmuir model (Biavaluation, GE Healthcare) was fitted globally to the binding data to derive the kinetic parameters. S_m values were based on the highest analyte concentrations, yielding the highest T values for R_{max}. All reported binding parameters were significant (T > 10).

To study interference or enhancement of CD4 and PGT145 binding, sequential binding analyses were also performed. The association and dissociation phases of binding to trimers immobilized as above were monitored for 200 s each. PGT145 IgG and CD4-IgG2 were injected at 500 nM, sequentially (at times 0 and 200 s in a single cycle, both at flow rates of 30 µL/min), as described (Derking et al., 2015). A second injection of PGT145 resulted in a residual binding of 2%, relative to the first PGT145 injection; correspondingly, for CD4-IgG2 after a first injection of the CD4-IgG2, the residual binding was 14%, showing that the first injection nearly saturated the respective binding sites. The relative residual binding of PGT145 after CD4-IgG2 injection, or vice versa, was calculated analogously in %, as the binding of analyte 2 after pre-binding of analyte 1, relative to the binding of analyte 2 on its own.

Neutralization Assays

Amino acid substitution mutations in the antibody and HIV envelope encoding plasmids

were incorporated by QuikChange site-directed mutagenesis kit (Stratagene) according to the manufacturer's instructions. For pseudovirus production, we cotransfected HEK293T or 293S cells with an Env encoding and an Env-deficient backbone (pSG3DEnv) plasmids with the transfection reagent Fugene 6 (1:2 ratio) (Promega). Pseudoviruses were harvested 48–72 hr post-transfection, filtered and titrated for use in neutralization assays. Neutralization was measured in TZM-bl target cells, as described previously (Andrabi et al., 2015).

Glycan Array Assays

mAbs were screened on a custom high-mannose array, consisting of 9 mannosides and 1 control sialylated N-glycan. The 10 amine-linked glycans were covalently immobilized onto custom NHS-ester dendron functionalized glass microscope slides (G3 and G4, ZBiotech) using a MicroGridII robotic array printer (Digilab Global) equipped with Stealth SMP4B microarray pins (Telechem). Compounds were diluted to 100 µM and spotted in replicates of 6 and, following 1 h of humidification, were washed in blocking buffer (50 mM ethanolamine in 50 mM borate buffer, pH 9.2) to remove any unbound compound and quench remaining NHS-ester residues. Slides were stored at -20°C prior to use. To assess mAb binding, the antibodies were pre-mixed with the detection antibody (anti-human-IgG R-PE, Jackson Immuno) at a ratio of 2:1, 30 ug/mL and 15 µg/mL, respectively. Following 15 min, the pre-complexed antibodies were applied directly to the slide surface and allowed to incubate for 1 h and then washed. Arrays were washed using three exchanges of 0.05% Tween-20 in 1x PBS, three exchanges of 1x PBS and finally, three exchanges of deionized water. Washed arrays were dried by centrifugation and scanned for R-PE signal on a confocal microarray scanner (Innoscan 1100AL, Innopsys). The resultant images were analyzed using Mapix (Innopsys) and mean signal minus background values for each spotted compound were calculated and plotted using MS Excel.

Identification of Key Residues by Regression Analysis

TZM-bl neutralization assay derived IC_{50} values of PGT145 from a total of 106 strains was obtained from Sok *et al.* (Sok et al., 2014). An additional dataset of IC_{50} values from a total of 65 strains against 293T produced pseudoviruses was obtained from the neutralization assay performed here (Figure 2A), and from Walker *et al.* (Walker et al., 2011) for which the Env sequences were available. In these two datasets, 37 strains overlapped. Because BG505 was not a part of either panel, the PGT145 IC_{50} against BG505 was obtained from the data shown in Fig. 3E. Subsequently, IC_{50} from 135 strains was employed for downstream analysis. For those strains that were included in both datasets, IC_{50} was obtained by averaging between two datasets. Strains with IC_{50} > 10 µg/mL were classified as escape. Multiple sequence alignment was performed by MUSCLE using default parameters (Edgar, 2004). The residues of interest

were extracted. By comparing the amino acid identity of each residue of interest in each strain to that of BG505, a numeric value was assigned based on an adjusted BLOSUM62 matrix (Henikoff and Henikoff, 1992). For each substitution relative to a given amino acid at BG505, the numeric value was computed by subtracting the substitution score from the self-substitution score. For example, at a given residue, if a strain had the same amino acid identity as that of BG505, a value of 0 would be assigned for that particular residue in that strain. If the amino acid identity were different from that of BG505, a negative value would be assigned. This negative value would represent the conservativeness of the substitution, with less conservative being more negative. As a result, the amino-acid sequence for each strain was converted to a list of integers that has the same length as the amino acid sequence string of interest. Combining these sequences in the integer representation generated a matrix. A logistic regression model with L1 regularization was then fit to the matrix with the escape phenotype as the targets. Logistic regression was performed using "linear_model.LogisticRegressionCV" in scikit-learn (Pedregosa et al., 2011) in python. Each residue of interest would be assigned a coefficient. A larger magnitude of coefficient a residue implied more influence it has on the escape phenotype. The absolute value of the coefficient was reported. The global Env sequence conservation information was derived from an alignment of 25,946 sequences across multiple clades from the Los Alamos National Laboratory HIV database.

SUPPLEMENTAL REFERENCES

Adams, P.D., Afonine, P.V., Bunkoczi, G., Chen, V.B., Davis, I.W., Echols, N., Headd, J.J., Hung, L.W., Kapral, G.J., Grosse-Kunstleve, R.W., *et al.* (2010). PHENIX: a comprehensive Python-based system for macromolecular structure solution. Acta Crystallogr., Sect: D Biol. Crystallogr. *66*, 213-221.

Andrabi, R., Voss, J.E., Liang, C.H., Briney, B., McCoy, L.E., Wu, C.Y., Wong, C.H., Poignard, P., and Burton, D.R. (2015). Identification of common features in prototype broadly neutralizing antibodies to HIV envelope V2 apex to facilitate vaccine design. Immunity *43*, 959-973.

Barad, B.A., Echols, N., Wang, R.Y., Cheng, Y., DiMaio, F., Adams, P.D., and Fraser, J.S. (2015). EMRinger: side chain-directed model and map validation for 3D cryo-electron microscopy. Nature Methods *12*, 943-946.

Biasini, M., Bienert, S., Waterhouse, A., Arnold, K., Studer, G., Schmidt, T., Kiefer, F., Gallo Cassarino, T., Bertoni, M., Bordoli, L., and Schwede, T. (2014). SWISS-MODEL: modelling protein tertiary and quaternary structure using evolutionary information. Nucleic acids research *42*, W252-258.

Blattner, C., Lee, J.H., Sliepen, K., Derking, R., Falkowska, E., de la Pena, A.T., Cupo, A., Julien, J.P., van Gils, M., Lee, P.S., *et al.* (2014). Structural delineation of a quaternary, cleavage-dependent epitope at the gp41-gp120 interface on intact HIV-1 Env trimers. Immunity *40*, 669-680.

Chen, V.B., Arendall, W.B., 3rd, Headd, J.J., Keedy, D.A., Immormino, R.M., Kapral, G.J., Murray, L.W., Richardson, J.S., and Richardson, D.C. (2010). MolProbity: all-atom structure validation for macromolecular crystallography. Acta Crystallogr., Sect: D Biol. Crystallogr. *66*, 12-21.

Derking, R., Ozorowski, G., Sliepen, K., Yasmeen, A., Cupo, A., Torres, J.L., Julien, J.P., Lee, J.H., van Montfort, T., de Taeye, S.W., *et al.* (2015). Comprehensive antigenic map of a cleaved soluble HIV-1 envelope trimer. PLoS Pathog. *11*, e1004767.

Derrick, J.P., and Wigley, D.B. (1994). The third IgG-binding domain from streptococcal protein G. An analysis by X-ray crystallography of the structure alone and in a complex with Fab. J. Mol. Biol. *243*, 906-918.

DiMaio, F., Tyka, M.D., Baker, M.L., Chiu, W., and Baker, D. (2009). Refinement of protein structures into low-resolution density maps using rosetta. J. Mol. Biol. *392*, 181-190.

Edgar, R.C. (2004). MUSCLE: multiple sequence alignment with high accuracy and high throughput. Nucleic Acids Res. *32*, 1792-1797.

Emsley, P., Lohkamp, B., Scott, W.G., and Cowtan, K. (2010). Features and development of Coot. Acta Crystallogr., Sect: D Biol. Crystallogr. *66*, 486-501.

Frank, J., Penczek, P., and Liu, W. (1992). Alignment, classification, and three-dimensional reconstruction of single particles embedded in ice. Scanning Microsc. Suppl. *6*, 11-20; discussion 20-12.

Garces, F., Lee, J.H., de Val, N., Torrents de la Pena, A., Kong, L., Puchades, C., Hua, Y., Stanfield, R.L., Burton, D.R., Moore, J.P.*, et al.* (2015). Affinity maturation of a potent family of HIV antibodies Is primarily focused on accommodating or avoiding glycans. Immunity *43*, 1053-1063.

Henikoff, S., and Henikoff, J.G. (1992). Amino acid substitution matrices from protein blocks. Proc. Natl. Acad. Sci. USA *89*, 10915-10919.

Julien, J.P., Cupo, A., Sok, D., Stanfield, R.L., Lyumkis, D., Deller, M.C., Klasse, P.J., Burton, D.R., Sanders, R.W., Moore, J.P.*, et al.* (2013). Crystal structure of a soluble cleaved HIV-1 envelope trimer. Science *342*, 1477-1483.

Kabsch, W. (2010). Xds. Acta Crystallogr., Sect: D Biol. Crystallogr. *66*, 125-132.

Lander, G.C., Stagg, S.M., Voss, N.R., Cheng, A., Fellmann, D., Pulokas, J., Yoshioka, C., Irving, C., Mulder, A., Lau, P.W.*, et al.* (2009). Appion: an integrated, database-driven pipeline to facilitate EM image processing. J. Struct. Biol. *166*, 95-102.

Lee, J.H., de Val, N., Lyumkis, D., and Ward, A.B. (2015). Model building and refinement of a natively glycosylated HIV-1 Env protein by high-resolution cryoelectron microscopy. Structure *23*, 1943-1951.

McCoy, A.J., Grosse-Kunstleve, R.W., Adams, P.D., Winn, M.D., Storoni, L.C., and Read, R.J. (2007). Phaser crystallographic software. J. Appl. Crystallogr. *40*, 658-674.

McLellan, J.S., Pancera, M., Carrico, C., Gorman, J., Julien, J.P., Khayat, R., Louder, R., Pejchal, R., Sastry, M., Dai, K.*, et al.* (2011). Structure of HIV-1 gp120 V1/V2 domain with broadly neutralizing antibody PG9. Nature *480*, 336-343.

Ogura, T., Iwasaki, K., and Sato, C. (2003). Topology representing network enables highly accurate classification of protein images taken by cryo electron-microscope without masking. J. Struct. Biol. *143*, 185-200.

Otwinowski, Z., and Minor, W. (1997). Processing of X-ray diffraction data collected in oscillation mode. Methods Enzymol. *276A*, 307-326.

Pedregosa, F., Varoquaux, G., Gramfort, A., Michel, V., Thirion, B., Grisel, O., Blondel, M., Prettenhofer, P., Weiss, R., Dubourg, V.*, et al.* (2011). Scikit-learn: machine learning in Python. J. Mach. Learn. Res. *12*, 2825-2830.

Pettersen, E.F., Goddard, T.D., Huang, C.C., Couch, G.S., Greenblatt, D.M., Meng, E.C., and Ferrin, T.E. (2004). UCSF Chimera--a visualization system for exploratory research and analysis. J. Comput. Chem. *25*, 1605-1612.

Pugach, P., Ozorowski, G., Cupo, A., Ringe, R., Yasmeen, A., de Val, N., Derking, R., Kim, H.J., Korzun, J., Golabek, M.*, et al.* (2015). A native-like SOSIP.664 trimer based on an HIV-1 subtype B Env gene. J. Virol. *89*, 3380-3395.

Ramey, V.H., Wang, H.W., and Nogales, E. (2009). Ab initio reconstruction of helical samples with heterogeneity, disorder and coexisting symmetries. J. Struct. Biol. *167*, 97-105.

Sok, D., van Gils, M.J., Pauthner, M., Julien, J.P., Saye-Francisco, K.L., Hsueh, J., Briney, B., Lee, J.H., Le, K.M., Lee, P.S.*, et al.* (2014). Recombinant HIV envelope trimer selects for quaternary-dependent antibodies targeting the trimer apex. Proc. Natl. Acad. Sci. USA *111*, 17624-17629.

Suloway, C., Pulokas, J., Fellmann, D., Cheng, A., Guerra, F., Quispe, J., Stagg, S., Potter, C.S., and Carragher, B. (2005). Automated molecular microscopy: the new Leginon system. J. Struct. Biol. *151*, 41-60.

Walker, L.M., Huber, M., Doores, K.J., Falkowska, E., Pejchal, R., Julien, J.P., Wang, S.K., Ramos, A., Chan-Hui, P.Y., Moyle, M.*, et al.* (2011). Broad neutralization coverage of HIV by multiple highly potent antibodies. Nature *477*, 466-470.

Webb, B., and Sali, A. (2016). Comparative protein structure modeling using MODELLER. Curr. Protoc. Bioinformatics *54*, 5 6 1-5 6 37.

Yasmeen, A., Ringe, R., Derking, R., Cupo, A., Julien, J.P., Burton, D.R., Ward, A.B., Wilson, I.A., Sanders, R.W., Moore, J.P., and Klasse, P.J. (2014). Differential binding of neutralizing and non-neutralizing antibodies to native-like soluble HIV-1 Env trimers, uncleaved Env proteins, and monomeric subunits. Retrovirology *11*, 41.
