# Supplementary material for: A Broadly Neutralizing Antibody Targets the Dynamic HIV Envelope Trimer Apex via a Long, Rigidified, and Anionic β-Hairpin Structure
Source: Immunity. 2017 Apr 18;46(4):690–702. doi: 10.1016/j.immuni.2017.03.017 (PMC5400778; doi:10.1016/j.immuni.2017.03.017)
Supplement: Document S2. Article plus Supplemental Information [file mmc2.pdf]

Immunity

Article

A Broadly Neutralizing Antibody Targets the Dynamic HIV Envelope Trimer Apex via a Long, Rigidified, and Anionic β-Hairpin Structure

Highlights

- Apex binding antibody PGT145 engages all three gp120 protomers simultaneously
- Epitope recognition is chemical-feature specific
- PGT145-class antibodies exhibit structural features that reflect bovine antibodies
- PGT145-class antibody maturation is dependent on structural stabilization of HCDR3

Authors

Jeong Hyun Lee, Raiees Andrabi, Ching-Yao Su, ..., Ian A. Wilson, Dennis R. Burton, Andrew B. Ward

Correspondence

burton@scripps.edu (D.R.B.), andrew@scripps.edu (A.B.W.)

In Brief

Broadly neutralizing antibodies of the PGT145-family target the HIV-1 Env trimer apex via a long β-hairpin HCDR3, but the molecular basis of recognition is unknown. Using cryoEM, Lee et al. (2017) reveal how PGT145 binds its quaternary epitope and the importance of HCDR2 evolution despite its lack of contacts with Env.

Accession Numbers

5UXQ
5UY3
5V8M
5V8L

Lee et al., 2017, Immunity *46*, 690–702
CrossMark
April 18, 2017 © 2017 The Author(s). Published by Elsevier Inc.
http://dx.doi.org/10.1016/j.immuni.2017.03.017

CellPress

Immunity
Article

A Broadly Neutralizing Antibody Targets the Dynamic HIV Envelope Trimer Apex via a Long, Rigidified, and Anionic β-Hairpin Structure

Jeong Hyun Lee,[1,2,9] Raiees Andrabi,[2,3,9] Ching-Yao Su,[3] Anila Yasmeen,[4] Jean-Philippe Julien,[1,2,5] Leopold Kong,[1] Nicholas C. Wu,[1] Ryan McBride,[6] Devin Sok,[2,3] Matthias Pauthner,[2,3] Christopher A. Cottrell,[1,2] Travis Nieusma,[1] Claudia Blattner,[1,2] James C. Paulson,[6] Per Johan Klasse,[4] Ian A. Wilson,[1,2,7] Dennis R. Burton,[2,3,8,*] and Andrew B. Ward[1,2,10,*]

[1]Department of Integrative Structural and Computational Biology, The Scripps Research Institute, La Jolla, CA 92037, USA
[2]Center for HIV/AIDS Vaccine Immunology and Immunogen Discovery, International AIDS Vaccine Initiative Neutralizing Antibody Center and Collaboration for AIDS Vaccine Discovery, The Scripps Research Institute, La Jolla, CA 92037, USA
[3]Department of Immunology and Microbiology, The Scripps Research Institute, La Jolla, CA 92037, USA
[4]Weill Medical College of Cornell University, New York, New York 10065, USA
[5]Program in Molecular Structure and Function, Hospital for Sick Children Research Institute, and Departments of Biochemistry and Immunology, University of Toronto, Toronto, Ontario M5S 1A8, Canada
[6]Department of Cell and Molecular Biology and Chemical Physiology, The Scripps Research Institute, La Jolla, California 92037, USA
[7]Skaggs Institute for Chemical Biology, The Scripps Research Institute, La Jolla, CA 92037, USA
[8]Ragon Institute of MGH, MIT and Harvard, Boston, MA 02139, USA
[9]Co-first author
[10]Lead contact
*Correspondence: burton@scripps.edu (D.R.B.), andrew@scripps.edu (A.B.W.)
http://dx.doi.org/10.1016/j.immuni.2017.03.017

SUMMARY

Broadly neutralizing antibodies (bnAbs) to HIV delineate vaccine targets and are prophylactic and therapeutic agents. Some of the most potent bnAbs target a quaternary epitope at the apex of the surface HIV envelope (Env) trimer. Using cryo-electron microscopy, we solved the atomic structure of an apex bnAb, PGT145, in complex with Env. We showed that the long anionic HCDR3 of PGT145 penetrated between glycans at the trimer 3-fold axis, to contact peptide residues from all three Env protomers, and thus explains its highly trimer-specific nature. Somatic hypermutation in the other CDRs of PGT145 were crucially involved in stabilizing the structure of the HCDR3, similar to bovine antibodies, to aid in recognition of a cluster of conserved basic residues hypothesized to facilitate trimer disassembly during viral entry. Overall, the findings exemplify the creative solutions that the human immune system can evolve to recognize a conserved motif buried under a canopy of glycans.

INTRODUCTION

Numerous antibodies that target and neutralize a broad range of different human immunodeficiency virus (HIV) isolates have been found in chronically infected HIV donors. Some of these bnAbs inhibit HIV Env with remarkable breadth and potency by recognizing conserved “supersites” of vulnerability (Burton and Hangartner, 2016). One of these epitope clusters is located at the trimer apex, consisting of the variable loops 1 and 2 (V1/V2) that hold together the gp120 subunits of the trimer through inter-protomer interactions (Doria-Rose et al., 2014; McLellan et al., 2011; Sok et al., 2014; Walker et al., 2009).

True to its name, the V1/V2 region varies greatly in sequence and length. All HIV isolates nevertheless retain two notable features in this region. The V2 contains N-linked glycosylation sites at positions N160 and N156 (or the less common compensatory position N173), and a cluster of positively charged amino acids around the trimer 3-fold symmetry axis (Andrabi et al., 2015). In this manner, the trimer apex forms an immunogenic, structurally conserved motif consisting of an electropositive hole surrounded by N-linked glycans. Examples of patient-derived bnAbs that belong to this class include PG9, PG16, CH01-CH04, the CAP256-VRC26 lineage, PGT141-145, and PGDM1400-1412 (Doria-Rose et al., 2014; McLellan et al., 2011; Sok et al., 2014; Walker et al., 2011; Walker et al., 2009). PGDM1400 (83% breadth, 0.003 μg/mL median IC_{50}) and CAP256-VRC26.25 (57% breadth, 0.001 μg/mL median IC_{50}), in particular, are remarkably potent (Doria-Rose et al., 2015; Sok et al., 2014).

Partial descriptions of paratope-epitope interactions have been obtained using epitope scaffolds with PG9 (McLellan et al., 2011), PG16 (Pancera et al., 2013), and the CH01-CH04 apex bnAbs (Gorman et al., 2016). Hybrid-modeling approaches employing low-resolution negative-stain EM (Julien et al., 2013b) and X-ray structures of scaffolds indicate these bnAbs bind at or near the trimer 3-fold axis with a binding stoichiometry of one antigen-binding fragment (Fab) per trimer. This binding mode results in a symmetry mismatch, unique to this class of antibodies, and glycan heterogeneity makes them difficult targets for structural studies (Sok et al., 2014). All characterized apex bnAbs, except for some CAP256-VRC26 lineage antibodies

This is an open access article under the CC BY license (http://creativecommons.org/licenses/by/4.0/).

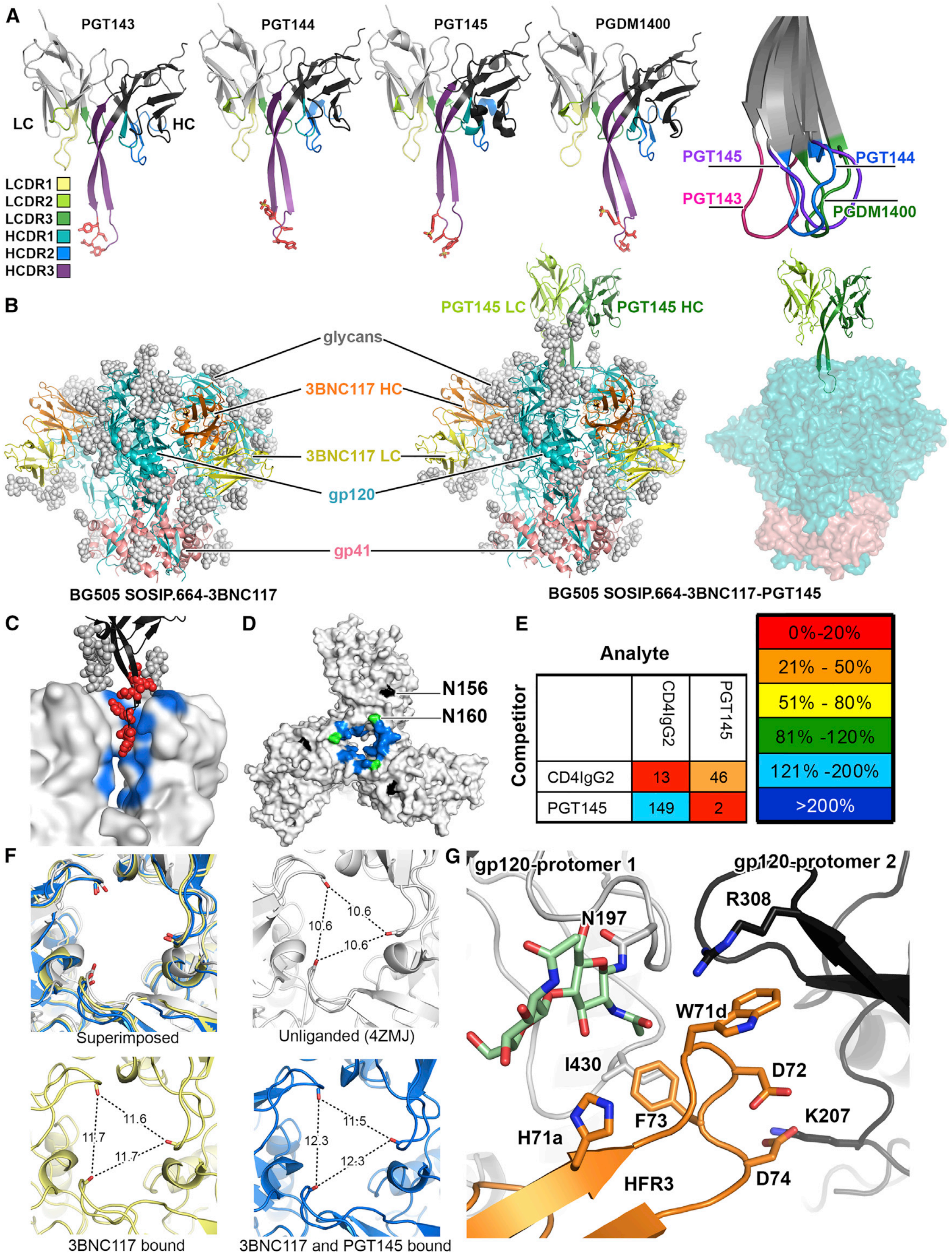

Figure 1. BG505 SOSIP Trimer in Complex with PGT145 Reveals HCDR3 Guided Epitope Recognition at the Center of the Trimer Apex

(A) X-ray structures of PGT145-family Fabs. Red sticks: acidic residues and observed or predicted sulfated tyrosines in the β-turn. The CDRs are colored according to the key shown on the far left. HC and LC are shown in dark and light gray respectively. Far right: superimposition of all HCDR3s shows different β-loop conformations.

(B) Models of 3BNC117 (left) and 3BNC117+PGT145 (middle) bound trimers. Glycans are shown as white spheres. PGT145 binds at a distance from the trimer as shown in the trimer model with glycans removed (right).

(C) PGT145 HCDR3 (black) inserts into a pit created by N160 glycans (gray spheres) and gp120 (gray surface), where its electronegative side chains (red) contact the electropositive epitope (blue).

(legend continued on next page)

(Doria-Rose et al., 2014), depend on glycans at N160 and N156/N173, and often fail to bind viruses produced in the presence of α-mannosidase-I inhibitor kifunensine (Kif) that results in homogeneous oligomannose glycans with 8-9 mannose (Man) residues (Andrabi et al., 2015; Sok et al., 2014). The structural basis of Env recognition for the PGT145-class of antibodies is highly sought after because its quaternary specificity is now widely exploited to detect and isolate properly formed Env trimers (de Taeye et al., 2015; Pugach et al., 2015), including under GMP conditions for human vaccine trials. Using cryo-electron microscopy (cryoEM), we determined the structure of PGT145 Fab in complex with the soluble, recombinant Env trimer, BG505 SOSIP.664 (Sanders et al., 2013) to elucidate key molecular interactions at the Env apex. Our structural and biochemical analyses revealed that PGT145-class bnAbs utilize their CDR loops, especially HCDR2 to stabilize a long anti-parallel β-hairpin HCDR3. This structural rigidity allows the antibody to penetrate through the tightly packed N160 glycan shield network, to recognize the electropositive sink generated by the protein elements at the core of the trimer apex. Therefore, despite nearly all epitope contacts coming from the HCDR3, additional maturation of the remaining CDR loops influences the HCDR3 and is crucial for generating a potent PGT145-like antibody.

RESULTS

PGT145 Recognizes a Quaternary Epitope at the Apical 3-fold Symmetry Axis of the Env Trimer

Apex bnAbs discovered so far can be grouped according to their heavy chain (HC) complementarity determining region (CDR) 3 topology: (1) PG9-like with—or predicted to have—a hammerhead motif (Doria-Rose et al., 2015; Doria-Rose et al., 2014; Gorman et al., 2016; McLellan et al., 2011; Pancera et al., 2013); or (2) PGT145-like with a long, anti-parallel β-hairpin (McLellan et al., 2011; Sok et al., 2014). Here, we solved X-ray structures of unliganded PGT143 and PGT144 Fabs, and they too exhibit the β-hairpin HCDR3 motif as expected (Figure 1A, Table 1, Figure S1). The elongated HCDR3 conformation of this PGT145-class bnAbs results in a paratope that projects a long distance away from the surface of the Fab and enables epitope recognition at the C3 axis of the trimer apex via a long-range interaction (Figures 1B and 1C, S2A) (Sok et al., 2014). To define the molecular interactions of an apex antibody, we generated the structures of BG505 SOSIP.664-3BNC117-PGT145 and BG505 SOSIP.664-3BNC117 by single particle cryoEM at global resolutions of ~4.3 Å and ~4.4 Å, respectively (Figures 1B, S2B–S2G). The PGT145 HCDR3 inserted perpendicularly to the V1/V2 β sheets between the triad of N160 glycans into a pocket formed at the trimer apex, to simultaneously engage all three gp120s (Figures 1B–1D). This quaternary epitope is only present in the correctly folded pre-fusion Env conformation (Sok et al., 2014).

The trimer-specific PGT145 is one of the few antibodies with which we could previously extract cleaved wild-type JR-FL E168K EnvΔCT trimers from cell membranes (Figure S2A) (Blattner et al., 2014). PGT145 also increases the thermal stability of BG505 SOSIP.664 upon binding (Cheng et al., 2015) and is predicted to neutralize HIV by stabilizing the prefusion conformation of the Env trimer. However, surface plasmon resonance (SPR) showed improved binding of the Env-receptor mimic CD4-IgG2 (Liu et al., 2008), when PGT145 Fab had pre-bound the trimer (Figures 1E, S3A). A subtle opening of the trimer apex induced by PGT145 HCDR3 (Figure 1F) might improve access to the CD4-binding site, albeit shown only in vitro with soluble components. In vivo, PGT145 IgG binding at the Env apex would however provide a steric block to CD4 binding on the host cell membrane.

In the BG505-3BNC117 complex, we also observed a subtle opening of the trimer apex (Kwon et al., 2015) (Figure 1F) that appeared to be mediated by HC framework region 3 (HFR3) of 3BNC117. 3BNC117 and its clonal relative, 3BNC60, have a ^{71d}WDFD^{74} insertion in HFR3, which is critical for neutralizing activity (Klein et al., 2013). In 3BNC117, the HFR3 interacts with the N-acetylglucosamine (GlcNAc) core of the N197 glycan with H71a, and residue W71d interacted with V3 R308 of the adjacent gp120 (Figure 1G). Env residue 308 is typically Arg or His (Arg: 32%, His: 39%), both of which provide favorable interactions with tryptophan. Thus, while PGT145 recognizes the closed conformation of the Env trimer, the trimer apex is very subtly open in the PGT145 bound conformation. Induced allosteric effects, such as 3BNC117 binding at the CD4 binding site, that lead to an increase in spacing between the N160 glycan triad, likely make the apical binding site more accessible to PGT145.

PGT145 Recognizes Oligomannose Glycans

The glycoforms to which apex antibodies bind have been a matter of debate. Studies have shown that apex antibodies such as PG16 bind sialylated hybrid glycans with much higher affinity than oligomannose glycans (Andrabi et al., 2015; Pancera et al., 2013). Similarly, PG9 showed increased binding affinity, neutralization potency, and maximum percentage neutralization (MPN) in the presence of complex/hybrid glycans (Figure 2A). PGT145-family antibodies have also been shown to preferentially bind to wild-type viruses and trimers compared to Kif treated Envs (Sok et al., 2014). However, SPR analysis of PGT145 Fab binding to BG505 SOSIP.664 trimers demonstrated that PGT145 has the highest affinity for glucose N-acetyltransferase 1 (GnT1)-deficient 293S-produced trimers that only have oligomannose Man_{5-9} glycans, although the larger Man_{8-9} glycans in the +Kif condition are somewhat inhibitory (Figures 2B, S3B and S3C). Further, a 31-pseudovirus neutralization panel showed a ~3.5-fold improved median IC_{50} of PGT145 in 293S compared to wild-type glycan producing 293T cell produced pseudoviruses (Figure 2A), suggesting that, while PGT145 can

(D) Env residues within a 4 Å radius of the PGT145 CDR loops (blue).

(E) SPR competition assay illustrating the non-reciprocal relationship between CD4 and PGT145 binding. PGT145 enhances CD4 binding while CD4 induces a conformational change that destroys the PGT145 epitope.

(F) 3-fold apex regions of 3BNC117-bound (yellow), 3BNC117^{+}PGT145-bound (blue) and unliganded (gray) trimer. Inter-V2 distances (Å) are measured between the Cα residues of R166 (dashed lines).

(G) 3BNC117 HFR3 interaction with the proximal (gray) and adjacent (black) gp120 protomers.

Table 1. X-ray Data Collection and Refinement Statistics

	PGT143 Fab	PGT144 Fab
Beamline	APS 23ID-B	APS 23-ID-D
Wavelength, Å	1.033	1.0332
Space group	$P2_12_12_1$	P1
Unit cell a, b, c (Å)	70.9, 97.6, 126.2	57.6, 67.6, 69.6
α, β, γ (°)	90.00, 90.00, 90.00	82.01, 74.97, 81.28
Resolution (Å)	48.8 – 2.40 (2.44 – 2.40)[a]	40 – 2.9 (3.0 – 2.9)[a]
Completeness	95.1 (87.4)[a]	96.6 (96.9)[a]
Redundancy	3.5 (2.5)[a]	2.1 (2.1)[a]
No. total reflections	113,011	45,828
No. unique reflections	32,477	21,292
I/σ	9.6 (2.2)[a]	5.5 (1.9)[a]
R_{sym}	0.11 (0.49)[a, b]	0.16 (0.39)[a, b]
R_{pim}	0.06 (0.36)[a]	0.14 (0.35)[a]
$CC_{1/2}$	97.0 (90.0)[a]	94.8 (59.1)[a]
Refinement Statistics		
Resolution (Å)	48.8-2.4	40 – 2.9
No. reflections total/R_{free}	30,746/1,652	21,284/1,064
R_{cryst}/R_{free}	22.9[c]/ 26.7[d]	24.11[c]/28.2[d]
RMSD Bond Length (Å)	0.003	0.0025
RMSD Bond Angles (°)	0.900	0.620
Protein Atoms	6,913	6,856
Wilson B-value ($Å^2$)	46.6	38.6
Overall average B-value ($Å^2$)	38.6	42.5
Ramachandran		
Favored (%)	96.2	95.5
Allowed (%)	3.6	4.0
MolProbity all-atom clashscore	7.4[e]	8.7[e]
PDB ID	5UXQ	5UY3

[a]Numbers in parentheses refer to the highest resolution shell.
[b]$R_{sym} = \Sigma_{hkl}\Sigma_i \mid I_{hkl,i} - < I_{hkl}> \mid / \Sigma_{hkl}\Sigma_i I_{hkl,I}$, where $I_{hkl,i}$ is the scaled intensity of the ith measurement of relection h, k, l, $< I_{hkl}>$ is the average intensity for that reflection, and n is the redundancy.
[c]$R_{cryst} = \Sigma_{hkl} \mid F_o - F_c \mid / \Sigma_{hkl} \mid F_o \mid$ x 100
[d]R_{free} was calculated as for R_{cryst}, but on a test set comprising 5% of the data excluded from refinement.
[e]These values were calculated using MolProbity.

accommodate glycan heterogeneity, there is a preference for oligomannose forms.

Previously, glycan arrays failed to detect PGT145 binding across a wide range of glycans (Andrabi et al., 2015). Here, we employed a dendrimer format of glycan array slides (Wang et al., 2008) that creates a higher density of glycans. PGT145 and PGDM1400 bound the C-isomer of a Man_8 glycan (M8C) (Figures S3D–S3F), but not the Man_8 B-isomer (M8B) indicating that the terminal Man(α1-2) residue of the D3 arm may sterically clash with PGT145-family Fabs. These data suggested that not only the correct glycoform, but also close spacing of glycans is essential for PGT145 binding.

The PGT145 Epitope Consists of Glycans from Two Protomers and Peptide Contacts from all Three Protomers

In the cryoEM map, the primary interacting N160 glycan appeared as oligomannose, consistent with our glycan array analysis. None of the asymmetric N160 glycans revealed density corresponding to core fucosylation (Figures S4A and S4B), often found in complex glycans. The total buried surface area between PGT145 and the modeled N160 glycans was ~555 $Å^2$ (glycan 1: 384 $Å^2$, glycan 2: 131 $Å^2$, glycan 3: 40 $Å^2$). PGT145 most prominently interacted with a single N160 glycan ($N160_{glycan1}$), less extensively with a second N160 glycan ($N160_{glycan2}$), and likely not at all with the third N160 glycan ($N160_{glycan3}$) (Figures 3A–3D, S4A–S4C). The density resolved for $N160_{glycan1}$ is approximately the size of Man_6 (Figure S4A) and its core GlcNAc sugars bound the peptide backbone of the HCDR3 descending strand, while the D1 arm packed between HCDR2 and HCDR3, making polar contacts with highly conserved $H52a_{HCDR2}$ (Figure 3B). $Y100f_{HCDR3}$—a tyrosine known to be sulfated—orients R100a $_{HCDR3}$, and the sulfate might also interact with $N160_{glycan1}$ Asn, while the benzene ring face of Y100f $_{HCDR3}$ is packed against T162 (Figure 2B). Although the D2 and D3 arms of $N160_{glycan1}$ are not fully resolved, we infer from glycan array data that the terminal D3 arm would clash with the LC of PGT145 in its orientation in the structure (Figures 3A and S3E). Together, the data predicted that much of the glycan recognition is driven by interactions made with the GlcNAc stalk, with steric accommodation for a Man_6, Man_7, Man_8, or even a hybrid glycan at $N160_{glycan1}$. In contrast, a complex glycan with its bulkier Gal(β1-4)GlcNAc(β1-2/4/6) branching units would likely clash with PGT145. Thus, the mode of N160 glycan recognition differed from that of the PG9-like antibodies, in which the N160 glycan branches bury into a pocket formed by the hammerhead-shaped HCDR3 and the LCDRs (Figure S4D). The GlcNAc base of $N160_{glycan2}$ interacted with $Y100c_{HCDR3}$ and to some extent with LCDR1, although not well resolved in our structure (Figure 3C). LCDR1 is the most variable CDR segment among the PGT145-family variants (Figure S1B), suggesting that the PGT145-family antibodies might accommodate $N160_{glycan2}$ in different ways. $N160_{glycan3}$ projected away from the antibody likely because it is sterically inhibitory to PGT145 binding in its native conformation (Figures 3D, S4C). Consequently, the N160 glycan is both essential (on one, probably two protomers), as well as inhibitory (on the third protomer) to binding of PGT145, presenting a unique challenge to epitope recognition of an asymmetric antibody molecule at a symmetry axis.

In contrast to hammerhead HCDR3-type apex antibodies, which are dependent on the N156 glycan and bind complex glycoforms on glycan arrays (Andrabi et al., 2015; Gorman et al., 2016; McLellan et al., 2011; Pancera et al., 2013), none of the N156 glycans directly contacted PGT145 (Figures S4D and S4E). Ablation of the N156 glycosylation site by an N156K substitution abrogated neutralization by PGT145 (Figure 3E). We therefore looked for an indirect effect that could be ascribed to differential processing of N160 glycans in N156 glycan-lacking Env variants expressed in different cell-lines. SPR showed negligible binding of PGT145 Fab to all of the N156D substitution variants (Figure S3B), and inspection of the N156D mutant by negative stain EM revealed that this mutation enriched for non-native-like

A

VIRUS	CLADE	PGT145 WT IC50 (μg/mL)	PGT145 WT MPN (%)	PGT145 293S IC50 (μg/mL)	PGT145 293S MPN (%)	PG9 WT IC50 (μg/mL)	PG9 WT MPN (%)	PG9 293S IC50 (μg/mL)	PG9 293S MPN (%)
Q23.17	A	0.096	64	> 10	0	0.002	100	0.024	90
191955_A11	A (T/F)	0.004	95	0.037	90	0.002	95	0.132	91
REJO4541.67	B	0.003	100	0.005	74	0.002	89	ND	ND
TRO.11	B	0.072	99	0.439	87	> 10	0	0.087	75
PVO.4	B	1.32	100	0.310	94	> 10	0	> 10	0
12_11_TC21_3257	B (T/F)	0.022	100	0.054	99	0.018	80	0.138	76
006_11_C3_1601	B (T/F)	> 10	0	> 10	0	0.082	82	0.005	79
ZM53M.PB12	C	0.117	71	0.094	88	0.014	98	0.072	89
CAP45.2.00.G3	C	0.002	96	0.013	95	0.003	99	0.001	84
HIV-16055-2.3	C	0.020	87	0.006	87	0.004	100	0.002	88
HIV-001428-2.42	C	0.004	88	0.001	90	0.002	100	0.004	92
CAP210.2.00.E8	C	> 10	0	0.293	81	0.047	95	0.008	96
1394C9G1(Rev-)	C (T/F)	0.006	92	0.001	97	0.006	97	0.001	91
Ce1176_A3	C (T/F)	> 10	0	> 10	0	0.003	99	0.123	89
e703010054_2A2	C (T/F)	> 10	0	> 10	0	0.003	100	0.003	100
231966.c02	D	1.52	68	0.065	53	0.011	50	0.007	54
X1632_S2_B10	G	0.092	71	> 10	0	0.008	84	0.043	83
CNE21	BC	0.010	90	0.005	75	0.007	91	0.044	80
CNE17	BC	0.145	90	0.010	76	0.039	99	0.201	94
CNE52	BC	0.045	99	0.060	87	0.007	99	0.016	84
CNE53	BC	0.085	95	0.011	77	0.006	89	> 10	0
89-F1_2_25	CD	0.608	68	0.091	44	0.023	81	0.004	61
3817.v2.c59	CD	> 10	0	> 10	0	0.001	98	0.010	87
3103.v3.c10	ACD	0.284	97	> 10	0	> 10	0	> 10	0
620345.c01	CRF01_AE	0.003	74	0.001	91	0.001	70	0.003	86
C3347.c11	CRF01_AE	0.052	93	> 10	0	0.006	97	> 10	0
C4118.c09	CRF01_AE	0.007	98	0.004	83	0.005	97	0.023	86
BJOX028000.10.3	CRF01_AE (T/F)	> 10	0	> 10	0	0.009	71	> 10	0
T250-4	CRF02_AG	0.023	87	> 10	0	0.001	99	> 10	0
T257-31	CRF02_AG	> 10	0	> 10	0	0.004	96	> 10	0
T278-50	CRF02_AG	> 10	0	> 10	0	0.049	80	0.011	89
% BREADTH		74%		61%		90%		74%	
MEDIAN IC50 (μg/mL)		0.045		0.013		0.006		0.011	
MEDIAN MPN (%)		87		75		95		84	

B

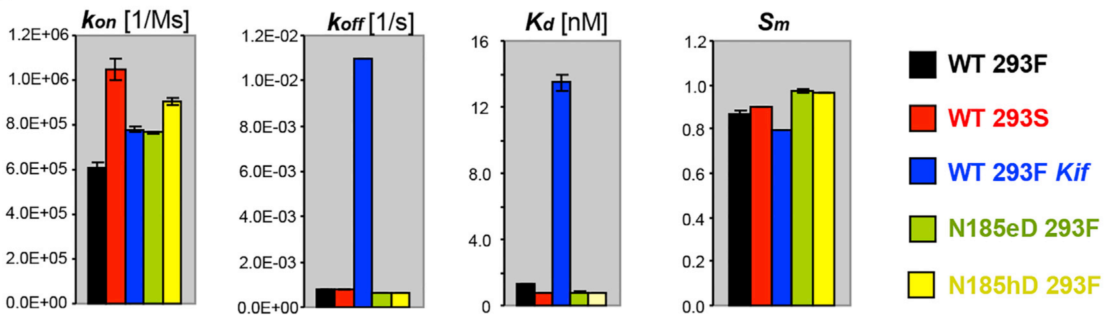

Figure 2. PGT145 Favorably Binds Oligomannose Glycans

(A) IC_{50} and maximum percentage neutralization (MPN) values for PGT145 and PG9 neutralization of pseudoviruses produced in 293T or 293S cells. ND: Not determined.

(B) A comparison of kinetic parameters derived from SPR analyses of PGT145 binding to BG505 SOSIP.664 trimers produced in trimers expressing different glycoforms and N-linked glycosylation site mutations. Also see Figures S3B and S3C.

trimers (Figure S4F). Thus, the reduced binding of PGT145 to N156 glycan knockouts most likely arose from disruption of the conformational epitope through the loss of the inter-V2-strand interaction between Y173 and N156 core GlcNAc. Residue 173 is a Tyr, His, or Phe in >86% of Envs, all of which are suitable for stabilizing the N156 glycan. Indirect glycan effects on the PGT145 epitope might also result from variability in the length and number of glycosylation sites on V1 and V2 loops, as such viral attributes have been noted to increase with disease progression and correlate with reduced neutralization sensitivity (Curlin et al., 2010; Sagar et al., 2006). The most variable segment of V2, which is distal to the 3-fold axis, is flexible and disordered in the majority of high-resolution SOSIP.664 structures. The density corresponding to the projection of the V2 loop in BG505, however, suggested that its glycans would be close to the bound PGT145 Fab, and the N-linked glycosylation sites at N185e and

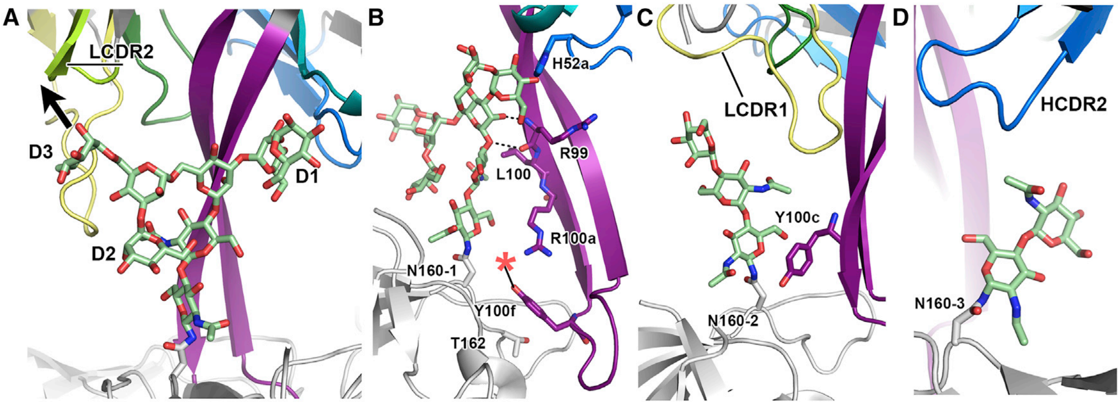

E

BG505.Env.C2	PGDM1400	PGDM1401	PGDM1404	PGDM1406	PGDM1409	PGT143	PGT145	PG9	CH01	CAP256.09	VRC01
WT	0.016	0.009	3.244	>10	0.601	0.028	0.033	0.032	0.579	0.044	0.164
K121A	0.059	5.679	>10	>10	>10	>10	>10	0.018	0.092	0.397	0.105
V127A	>10	>10	>10	>10	>10	>10	>10	0.418	>10	>10	0.252
N156K	2.348	>10	>10	>10	>10	>10	>10	1.441	>10	>10	0.159
N160K	>10	>10	>10	>10	>10	>10	>10	>10	>10	>10	0.162
N160A	>10	>10	>10	>10	>10	>10	>10	>10	>10	>10	0.037
M161A	0.040	0.149	>10	>10	>10	0.629	2.003	0.038	0.359	0.008	0.091
T162A	>10	>10	>10	>10	>10	>10	>10	>10	>10	>10	0.111
E164A	0.011	0.008	>10	>10	6.558	0.024	0.089	0.016	0.389	1.492	0.116
L165A	0.039	8.611	>10	>10	>10	4.320	5.539	0.017	0.172	>10	0.069
R166A	>10	>10	>10	>10	>10	>10	>10	0.024	0.433	>10	0.201
D167A	>10	>10	>10	>10	>10	>10	>10	0.016	0.162	>10	0.069
K168A	0.787	5.193	>10	>10	>10	4.402	9.110	0.587	2.469	0.192	0.095
K169A	1.237	0.114	>10	>10	>10	0.084	8.493	0.054	1.673	>10	0.155
I307A	0.015	0.007	>10	>10	>10	0.056	0.009	0.024	0.204	0.418	0.171
I420A	0.030	0.029	>10	>10	5.168	0.094	0.126	0.028	0.623	0.104	0.154
I423A	0.009	0.002	>10	>10	9.933	0.034	0.061	0.054	1.001	0.519	0.148

10	1	0.1	0.01	0.001	IC_{50} (μg/mL)

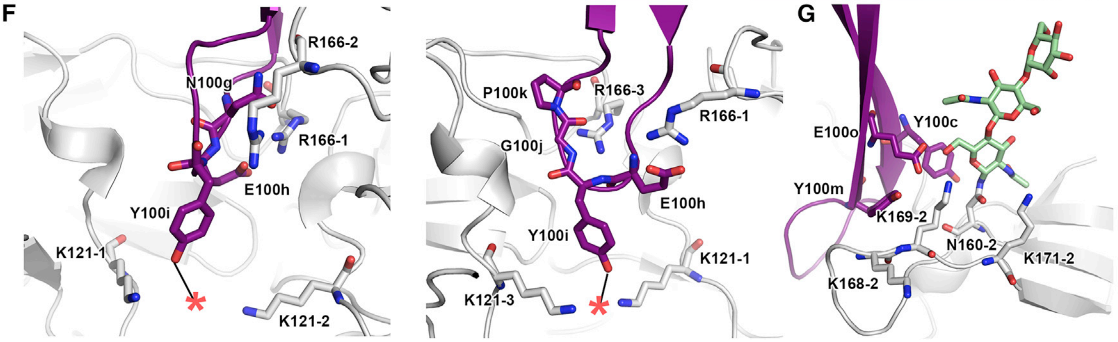

Figure 3. Simultaneous Engagement of Glycans and Protein Residues from Multiple gp120 Protomers Is Required for PGT145-Class bnAb Binding

(A) PGT145 interaction with $N160_{glycan1}$. Glycans are shown as green sticks, and the CDRs are colored as in Figure 1A. The arrow indicates the direction of the D3 branch of the glycan.

(B) $N160_{glycan1}$ interacts with the PGT145 HCDR3 peptide backbone. The red star indicates a sulfated tyrosine.

(C) PGT145 interaction with $N160_{glycan2}$.

(D) PGT145 interaction with $N160_{glycan3}$.

(E) Neutralization IC_{50} values of 293T cell-produced BG505 pseudovirus mutants by PGT145-family variants.

(F) Left and right panels: R166 and K121 from all three gp120 protomers (white) interact with the electronegative moieties in HCDR3. Red star indicates tyrosine sulfation observed in the X-ray structure of the Fab.

(G) Residues in HCDR3 along with $K169_{gp120}$ support interactions with the base of $N160_{glycan2}$.

N185h could restrict accessibility to the epitope (Figure S4G). Removal of the N185h glycan site in particular, led to a ~1.5-fold increase in the association constant of PGT145 Fab, and an increase in the stoichiometry (S_m) from 0.87 to 0.96 (Figures 2B, S3B and S3C), confirming that outer-V2 region glycans can partially shield the apex bnAb epitope.

Peptide contacts between PGT145 and Env (total buried surface area ~729 Å^2) are largely restricted to interactions between the tip of HCDR3 with the C-strand of V1/V2 and the C1 region near the base of V1 including K121 (Figure 3F). K121 from each protomer interacted with sulfated tyrosine Y100i, while R166 from each protomer interacted with various electronegative moieties in HCDR3 of PGT145 (Figure 3F). Finally, $Y100m_{HCDR3}$ stacked up against the hydrocarbon segment of K169, which probably helps engage the $N160_{glycan2}$ via a network of interactions along with E100o (Figure 3G).

To confirm our structural observations, we conducted alanine-scanning neutralization assays on BG505 pseudovirus mutants (Figures 3E, S5A). Neutralization was reduced when the N160 or N156 glycan sites were knocked out (N156K, N160K, N160A, T162A), or direct peptide contact residues were substituted (K121A, R166A). Point mutations in V1/V2 that caused BG505 SOSIP.664 trimers to adopt aberrant trimer forms (M161A, L165A, D167A) when observed by EM, selectively decreased neutralization potencies of PGT145-family bnAbs versus hammerhead-group, PG9 and CH01 (Figures 3E, S5). Neutralization by CAP256-VRC26.09 was affected mostly by the same Env mutations that affected the PGT145 class. Thus, despite CAP256-VRC26.09 having a HCDR3 structure that more closely resembles the hammerhead-class (Doria-Rose et al., 2014), it is dependent on proper quaternary apex formation like PGT145, and engages all three protomers upon binding. Overall, our results further classified PGT145 as a distinct class of apex bnAb, compared to PG9, and that it is an N160 glycan-dependent antibody that relies on simultaneous recognition of peptide contacts from the apex central residues of all three gp120 V1V2 loops, and is indirectly affected by the glycan at N156.

PGT145 Recognizes an Electropositive Sink at the Trimer Apex

Apex-bnAbs recognize the conserved electropositive V2, particularly strand C (Doria-Rose et al., 2012). We looked for additional residues near the central electropositive layer that could add to the overall local charge. K117, which is 97% conserved, is deeply buried under the trimer apex and is likely important for PGT145 recognition. Additionally, 84% of clade B Envs have an arginine or lysine at V3 position 315, instead of glutamine commonly found in other clades, effectively increasing the positive charge potential at the center of the trimer apex (Figure S6). Residue 315 is located between the electropositive layer created by strand C and K121, which is part of the PGT145 epitope, and thus the additional electropositive layer might interact favorably with the electronegative HCDR3 (Figure S6E). However, PGDM1400- and CAP256-VRC26-lineage bnAbs have lower clade B breadth (Doria-Rose et al., 2015; Sok et al., 2014). Therefore, this extra positive residue likely influences trimer apex dynamics through electrostatic repulsion, as often observed in clade B SOSIP trimers (de Taeye et al., 2015; Pugach et al., 2015). An increased tendency to “breathe” and open up at the apex would both alter the apex epitope but also enhance accessibility to the CD4bs, consistent with clade B Env infected-individuals preferentially generating CD4bs bnAb responses, but reduced apex responses (Rusert et al., 2016).

To determine whether there is any correlation between PGT145 IC_{50} and electropositive charges near the PGT145 epitope, we analyzed sequence variability at select positions in relation to neutralization using a linear regression model (Figure 4A). The goal of the model was to weigh the contribution of each residue to the escape phenotype defined by two different IC_{50} cut-off values, by considering the amino acid variation in each strain and their respective neutralization IC_{50} values. This weighting was reflected in the magnitude of the regression coefficient. The model predicts that the larger the magnitude of this coefficient, the more the residue influences resistance. In this analysis, residues that contact PGT145 and are important for neutralization, such as K121, R166, and T162, are also well conserved, having an exact match conservation score (relative to BG505) of ~70% or higher (Figure 4A). This analysis also revealed that K171 is important even though it does not directly contact PGT145. K171 seemed to interact with the base of the N160 glycan and/or restrict the movement range of N156 and N160 glycans to indirectly affect PGT145 binding (Figures 3G and 4B). A subtler effect on PGT145 neutralization was observed among hydrophobic residues involved in stabilization of the trimer apex (Figures 4A, 4C, and 4D). Particular V1/V2 residues have neutralization regression coefficients that were more prominent with an $IC_{50} > 1$ μg/mL cut-off, such as L125, I309, L175, and I326, which also tend to be more conserved. In binding, the anionic HCDR3 of PGT145 stabilizes the trimer by offsetting the net charge at the apex, especially when Env contains additional cationic residues. Mechanistically, Env requires CD4 triggering to transition into an open state leading to exposure of the co-receptor binding site. The proximal localization of positively charged residues results in repulsion between the gp120 protomers, contributing to meta-stability and priming Env for CD4 induced conformational changes. Too much repulsion between protomers would result in a constitutively open trimer susceptible to degradation and therefore render the virus defunct. Thus, we propose that a fine balance between tight hydrophobic packing and localization of electropositive charge is required for viral fitness, which PGT145 exploits for epitope targeting (Figure 4E).

PGT145 Potency Is Reliant on the Stability of Its HCDR3

While HCDR3 comprises the majority of the PGT145 paratope, the other CDR loops play important roles in neutralization. For example, reversion of HCDR2 in PGT143 and PGT145 to their predicted germline sequences abrogates neutralization (Andrabi et al., 2015) despite few direct contacts with Env (Figure 3). Structural examination revealed that HCDR3 makes extensive intra- and inter-CDR contacts in PGT145-family Fabs. Within the HCDR3, Y101 and D100b, and E100o and Y100m form electrostatic pairing interactions. W100p packs against R100a, which interacts with Y100f to orient the tyrosine side chain. Eliminating these pairwise stabilizing interactions reduced PGT145 neutralization (Figures 5A–5C, 6), indicating that structural stabilization of the long HCDR3 via tertiary and quaternary contacts is necessary for neutralizing activity.

We found a ^{52a}H-D/E-X-D/Q^{53} motif in HCDR2 that appeared to stabilize the HCDR3 stalk (Figures 5B, 6, S1A). PGT143-PGT145 and PGDM1400 all contained extensive electrostatic interactions between the HCDR2 and 3, such as the interaction between $R99_{HCDR3}$ with $D53_{HCDR2}$ (Q53 in PGDM1403-6) and $D100r_{HCDR3}$. The R99A paratope substitution had the second largest increase in PGT145 IC_{50} against all three pseudoviruses tested (Figure 5C). The $D100rA_{HCDR3}$ or $D53A_{HCDR2}$ substitutions also reduced potency, although not as severely as a single R99A substitution, which simultaneously ablates two electrostatic interactions. $K97_{HCDR3}$ formed electrostatic interactions with $E52b_{HCDR2}$ and $D33_{HCDR1}$, where $H52a_{HCDR2}$, a residue that contacted the $N160_{glycan1}$ also played a role in orienting the side chain of $E52b_{HCDR2}$. K97 is conserved across all PGT145-family

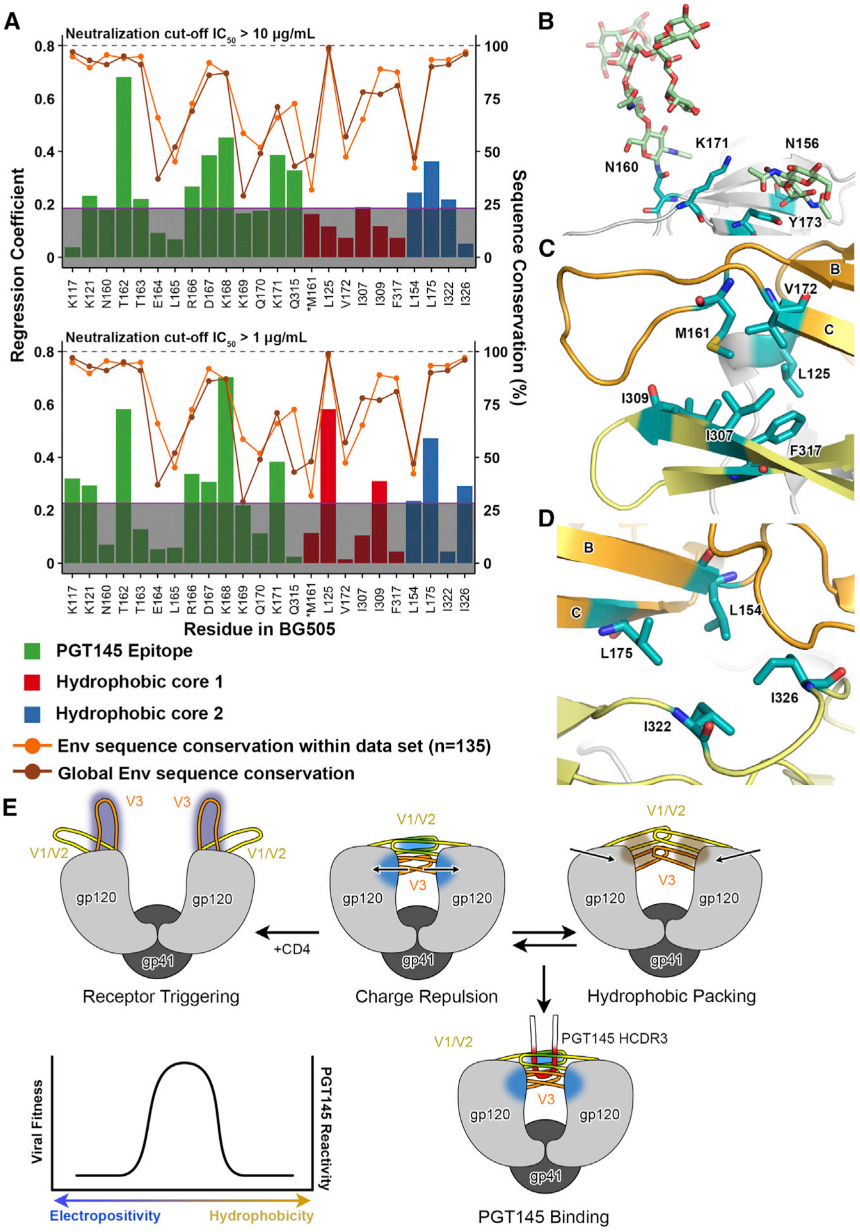

Figure 4. PGT145 IC_{50} Correlates with gp120 Residues Involved in Trimer Stability

(A) Logistic regression analysis was used to model neutralization escape based on amino acid sequence information. An IC_{50} of > 10 μg/mL (top) or > 1 μg/mL (bottom) was used to define escape. The relative conservation of the given residue is shown as a line graph. Residue-161 (asterisk) is also part of the direct PGT145 epitope.

(B) Y173 and K171 interact with N156 and N160 to stabilize V1/V2 and restrict glycan conformations.

(C) Hydrophobic packing interactions in hydrophobic core 1.

(D) Stabilizing interactions in hydrophobic core 2.

(E) Env is a meta-stable complex that transitions from a closed (top center and right) to an open unliganded trimer (top left) configuration. CD4 triggers a transition to the CD4-bound configuration (left) with an exposed co-receptor binding site (purple patch). The electronegative paratope of PGT145 (red patch, lower right) recognizes an electropositive pocket (blue), which is only present on a stable trimer. Both viral fitness and PGT145 reactivity require balance between electropositivity (blue) and hydrophobicity (tan) at the trimer apex (bottom left graph).

mAbs except for PGDM1411, a weak neutralizer that has K98 in place of K97 and R99, thereby incompletely substituting for two basic residues. Further, introduction of the 4- (^{52a}H-E-G-D^{53}) or 6-residue HCDR2 (^{52}S-H-E-G-D-K^{54}) stabilization motif back into inferred-germline (iGL; germine reversion with the exception of HCDR3) PGT145 partially rescued the neutralizing activity of PGT145 iGL variants (Figures 5D and 5E). LCDR1 exhibited the greatest variability in CDR length and sequence (Figure S1B). Nevertheless, a semi-conserved $Y32_{LCDR1}$ (9 of 18 MAbs) when mutated to alanine in PGT145 resulted in a large drop in neutralization (Figure 5C). In our four Fab structures, $Y32_{LCDR1}$ hydrogen bonded with the π-nitrogen of conserved $H98_{HCDR3}$ (Figures 5B and 5C). The remaining nine PGDM14XX MAbs had an $F32_{LCDR1}$-$F98_{HCDR3}$ interaction pair instead that plausibly stabilizes the HCDR3 by π-stacking.

Based on the SHM patterns, B cell affinity selection during the Ab maturation process led to four distinct PGT145 Ab sub-lineages (Figures S1A and S1B), reminiscent of a fork, which contains a "shaft" consisting of mutations that accumulated early to give rise to the most recent common ancestor (MRCA) Ab. These mutations are shared across all the Ab lineage members, and the four "prongs" represent the sub-lineages. Conservation of the ^{52a}H-D/E-X-D/Q^{53} motif suggests that certain HCDR2 mutations arose in the MRCA Ab and were critical for stabilizing a given HCDR3, then further diversified as the Ab sub-lineages diverged. Most of the prong mutations occurred in the C-term half of HCDR2, HCDR3, and LCDR1, likely due to strong viral selection pressure. The insertions in LCDR1 likely correspond to different $N160_{glycan2}$ recognition strategies.

The overall matured HCDR3 β-motif in the PGT145 family is similar to bovine antibodies, which form an anti-parallel β sheet "stalk" (Wang et al., 2013) that support a globular domain at the turn of the β sheet, thought to recognize antigen. In humans, a cross-reactive influenza antibody called C05 uses a HCDR3 β-hairpin to bind the hemagglutinin receptor binding site and was shown to contain potential HCDR3 stabilizing mutations in HCDR1 and HCDR2 (Ekiert et al., 2012); although its HCDR3 reflects the hammerhead shape of PG9, rather than the long β sheet stalks in bovine antibodies and PGT145. Therefore the structural and sequence analysis reveals that the evolution of these types of antibodies is driven by somatic hypermutation

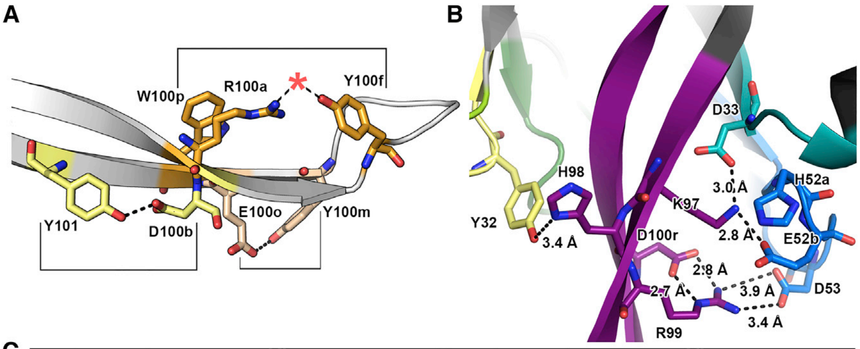

C

	Antibody	Virus		
	PGT145	WITO4160.33	CRF02_AG_250	BG505.Env.C2
	WT	0.003	0.005	0.010
HCDR3	G95A	0.003	0.005	0.024
	S96A	0.001	0.002	0.012
	K97A	0.192	0.145	4.088
	H98A	0.004	>10	8.159
	R99A	0.032	>10	>10
	L100A	0.004	>10	>10
	R100aA	0.002	0.001	0.017
	D100bA	0.002	2.187	>10
	Y100cA	0.003	>10	>10
	F100dA	0.003	0.787	2.380
	L100eA	0.001	0.865	9.991
	Y100fA	0.002	0.011	0.660
	N100gA	0.001	0.005	0.059
	E100hA	0.000	0.522	>10
	Y100iA	NA	NA	NA
	G100jA	0.002	0.006	0.096
	P100kA	0.001	0.158	0.356
	N100lA	0.001	0.002	0.004
	Y100mA	0.002	>10	>10
	E100nA	0.001	0.032	0.026
	E100oA	0.002	>10	>10
	W100pA	0.002	0.573	6.820
	G100qA	0.003	0.301	6.884
	D100rA	0.006	8.360	>10
	Y101A	0.007	>10	>10
	L102A	0.007	0.056	0.352
	A103G	0.004	0.014	0.179
	T104A	0.002	0.007	0.063
	L105A	0.003	0.250	0.510
	D106A	0.002	0.013	0.039
	V107A	0.002	0.001	0.025

	Antibody	Virus		
	PGT145	WITO4160.33	CRF02_AG_250	BG505.Env.C2
	WT	0.003	0.005	0.010
HCDR2	S52A	0.001	0.014	0.041
	H52aA	0.003	1.612	>10
	E52bA	0.012	>10	>10
	G52cA	0.002	0.003	0.016
	D53A	0.003	>10	>10
	K54A	0.002	0.003	0.015
LCDR1	L27bA	0.003	0.003	0.014
	Q27cA	0.003	0.012	0.037
	H27dA	0.004	0.109	0.240
	S27eA	0.001	0.003	0.002
	T28A	0.002	0.007	0.022
	G29A	0.002	0.003	0.029
	A30A	0.002	0.007	0.438
	N31A	0.002	0.002	0.019
	Y32A	0.449	>10	>10
Control	PGT128	1.693	0.005	0.004
	PGT151	0.006	0.002	0.002
	VRC01	0.111	>10	0.025

IC_{50} neutralization titers (μg/mL)
0.001
0.01
0.1
1
10
>10

D

```
                                      HCDR1 (30-35)          HCDR2 (50-63)
                                                             52                             82
                                                             abc                            abc
IGHV1-8-01  QVQLVQSGAEVKKPGASVKVSCKASGYTFTSYDINWVRQATGQGLEWMGWMNPNSGNTGYAQKFQGRVTMTRNTSISTAYMELSSLRSEDTAVYYCAR
PGT145_WT   ...............S..........NS.SNH.VH................SHEGDK..L.........I..DSGA..V....RG.TAD...I...LT
HC_iGL      ..................................................................................................
HCDR1_iGL   ...............S.................VH................SHEGDK..L.........I..DSGA..V....RG.TAD...I...LT
HCDR2_iGL   ...............S..........NS.SNH.VH......................L.........I..DSGA..V....RG.TAD...I...LT
HC_iGL+4    ....................................................HEGD..........................................
HC_iGL+6    ...................................................SHEGDK.........................................
```

E

Virus	Subtype	WT	LC iGL	HC iGL	HCDR1 iGL	HCDR2 iGL	HC iGL + 4	HC iGL + 6
WITO4160_33	B	<0.003	>10	1.063	<0.003	0.223	0.006	<0.003
REJO4541_67	B	<0.003	>10	>10	<0.003	>10	0.069	0.021
1012_11_TC21_3257	B	0.020	>10	>10	0.011	>10	0.163	0.087
CRF_250	AG	<0.003	>10	>10	0.006	>10	9.740	0.243
CNE21	BC	0.005	>10	>10	0.011	>10	>10	1.054
16055_2_3	C	0.015	>10	>10	0.019	>10	>10	3.062
CNE53	C	0.097	>10	>10	0.113	>10	>10	4.595
C1080_C3	AE	0.038	>10	>10	0.015	>10	>10	7.512
BG505.Env.C2	A	0.012	>10	>10	0.009	>10	>10	8.427
X1632_S2_B10	G	0.043	>10	>10	0.049	>10	>10	>10
3103_V3_C10	ACD	0.044	>10	>10	0.014	>10	>10	>10
89_F1_2_25	CD	1.000	>10	>10	0.825	>10	>10	>10
T257_31	AG	>10	>10	>10	>10	>10	>10	>10

IC_{50} (μg/mL)	Color scale	10	1	0.1	0.01	0.001

Figure 5. The Neutralization Potency of PGT145 Is Dependent on Stabilization of the HCDR3

(A) Interaction pairs within the HCDR3 are indicated by matching shades of color and brackets. The red star indicates the sulfated tyrosine found in the X-ray structure.

(B) The PGT145 HCDR3 forms an electrostatic interaction network with side chains of surrounding CDRs (colored as in Figure 1A). The Fab shown is from the cryoEM model.

(C) Neutralization of three different 293T-cell produced pseudoviruses by PGT145 Ala-substitution variants.

(D) Definition of iGL variants tested in (E).

(E) Neutralization of pseudoviruses by PGT145 iGL variants defined in (D).

in which substitutions stabilize an extended HCDR, thereby reducing the entropy for binding antigen.

DISCUSSION

HIV Env is remarkable in its ability to substantially alter its sequence and accommodate a high density of surface glycans and yet still fold and retain its conserved receptor binding and fusion functions. Here we described the structural basis of recognition and neutralization by a class of trimer apex-targeting bnAbs that exploit the intrinsic conservation in the trimer apex that enables Env to undergo receptor-induced conformational changes to attain its fusion active configuration. In the trimer ground state, the V1/V2 regions of the three protomers interact at the trimer apex to hold the gp120 subunits together and mask the co-receptor binding site. These interactions, however, cannot be overly stabilizing, as the V1/V2 region must remodel to enable the V3 base to bind the negatively charged N terminus of the CCR5/CXCR4 co-receptor for viral fusion to proceed (Liu et al., 2008; Rizzuto et al., 1998; Wu et al., 1996). We hypothesize that Env achieves this meta-stable balance by enriching the apex in basic amino acids, a subset of which are also important for co-receptor engagement. The PGT145-family bnAbs then exploit this cationic apex through extended, unusually stabilized HCDR3 loops that contain acidic residues and sulfated tyrosines distributed along the length of the β-hairpin loop, with most charge localized to the apical tip. The HCDR3 stalk is structurally conserved across the PGT145 family yet primarily interacts with a single glycan at N160. The stalk, stabilized by tertiary and quaternary interactions with the other CDR loops, serves as a platform to insert the somewhat variable tip of the β-hairpin into the glycan-shielded trimer apex. The 3-fold axis at the trimer apex comprises a large deep pocket that is permissive to a variety of negatively charged residues at the apical tip of the β-hairpin. This type of Env recognition is unique to this class of antibodies as most bnAb families, such as the VRC01-class (Scharf et al., 2016; Yacoob et al., 2016), PGT121-124 family (Steichen et al., 2016), or even PG9/PG16 (Andrabi et al., 2015), retain a few

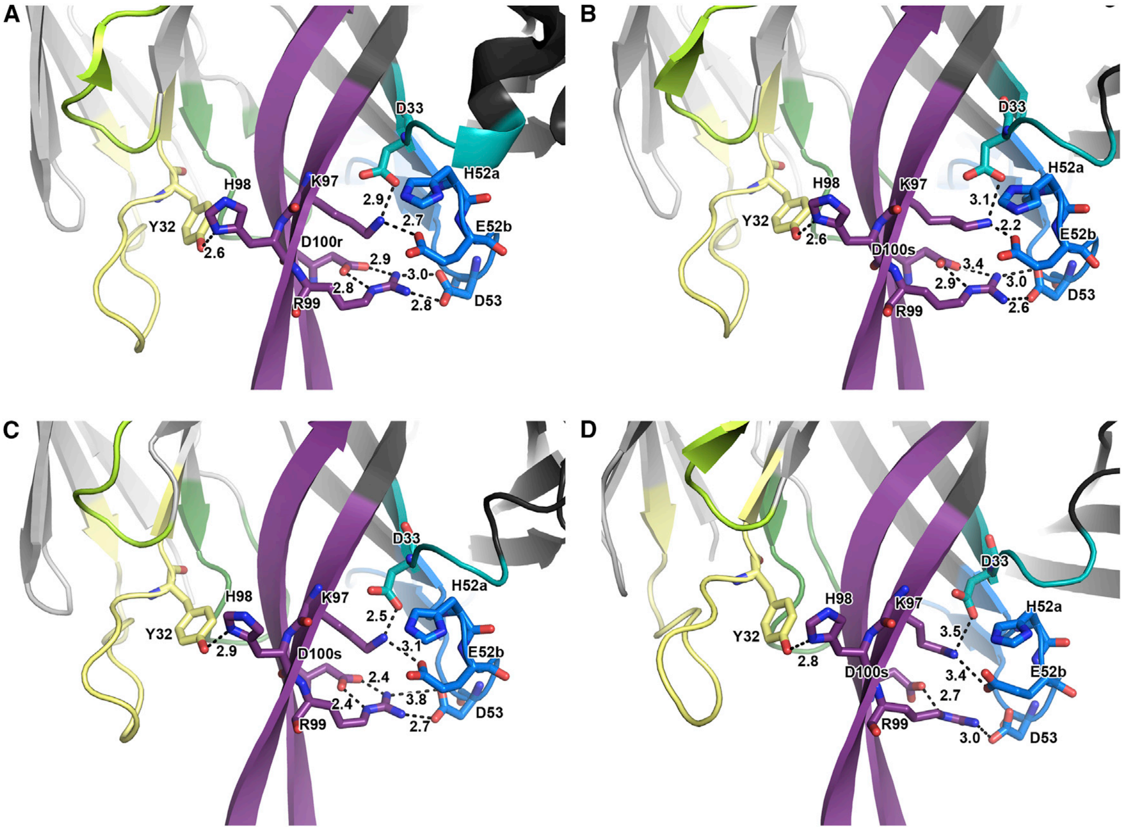

Figure 6. A Network of HCDR3 Stabilizing Interactions Is Found in Multiple PGT145-Class bnAbs

Inter-CDR stabilizing interactions (dashed lines, numbers corresponding to measured distance in Å) shown in Figure 5B are also present in the following unliganded X-ray Fab structures:

(A) PGT145.

(B) PGT144.

(C) PGT143.

(D) PGDM1400.

CDR loops are colored as in Figure 1A.

key specific peptide interactions that are conserved. Overall, our observations demonstrate that the potency of PGT145 is highly dependent on the CDR-stabilized HCDR3 structure and the acidic charge localization in the HCDR3 β-turn. We hypothesize that apex-directed bnAbs like PGT145 require less SHM to achieve breadth and potency because of the less-specific nature of binding to the apex; that is, the specific molecular determinants of binding are less stringent than for other bnAb classes. The steep angle of approach made possible by the long rigidified β-hairpin of HCDR3 also minimizes potential steric block by the hypervariable V1 and V2 loops. Viral fitness relies on preserving a fine balance between the closed and open conformations of the meta-stable, charged apical region of the Env trimer. By targeting these conserved properties, rather than sparsely conserved residues on the surface of Env, the PGT145-family of bnAbs have solved the HIV neutralization puzzle in a particularly creative fashion.

Our structural description of Env in complex with an apex targeting bnAb highlights the importance of an intact trimer apex for eliciting an appropriate immune response to this region. As germline targeting strategies gain more popularity the apex will likely require a trimeric immunogen that has the correct disposition of glycans and positive charges rather than a monomeric scaffold. Most rational vaccine design approaches involve engagement of appropriate germline precursors and biasing SHM to evolve specific molecular contacts with the antigen. The trimer apex presents a different challenge. Here, several mutations that do not contact the antigen are critical for bnAb evolution. One strategy for analyzing germline antibodies capable of evolving these types of responses would be to search for homologous HCDR3-stabilizing residues that reside in the other CDRs. These mutations can also act as early guideposts for evaluating if an immune response is going down the right path.

EXPERIMENTAL PROCEDURES

Protein Expression and Purification

Untagged or C-term His_6-tagged BG505 SOSIP.664 trimers were expressed in HEK293S or 293F cells and affinity purified using a 2G12 IgG cross-linked Sepharose column as described previously (Julien et al., 2013a). The affinity-purified trimers were size exclusion purified using a HiLoad 26/600 Superdex 200 pg column in 20 mM Tris pH 7.4, 150 mM NaCl (1× TBS), unless stated otherwise. The JR-FL EnvΔCT-PGT145 Fab complex was purified as

described using PGT145 as the pull-down reagent (Blattner et al., 2014). Antibody IgGs and Fabs were expressed in 293F cells and purified as previously described (Sok et al., 2014) (Supplemental Experimental Procedures).

Negative Stain EM Data Collection and Processing

All negative stain grids were prepared using 400 Cu mesh carbon coated grids as previously described (Julien et al., 2013b). PGT145 bound complexes were stained using NanoW for ~30 s, and unliganded trimers were stained using 2% uranyl formate for 45 s to 1 min. The EnvΔCT-PGT145 Fab complex was imaged on a Tecnai T12 coupled with a Tietz PXL 2k × 2k CCD camera, at a magnification of 52,000× resulting in 2.65 Å/pix images. Images were collected using Leginon (Suloway et al., 2005). Particles were processed as previously described (Blattner et al., 2014). Unliganded trimer mutants were imaged on a Tecnai T12 microscope coupled with a Tietz TemCam F416 CMOS detector, at a magnification of 52,000× resulting in 2.05 Å/pix on the specimen plane. Reference-free 2D classes were generated using MSA/MRA (Ogura et al., 2003) to sort the different trimer forms. Because these trimers were SEC purified over a Superdex 200 Increase 10/300 GL column, a large number of the mutants contained a significant portion of particles corresponding to monomers and dimers. Thus monomer and dimer populations were included in the analysis. In the first round of classification, all classes that had more than one particle in the boxed class average were eliminated. From a second round of 2D classification, reference-free 2D class averages were sub-grouped into three populations; (1) "non-native" that includes monomers, dimers, and badly assembled trimers, (2) "native-like closed," and (3) "native-like total" that includes both the closed (population [2]) and "breathing" ("native-like" but open) trimers (Pugach et al., 2015) (Supplemental Experimental Procedures).

CryoEM Data Collection and Processing

BG505 SOSIP.664 trimers produced in 293F cells were pre-complexed with 6-molar excess of 3BNC117 Fab overnight at 4°C, and size exclusion purified. To break the pseudo-symmetry of PGT145, PGT145 Fab was pre-complexed with a mouse Fab obtained from a commercial hybridoma cell line ATCC CRL-1757 (1757) that binds the HC of human Fabs (Figures S2A and S2B). To make the BG505 SOSIP.664-3BNC117-PGT145-1757 complex, PGT145 was combined with 1757 Fab (1:2 molar ratio) and size exclusion purified. The PGT145-1757 complex was then incubated with the purified BG505 SOSIP-3BNC117 complex and size exclusion purified. Samples were concentrated and manually frozen in liquid ethane. Data were collected via Leginon (Suloway et al., 2005) on an FEI Titan Krios electron microscope operating at 300 KeV coupled with a K2 Summit direct electron detector camera (Gatan) in counting mode at a magnification of 22,500× resulting in a pixel size of 1.31 Å/pixel, using a total dose of ~32 $e^{-}/Å^{2}$. Data were processed using RELION 1.4b1 (Scheres, 2012). The BG505 SOSIP.664-3BNC117 complex was refined using 22,625 particles with C3 symmetry imposed, to ~4.4 Å resolution at a Fourier shell correlation (FSC) cut-off of 0.143. 1757 was found to be specific for the constant region of the Fab HC, near the protein G binding site (Derrick and Wigley, 1994) (Figure S2F) allowing for confidence in the PGT145 Fab orientation. The BG505 SOSIP.664-3BNC117-PGT145-1757 and BG505 SOSIP.664-3BNC117-PGT145 classes from 3D sorting were combined, resulting in a total of 65,060 particles that were refined without imposing symmetry to 4.7 Å resolution (FSC = 0.143). A soft edge mask masking out 1757 Fab and PGT145 Fab constant domains was applied for one additional iteration of focused refinement, resulting in the ~4.3 Å resolution model (FSC = 0.143) (Figure S2D) (Supplemental Experimental Procedures).

Model Building and Refinement into the CryoEM Maps

The crystal structures of BG505 SOSIP.664 (4TVP) and 3BNC117 Fab (4JPV) were used as templates to generate an initial atomic model using the Modeller plug-in in UCSF Chimera (Pettersen et al., 2004; Webb and Sali, 2016). The resulting model was iteratively fixed and refined in Coot (Emsley et al., 2010) and RosettaRelax (DiMaio et al., 2009) employing Ramachandran constraints. Final models were chosen based on a combination of the Rosetta energy score, MolProbity and clash scores (Chen et al., 2010), and EMRinger score (Barad et al., 2015). Glycans were modeled into the finalized protein model as previously described (Lee et al., 2015), with all glycans being modeled as oligomannose. The protein structure in the BG505-3BNC117 model was used as an initial model to refine the PGT145-bound structure. The sulfated tyrosines in the PGT145 Fab X-ray structure were replaced with regular tyrosines because Rosetta fails to recognize sulfated tyrosines. The complete BG505-3BNC117-PGT145 complex was refined and followed by glycan modeling as was done for the BG505-3BNC117 complex (Supplemental Experimental Procedures).

Crystallization and X-ray Data Collection

PGT143 and PGT144 Fabs were concentrated to 4-24 mg/mL. Fab samples were screened for crystallization using the 384 conditions of the JCSG Core Suite at both 277 and 293 K using the TSRI/IAVI/JCSG robotic Crystalmation system as described previously (McLellan et al., 2011). Data collection was performed at cryogenic temperature (100 K) at beamline 23-ID of the Argonne Photon Source (APS), using a beam wavelength of 1.033 Å. The diffraction data were indexed, processed and scaled with HKL-2000 (Otwinowski and Minor, 1997) or XDS. Both structures were determined by molecular replacement using Phaser (McCoy et al., 2007) with PGT145 Fab as an initial model (3U1S). Model building and refinement was carried out using Coot-0.7 (Emsley et al., 2010) and Phenix (Adams et al., 2010) (Supplemental Experimental Procedures).

Surface Plasmon Resonance

SPR analysis of PGT145 Fab binding to His-tagged BG505 SOSIP trimers was analyzed on a Biacore 3000 instrument at 25°C. Glycan knockout mutants were expressed in HEK293F cells unless otherwise indicated. All trimers were immobilized on the chip by His-tag capture, as previously described (Yasmeen et al., 2014). To study interference or enhancement of CD4 and PGT145 binding, sequential binding analyses were also performed as stated previously (Derking et al., 2015) (Supplemental Experimental Procedures).

Neutralization Assays

For pseudovirus production, we cotransfected HEK293T or 293S cells with an Env encoding and an Env-deficient backbone (pSG3DEnv) plasmids using Fugene 6 (1:2 ratio). Pseudoviruses were harvested 48–72 hr post-transfection, filtered, and titrated for use in neutralization assays. Neutralization was measured in TZM-bl target cells, as described previously (Andrabi et al., 2015) (Supplemental Experimental Procedures).

Glycan Array Assays

mAbs were screened on a custom high mannose array, consisting of 9 mannosides and 1 control sialylated N-glycan. The 10 amine-linked glycans were covalently immobilized onto custom NHS-ester dendron functionalized glass microscope slides (G3 and G4, ZBiotech). To assess mAb binding, the antibodies were pre-mixed with the detection antibody (anti-human-IgG R-PE). Following 15 min, the pre-complexed antibodies were applied directly to the slide surface and allowed to incubate for 1 hr and then washed. Washed arrays were dried by centrifugation and then scanned for RPE signal on a confocal microarray scanner (Supplemental Experimental Procedures).

Identification of Key Residues by Regression Analysis

TZM-bl neutralization assay derived IC_{50} values of PGT145 from 135 strains was employed for linear regression analysis. Strains with IC_{50} > 10 μg/mL were classified as escape. Multiple sequence alignment was performed by MUSCLE using default parameters (Edgar, 2004). By comparing the amino acid identity of each residue of interest in each strain to that of BG505, a numeric value was assigned based on an adjusted BLOSUM62 matrix (Henikoff and Henikoff, 1992). For each substitution relative to a given amino acid at BG505, the numeric value was computed by subtracting the substitution score from the self-substitution score. No substitution is represented by a value of 0. A negative value would represent the conservativeness of the substitution, with less conservative being more negative. As a result, the amino-acid sequence for each strain was converted to a list of integers. Combining these sequences in the integer representation generated a matrix. A logistic regression model with L1 regularization was then fit to the matrix with the escape phenotype as the targets. Logistic regression was performed using "linear_model.LogisticRegressionCV" in scikit-learn (Pedregosa et al., 2011) in python. Each residue of interest would be assigned a coefficient. A larger magnitude of coefficient a residue implied more influence it has on the escape

phenotype. The absolute value of the coefficient was reported (Supplemental Experimental Procedures).

ACCESSION NUMBERS

Atomic coordinates and structure factors for the X-ray structures have been deposited with the Protein Data Bank under accession codes 5UXQ (PGT143) and 5UY3 (PGT144). The cryoEM maps and models have been deposited with the Electron Microscopy Data Bank and the Protein Data Bank under accession codes EMD-8644, 5V8M (BG505 SOSIP.664-3BNC117) and EMD-8643, 5V8L (BG505 SOSIP.664-3BNC117-PGT145).

SUPPLEMENTAL INFORMATION

Supplemental Information includes seven figures and Supplemental Experimental Procedures and can be found with this article online at http://dx.doi.org/10.1016/j.immuni.2017.03.017.

AUTHOR CONTRIBUTIONS

J.H.L, R.A., and J.-P.J. designed the project. J.H.L. performed the EM work and structural analysis. J.-P.J. and L.K. performed the crystallization studies. J.H.L. and C.A.C. performed atomic modeling of the EM structures. R.A., C.S., D.S., and M.P. performed the neutralization assays. A.Y. and P.J.K. performed the SPR studies. R.M. performed the glycan array assays. N.C.W. performed the linear regression analysis. T.N. and C.B. provided reagents. J.H.L., R.A., P.J.K., D.R.B., and A.B.W. analyzed the data. R.A. contributed to figure generation. J.H.L., A.B.W., D.R.B., and I.A.W. wrote the manuscript. All authors were asked to comment on the manuscript.

ACKNOWLEDGMENTS

This work was supported by the California HIV/AIDS Research Program Dissertation Award D12-SRI-353 (to J.H.L.), the Scripps CHAVI-ID (UM1 AI100663), P01 AI110657, International AIDS Vaccine Initiative, and the Bill and Melinda Gates Foundation CAVD (OPP1115782 and OPP1084519). GM/CA@APS has been funded in whole or in part with Federal funds from the National Cancer Institute (ACB-12002) and the National Institute of General Medical Sciences (AGM-12006). This research used resources of the Advanced Photon Source, a U.S. Department of Energy (DOE) Office of Science User Facility operated for the DOE Office of Science by Argonne National Laboratory under Contract No. DE-AC02-06CH11357. This is manuscript number 29440 from the Scripps Research Institute.

Received: December 21, 2016
Revised: February 23, 2017
Accepted: March 24, 2017
Published: April 18, 2017

REFERENCES

Adams, P.D., Afonine, P.V., Bunkoczi, G., Chen, V.B., Davis, I.W., Echols, N., Headd, J.J., Hung, L.W., Kapral, G.J., Grosse-Kunstleve, R.W., et al. (2010). PHENIX: a comprehensive Python-based system for macromolecular structure solution. Acta Crystallogr., Sect: D Biol. Crystallogr. *66*, 213–221.

Andrabi, R., Voss, J.E., Liang, C.H., Briney, B., McCoy, L.E., Wu, C.Y., Wong, C.H., Poignard, P., and Burton, D.R. (2015). Identification of common features in prototype broadly neutralizing antibodies to HIV envelope V2 apex to facilitate vaccine design. Immunity *43*, 959–973.

Barad, B.A., Echols, N., Wang, R.Y., Cheng, Y., DiMaio, F., Adams, P.D., and Fraser, J.S. (2015). EMRinger: side chain-directed model and map validation for 3D cryo-electron microscopy. Nat. Methods *12*, 943–946.

Blattner, C., Lee, J.H., Sliepen, K., Derking, R., Falkowska, E., de la Peña, A.T., Cupo, A., Julien, J.P., van Gils, M., Lee, P.S., et al. (2014). Structural delineation of a quaternary, cleavage-dependent epitope at the gp41-gp120 interface on intact HIV-1 Env trimers. Immunity *40*, 669–680.

Burton, D.R., and Hangartner, L. (2016). Broadly neutralizing antibodies to HIV and their role in vaccine design. Annu. Rev. Immunol. *34*, 635–659.

Chen, V.B., Arendall, W.B., 3rd, Headd, J.J., Keedy, D.A., Immormino, R.M., Kapral, G.J., Murray, L.W., Richardson, J.S., and Richardson, D.C. (2010). MolProbity: all-atom structure validation for macromolecular crystallography. Acta Crystallogr. Sect: D. Biol. Crystallogr. *66*, 12–21.

Cheng, C., Pancera, M., Bossert, A., Schmidt, S.D., Chen, R.E., Chen, X., Druz, A., Narpala, S., Doria-Rose, N.A., McDermott, A.B., et al. (2015). Immunogenicity of a prefusion HIV-1 envelope trimer in complex with a quaternary-structure-specific antibody. J. Virol. *90*, 2740–2755.

Curlin, M.E., Zioni, R., Hawes, S.E., Liu, Y., Deng, W., Gottlieb, G.S., Zhu, T., and Mullins, J.I. (2010). HIV-1 envelope subregion length variation during disease progression. PLoS Pathog. *6*, e1001228.

de Taeye, S.W., Ozorowski, G., Torrents de la Peña, A., Guttman, M., Julien, J.P., van den Kerkhof, T.L., Burger, J.A., Pritchard, L.K., Pugach, P., Yasmeen, A., et al. (2015). Immunogenicity of stabilized HIV-1 envelope trimers with reduced exposure of non-neutralizing epitopes. Cell *163*, 1702–1715.

Derking, R., Ozorowski, G., Sliepen, K., Yasmeen, A., Cupo, A., Torres, J.L., Julien, J.P., Lee, J.H., van Montfort, T., de Taeye, S.W., et al. (2015). Comprehensive antigenic map of a cleaved soluble HIV-1 envelope trimer. PLoS Pathog. *11*, e1004767.

Derrick, J.P., and Wigley, D.B. (1994). The third IgG-binding domain from streptococcal protein G. An analysis by X-ray crystallography of the structure alone and in a complex with Fab. J. Mol. Biol. *243*, 906–918.

DiMaio, F., Tyka, M.D., Baker, M.L., Chiu, W., and Baker, D. (2009). Refinement of protein structures into low-resolution density maps using rosetta. J. Mol. Biol. *392*, 181–190.

Doria-Rose, N.A., Georgiev, I., O'Dell, S., Chuang, G.Y., Staupe, R.P., McLellan, J.S., Gorman, J., Pancera, M., Bonsignori, M., Haynes, B.F., et al. (2012). A short segment of the HIV-1 gp120 V1/V2 region is a major determinant of resistance to V1/V2 neutralizing antibodies. J. Virol. *86*, 8319–8323.

Doria-Rose, N.A., Schramm, C.A., Gorman, J., Moore, P.L., Bhiman, J.N., DeKosky, B.J., Ernandes, M.J., Georgiev, I.S., Kim, H.J., Pancera, M., et al.; NISC Comparative Sequencing Program (2014). Developmental pathway for potent V1V2-directed HIV-neutralizing antibodies. Nature *509*, 55–62.

Doria-Rose, N.A., Bhiman, J.N., Roark, R.S., Schramm, C.A., Gorman, J., Chuang, G.Y., Pancera, M., Cale, E.M., Ernandes, M.J., Louder, M.K., et al. (2015). New member of the V1V2-directed CAP256-VRC26 lineage that shows increased breadth and exceptional potency. J. Virol. *90*, 76–91.

Edgar, R.C. (2004). MUSCLE: multiple sequence alignment with high accuracy and high throughput. Nucleic Acids Res. *32*, 1792–1797.

Ekiert, D.C., Kashyap, A.K., Steel, J., Rubrum, A., Bhabha, G., Khayat, R., Lee, J.H., Dillon, M.A., O'Neil, R.E., Faynboym, A.M., et al. (2012). Cross-neutralization of influenza A viruses mediated by a single antibody loop. Nature *489*, 526–532.

Emsley, P., Lohkamp, B., Scott, W.G., and Cowtan, K. (2010). Features and development of Coot. Acta Crystallogr., Sect: D Biol. Crystallogr. *66*, 486–501.

Gorman, J., Soto, C., Yang, M.M., Davenport, T.M., Guttman, M., Bailer, R.T., Chambers, M., Chuang, G.Y., DeKosky, B.J., Doria-Rose, N.A., et al.; NISC Comparative Sequencing Program (2016). Structures of HIV-1 Env V1V2 with broadly neutralizing antibodies reveal commonalities that enable vaccine design. Nat. Struct. Mol. Biol. *23*, 81–90.

Henikoff, S., and Henikoff, J.G. (1992). Amino acid substitution matrices from protein blocks. Proc. Natl. Acad. Sci. USA *89*, 10915–10919.

Julien, J.P., Cupo, A., Sok, D., Stanfield, R.L., Lyumkis, D., Deller, M.C., Klasse, P.J., Burton, D.R., Sanders, R.W., Moore, J.P., et al. (2013a). Crystal structure of a soluble cleaved HIV-1 envelope trimer. Science *342*, 1477–1483.

Julien, J.P., Lee, J.H., Cupo, A., Murin, C.D., Derking, R., Hoffenberg, S., Caulfield, M.J., King, C.R., Marozsan, A.J., Klasse, P.J., et al. (2013b). Asymmetric recognition of the HIV-1 trimer by broadly neutralizing antibody PG9. Proc. Natl. Acad. Sci. USA *110*, 4351–4356.

Klein, F., Diskin, R., Scheid, J.F., Gaebler, C., Mouquet, H., Georgiev, I.S., Pancera, M., Zhou, T., Incesu, R.B., Fu, B.Z., et al. (2013). Somatic mutations

of the immunoglobulin framework are generally required for broad and potent HIV-1 neutralization. Cell *153*, 126–138.

Kwon, Y.D., Pancera, M., Acharya, P., Georgiev, I.S., Crooks, E.T., Gorman, J., Joyce, M.G., Guttman, M., Ma, X., Narpala, S., et al. (2015). Crystal structure, conformational fixation and entry-related interactions of mature ligand-free HIV-1 Env. Nat. Struct. Mol. Biol. *22*, 522–531.

Lee, J.H., de Val, N., Lyumkis, D., and Ward, A.B. (2015). Model Building and Refinement of a Natively Glycosylated HIV-1 Env protein by high-resolution cryoelectron microscopy. Structure *23*, 1943–1951.

Liu, J., Bartesaghi, A., Borgnia, M.J., Sapiro, G., and Subramaniam, S. (2008). Molecular architecture of native HIV-1 gp120 trimers. Nature *455*, 109–113.

McCoy, A.J., Grosse-Kunstleve, R.W., Adams, P.D., Winn, M.D., Storoni, L.C., and Read, R.J. (2007). Phaser crystallographic software. J. Appl. Cryst. *40*, 658–674.

McLellan, J.S., Pancera, M., Carrico, C., Gorman, J., Julien, J.P., Khayat, R., Louder, R., Pejchal, R., Sastry, M., Dai, K., et al. (2011). Structure of HIV-1 gp120 V1/V2 domain with broadly neutralizing antibody PG9. Nature *480*, 336–343.

Ogura, T., Iwasaki, K., and Sato, C. (2003). Topology representing network enables highly accurate classification of protein images taken by cryo electron-microscope without masking. J. Struct. Biol. *143*, 185–200.

Otwinowski, Z., and Minor, W. (1997). Processing of X-ray diffraction data collected in oscillation mode. Methods Enzymol. *276A*, 307–326.

Pancera, M., Shahzad-Ul-Hussan, S., Doria-Rose, N.A., McLellan, J.S., Bailer, R.T., Dai, K., Loesgen, S., Louder, M.K., Staupe, R.P., Yang, Y., et al. (2013). Structural basis for diverse N-glycan recognition by HIV-1-neutralizing V1-V2-directed antibody PG16. Nat. Struct. Mol. Biol. *20*, 804–813.

Pedregosa, F., Varoquaux, G., Gramfort, A., Michel, V., Thirion, B., Grisel, O., Blondel, M., Prettenhofer, P., Weiss, R., Dubourg, V., et al. (2011). Scikit-learn: Machine Learning in Python. J. Mach. Learn. Res. *12*, 2825–2830.

Pettersen, E.F., Goddard, T.D., Huang, C.C., Couch, G.S., Greenblatt, D.M., Meng, E.C., and Ferrin, T.E. (2004). UCSF Chimera–a visualization system for exploratory research and analysis. J. Comput. Chem. *25*, 1605–1612.

Pugach, P., Ozorowski, G., Cupo, A., Ringe, R., Yasmeen, A., de Val, N., Derking, R., Kim, H.J., Korzun, J., Golabek, M., et al. (2015). A native-like SOSIP.664 trimer based on an HIV-1 subtype B env gene. J. Virol. *89*, 3380–3395.

Rizzuto, C.D., Wyatt, R., Hernández-Ramos, N., Sun, Y., Kwong, P.D., Hendrickson, W.A., and Sodroski, J. (1998). A conserved HIV gp120 glycoprotein structure involved in chemokine receptor binding. Science *280*, 1949–1953.

Rusert, P., Kouyos, R.D., Kadelka, C., Ebner, H., Schanz, M., Huber, M., Braun, D.L., Hozé, N., Scherrer, A., Magnus, C., et al.; Swiss HIV Cohort Study (2016). Determinants of HIV-1 broadly neutralizing antibody induction. Nat. Med. *22*, 1260–1267.

Sagar, M., Wu, X., Lee, S., and Overbaugh, J. (2006). Human immunodeficiency virus type 1 V1-V2 envelope loop sequences expand and add glycosylation sites over the course of infection, and these modifications affect antibody neutralization sensitivity. J. Virol. *80*, 9586–9598.

Sanders, R.W., Derking, R., Cupo, A., Julien, J.P., Yasmeen, A., de Val, N., Kim, H.J., Blattner, C., de la Peña, A.T., Korzun, J., et al. (2013). A next-generation cleaved, soluble HIV-1 Env trimer, BG505 SOSIP.664 gp140, expresses multiple epitopes for broadly neutralizing but not non-neutralizing antibodies. PLoS Pathog. *9*, e1003618.

Scharf, L., West, A.P., Sievers, S.A., Chen, C., Jiang, S., Gao, H., Gray, M.D., McGuire, A.T., Scheid, J.F., Nussenzweig, M.C., et al. (2016). Structural basis for germline antibody recognition of HIV-1 immunogens. eLife *5*, 5.

Scheres, S.H. (2012). RELION: implementation of a Bayesian approach to cryo-EM structure determination. J. Struct. Biol. *180*, 519–530.

Sok, D., van Gils, M.J., Pauthner, M., Julien, J.P., Saye-Francisco, K.L., Hsueh, J., Briney, B., Lee, J.H., Le, K.M., Lee, P.S., et al. (2014). Recombinant HIV envelope trimer selects for quaternary-dependent antibodies targeting the trimer apex. Proc. Natl. Acad. Sci. USA *111*, 17624–17629.

Steichen, J.M., Kulp, D.W., Tokatlian, T., Escolano, A., Dosenovic, P., Stanfield, R.L., McCoy, L.E., Ozorowski, G., Hu, X., Kalyuzhniy, O., et al. (2016). HIV vaccine design to target germline precursors of glycan-dependent broadly neutralizing antibodies. Immunity *45*, 483–496.

Suloway, C., Pulokas, J., Fellmann, D., Cheng, A., Guerra, F., Quispe, J., Stagg, S., Potter, C.S., and Carragher, B. (2005). Automated molecular microscopy: the new Leginon system. J. Struct. Biol. *151*, 41–60.

Walker, L.M., Phogat, S.K., Chan-Hui, P.Y., Wagner, D., Phung, P., Goss, J.L., Wrin, T., Simek, M.D., Fling, S., Mitcham, J.L., et al.; Protocol G Principal Investigators (2009). Broad and potent neutralizing antibodies from an African donor reveal a new HIV-1 vaccine target. Science *326*, 285–289.

Walker, L.M., Huber, M., Doores, K.J., Falkowska, E., Pejchal, R., Julien, J.P., Wang, S.K., Ramos, A., Chan-Hui, P.Y., Moyle, M., et al.; Protocol G Principal Investigators (2011). Broad neutralization coverage of HIV by multiple highly potent antibodies. Nature *477*, 466–470.

Wang, S.K., Liang, P.H., Astronomo, R.D., Hsu, T.L., Hsieh, S.L., Burton, D.R., and Wong, C.H. (2008). Targeting the carbohydrates on HIV-1: Interaction of oligomannose dendrons with human monoclonal antibody 2G12 and DC-SIGN. Proc. Natl. Acad. Sci. USA *105*, 3690–3695.

Wang, F., Ekiert, D.C., Ahmad, I., Yu, W., Zhang, Y., Bazirgan, O., Torkamani, A., Raudsepp, T., Mwangi, W., Criscitiello, M.F., et al. (2013). Reshaping antibody diversity. Cell *153*, 1379–1393.

Webb, B., and Sali, A. (2016). Comparative Protein Structure Modeling Using MODELLER. Curr. Protoc. Bioinformatics *54*, 5 6 1-5 6 37.

Wu, L., Gerard, N.P., Wyatt, R., Choe, H., Parolin, C., Ruffing, N., Borsetti, A., Cardoso, A.A., Desjardin, E., Newman, W., et al. (1996). CD4-induced interaction of primary HIV-1 gp120 glycoproteins with the chemokine receptor CCR-5. Nature *384*, 179–183.

Yacoob, C., Pancera, M., Vigdorovich, V., Oliver, B.G., Glenn, J.A., Feng, J., Sather, D.N., McGuire, A.T., and Stamatatos, L. (2016). Differences in allelic frequency and CDRH3 region limit the engagement of HIV env immunogens by putative VRC01 neutralizing antibody precursors. Cell Rep. *17*, 1560–1570.

Yasmeen, A., Ringe, R., Derking, R., Cupo, A., Julien, J.P., Burton, D.R., Ward, A.B., Wilson, I.A., Sanders, R.W., Moore, J.P., and Klasse, P.J. (2014). Differential binding of neutralizing and non-neutralizing antibodies to native-like soluble HIV-1 Env trimers, uncleaved Env proteins, and monomeric subunits. Retrovirology *11*, 41.

Immunity, Volume *46*

Supplemental Information

A Broadly Neutralizing Antibody Targets the Dynamic HIV Envelope Trimer Apex via a Long, Rigidified, and Anionic β-Hairpin Structure

Jeong Hyun Lee, Raiees Andrabi, Ching-Yao Su, Anila Yasmeen, Jean-Philippe Julien, Leopold Kong, Nicholas C. Wu, Ryan McBride, Devin Sok, Matthias Pauthner, Christopher A. Cottrell, Travis Nieusma, Claudia Blattner, James C. Paulson, Per Johan Klasse, Ian A. Wilson, Dennis R. Burton, and Andrew B. Ward

SUPPLEMENTARY FIGURES

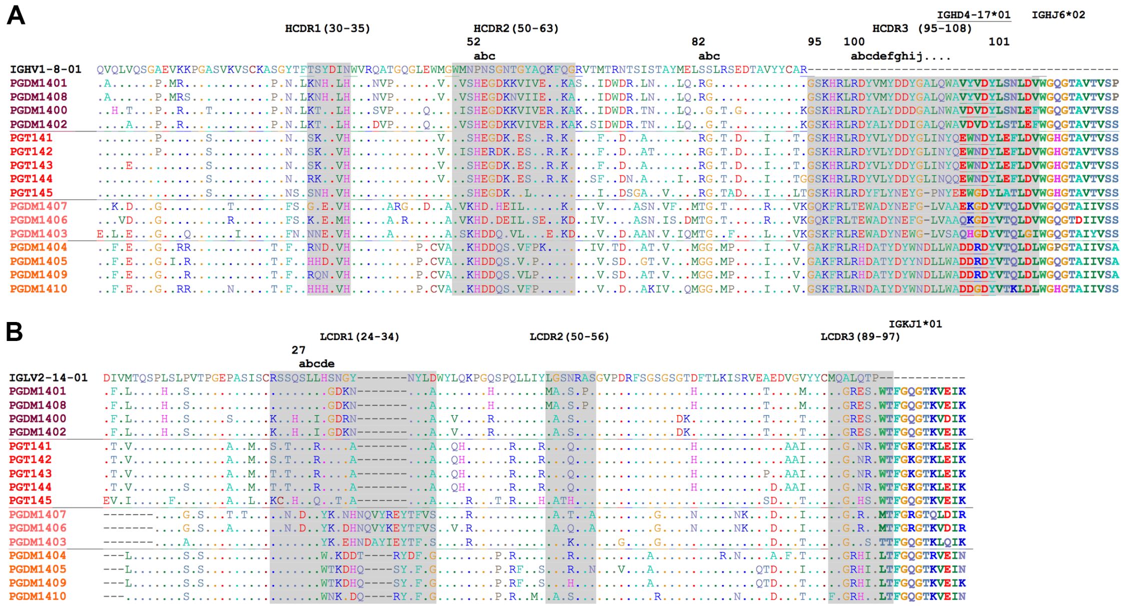

Figure S1. Sequence alignment of PGT145-family Abs. Related to Figure 1 and Table 1.

(A) Sequence alignment of HC variable regions of PGT145-family antibodies. The predicted germline gene is shown at the top. The insertions in CDRs are listed alphabetically (e.g. a,b,c, etc.). Due to the shorter HCDR3 in PGT145, PGDM1407, PGDM1406 and PGDM1403, the insertions beyond 100j are not listed in this figure.

(B) Sequence alignment of LC variable regions of PGT145-family antibodies.

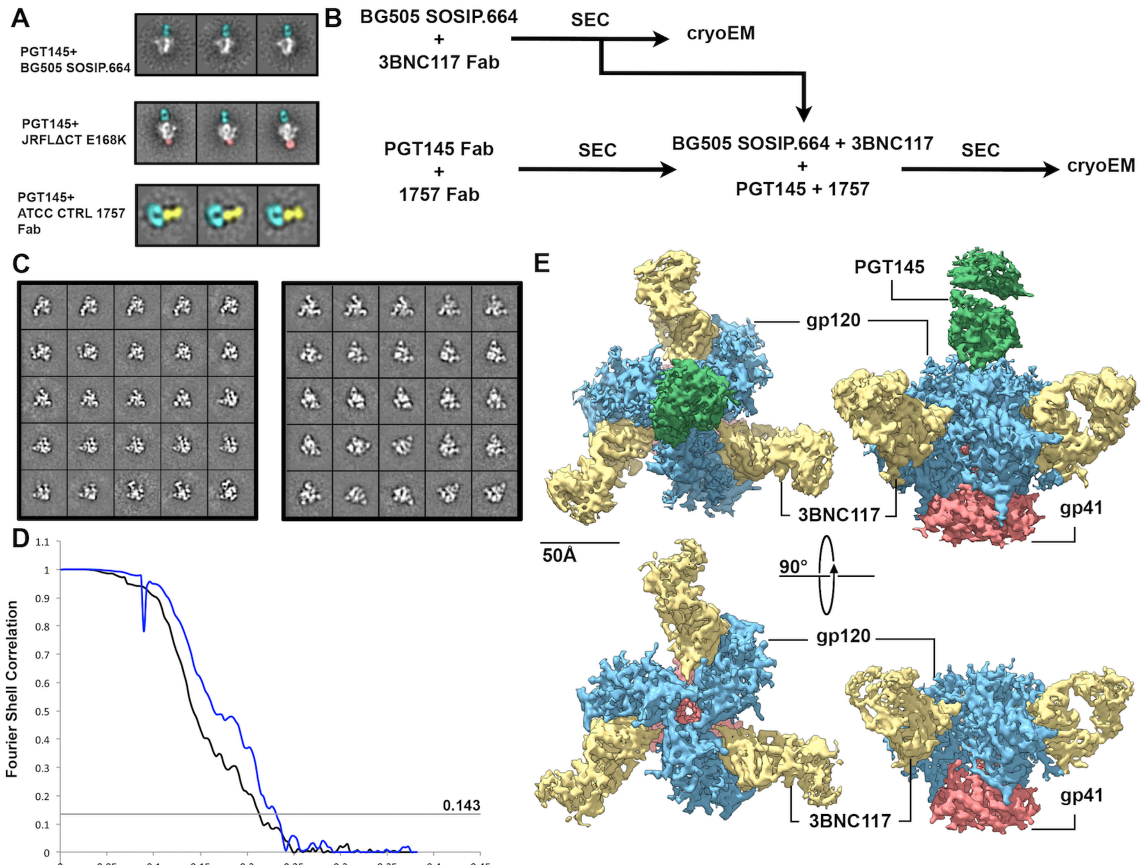

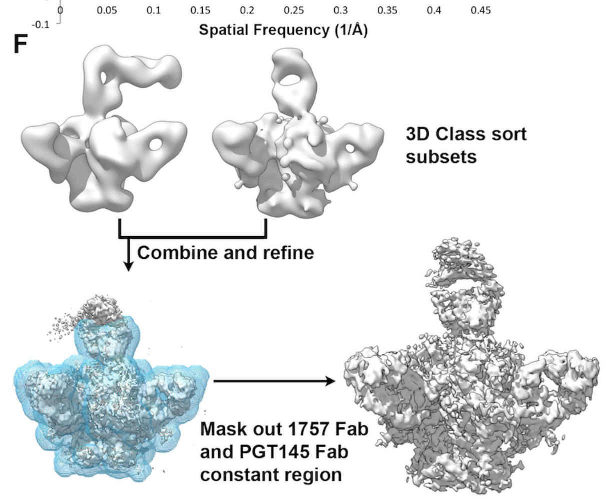

G

	BG505.664 in complex with 3BNC117	BG505.664 in complex with 3BNC117 and PGT145
Clashscore, all atoms	5.94 (91th percentile, all resolutions)	6.83 (87th percentile, all resolutions)
Poor rotamers	0.14%	0.30%
Favored rotamers	99.43%	98.70%
Ramachandran outliers	0%	0.19%
Ramachandran favored	97.74%	98.07%
MolProbity score	1.40 (97th percentile, all resolutions)	1.38 (98th percentile, all resolutions)
Cβ deviations	0%	0.04%
Bad bonds	0.24%	0.24%
Bad angles	0.33%	0.44%
Cis prolines	0	2
EMRinger score	1.41	1.33

Figure S2. Single particle EM analysis of the 3BNC117-BG505 SOSIP.664 and PGT145-3BNC117-BG505 SOSIP.664 complexes. Related to Figure 1.

(A) Reference free negative stain 2D class averages of BG505 SOSIP.664-PGT145 Fab complex, JR-FL EnvΔCT E168K-PGT145 Fab, and PGT145 Fab-1757 Fab complex. PGT145 and 1757 Fabs are false colored in blue and yellow, respectively. In the JR-FL EnvΔCT sample, the transmembrane domain is colored in pink.

(B) Purification scheme for the two complexes studied by cryoEM.

(C) Reference free 2D class averages of the BG505-3BNC117-PGT145-1757 complex (left), and BG505-3BNC117 complex (right).

(D) Fourier shell correlation curves of the final reconstructions of the 3BNC117-PGT145 (blue), and 3BNC117 (black) bound complexes.

(E) Segmented top (left) and side views (center) of the cryoEM reconstruction of BG505 SOSIP.664-3BNC117-PGT145 (top), and BG505 SOSIP.664-3BNC117 (bottom) complexes, colored according to subunit identity. Scale bar is shown.

(F) Sorting and refinement strategy of the BG505 SOSIP.664-3BNC117-PGT145-1757 complex. The blue mesh corresponds to the mask applied for final focused refinement iterations.

(G) MolProbity and EM Ringer statistics for the refined models.

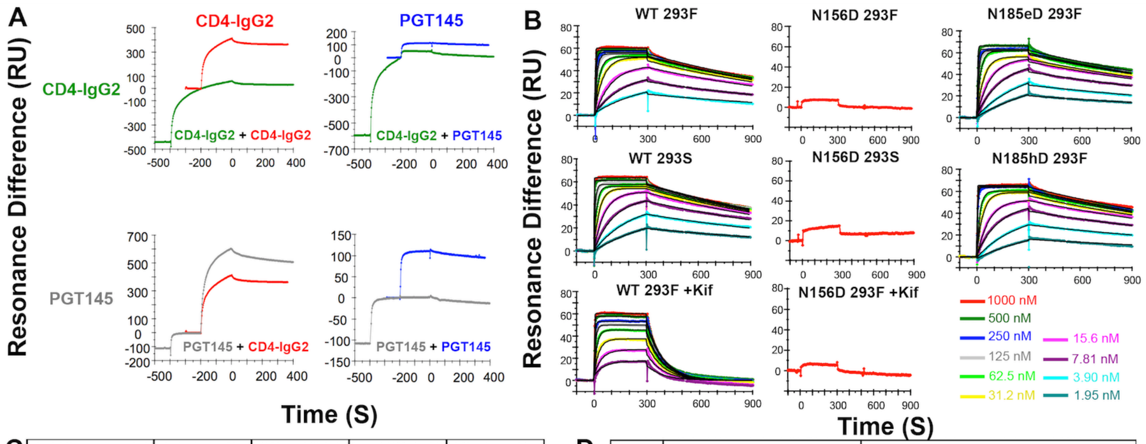

C

	k_{on} (1/Ms)	k_{off} (1/s)	K_d (nM)	S_m
**WT 293F** (n=2)	$6.1 \cdot 10^5$ $\pm 2.5 \cdot 10^4$	$7.7 \cdot 10^{-4}$ $\pm 5.5 \cdot 10^{-5}$	1.3 $\pm 5.0 \cdot 10^{-2}$	0.87 $\pm 2.1 \cdot 10^{-2}$
**WT 293S** (n=2)	$1.1 \cdot 10^6$ $\pm 5.0 \cdot 10^4$	$7.8 \cdot 10^{-4}$ $\pm 2.0 \cdot 10^{-5}$	0.76 $\pm 3.5 \cdot 10^{-2}$	0.90 $\pm 1.8 \cdot 10^{-3}$
**WT 293F Kif** (n=2)	$7.8 \cdot 10^5$ $\pm 1.0 \cdot 10^4$	$1.1 \cdot 10^{-2}$ ± 0	14 ± 0.50	0.79 $\pm 3.8 \cdot 10^{-3}$
**N185eD 293F** (n=2)	$7.7 \cdot 10^5$ $\pm 5.0 \cdot 10^3$	$6.2 \cdot 10^{-4}$ $\pm 5.0 \cdot 10^{-6}$	0.81 $\pm 1.5 \cdot 10^{-2}$	0.97 $\pm 8.9 \cdot 10^{-3}$
**N185hD 293F** (n=2)	$9.1 \cdot 10^5$ $\pm 1.5 \cdot 10^4$	$6.5 \cdot 10^{-4}$ $\pm 1.5 \cdot 10^{-5}$	0.72 $\pm 5.0 \cdot 10^{-3}$	0.96 $\pm 2.1 \cdot 10^{-3}$

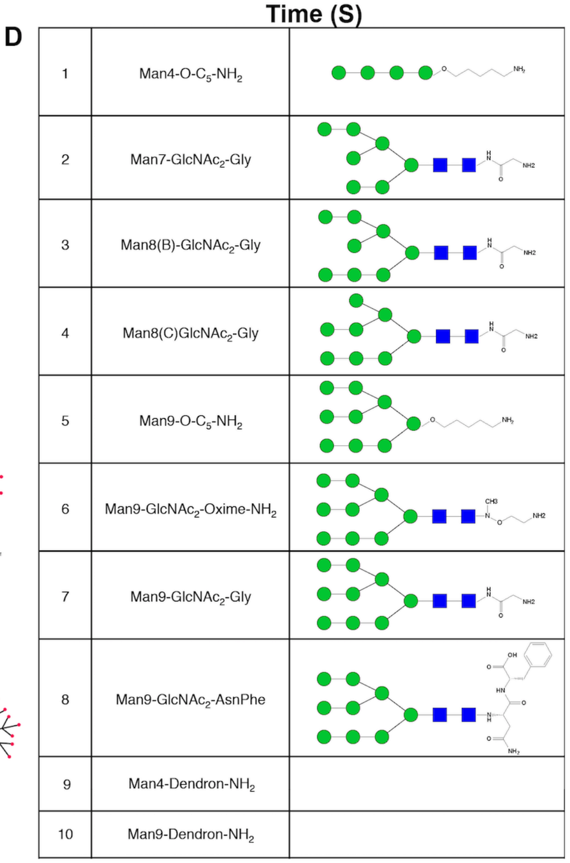

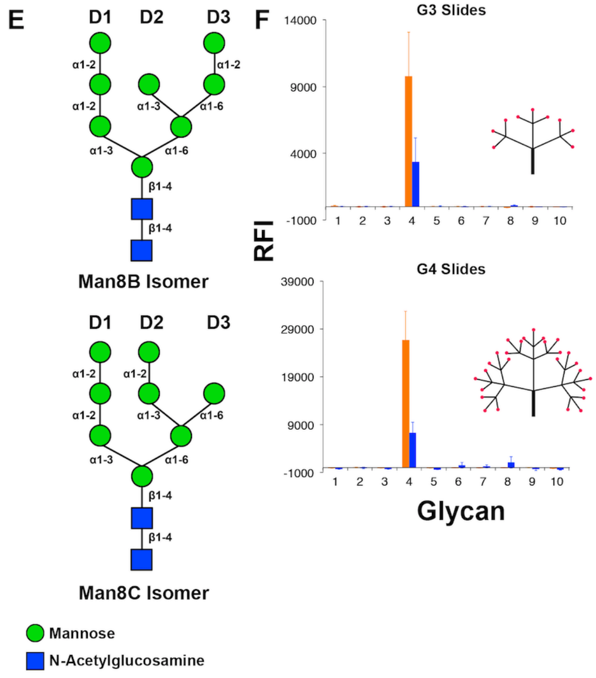

Figure S3. SPR analysis of PGT145 binding. Related to Figures 1 and 2.

(A) SPR sensograms showing association and dissociation phases of binding to PGT145 or CD4IgG2 in the presence of the other.

(B) SPR analysis of PGT145 binding to BG505 SOSIP.664 trimers produced in wild-type glycan producing 293F, 293S, or 293F cells in the presence of Kif, and glycan knockouts at the apex.

(C) SPR kinetic parameters of PGT145 Fab binding to various BG505 SOSIP.664 variants performed in (B). Means of n replicates and ± s.e.m. are given.

(D) The range of glycans tested on the oligomannose glycan array and their structures. The Man_4- and Man_9-Dendron-NH_2 are oligomannose residues pre-attached to a dendron prior to attachment to the G3 or G4 dendrimer slides.

(E) Definition of the M8B and M8C isomers and oligomannose branching nomenclature.

(F) PGT145 (blue) and PGDM1400 (orange) binding to the glycans shown in (E) on G3 (top) or G4 (bottom) slides. Error bars indicate s.e.m. To the right of the bar graphs show the dendrimer branching scheme for the G3 and G4 slides, with red dots indicate where glycans would be attached. Dendron image is not to scale.

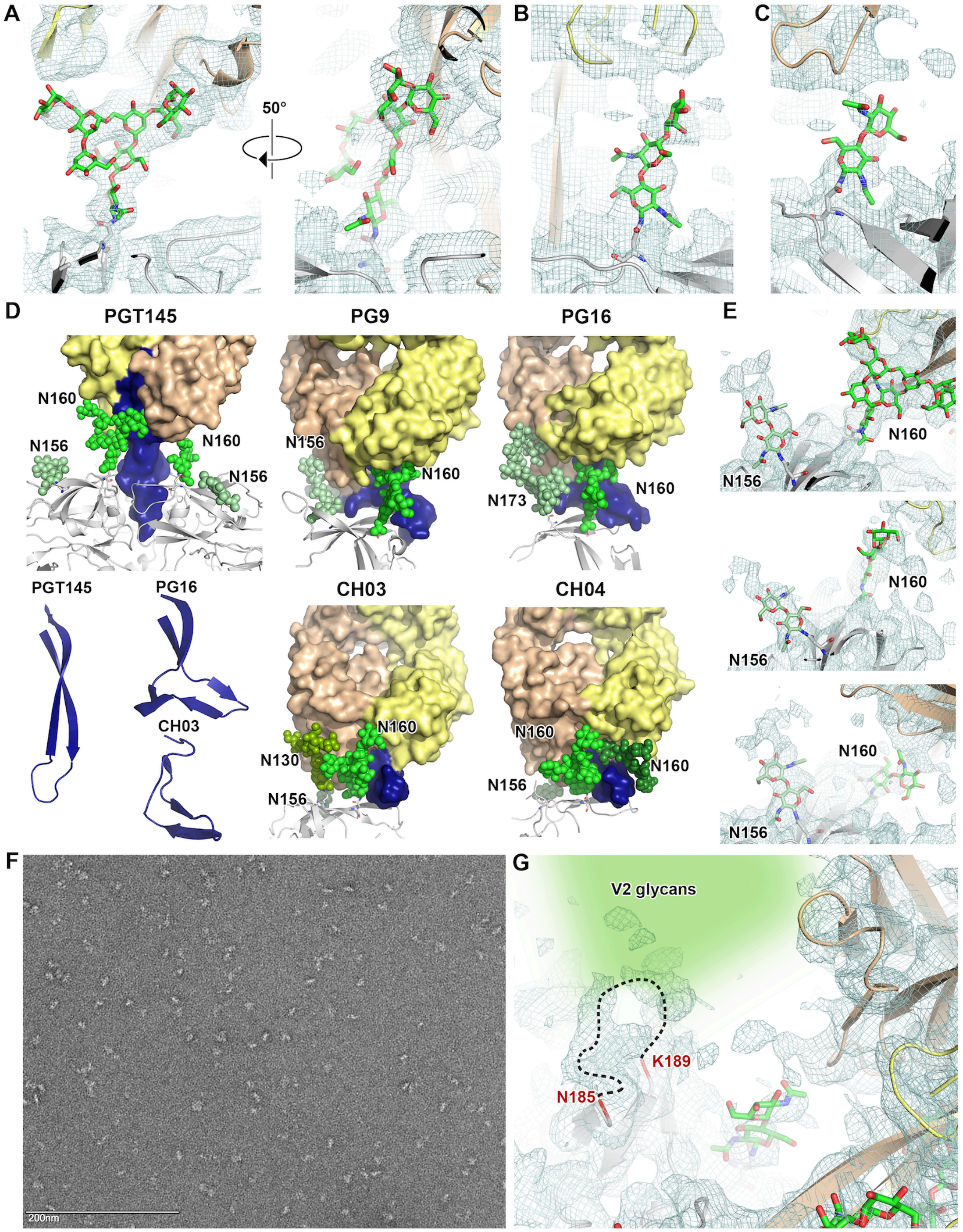

Figure S4. N160 glycan densities and comparison of glycan recognition by apex bnAbs. Related to Figure 3.

The modeled N160 glycans (green) and the cryoEM map (blue mesh). PGT145 HC and LC are shown in tan and yellow, respectively. Gp120 is shown in gray.

(A) $N160_{glycan1}$ in the BG505-PGT145 complex map.

(B) $N160_{glycan2}$ in the BG505-PGT145 complex map.

(C) $N160_{glycan3}$ in the BG505-PGT145 complex map.

(D) V2 binding antibodies PGT145, PG9 (3U4E), PG16 (4DQO), CH03 (5ESV) and CH04 (5ESZ), and their interaction with apex glycans. The N173 glycan is a compensatory glycan for N156. CH04 interacts with an additional glycan (dark green) predicted to represent the N160 glycan from an adjacent protomer. A comparison of the HCDR3 structural conformations is shown in the bottom left. The light and heavy chains are colored as in (A), with the HCDR3 shown in blue.

(E) The N156 glycans face away from PGT145. The figure is colored as in (A). The EM map is contoured at $\sigma=5.5$.

(F) A negative stain EM micrograph of BG505 SOSIP.664 N156D trimers. Very few intact trimers are visible in the image.

(G) The dashed line indicates the complete trajectory of the V2 loop. The two glycans in V2 of BG505 likely occupy space (shaded region) near the PGT145 antibody (tan). The EM map is contoured at $\sigma=5.5$ and glycans are shown as green sticks.

A

BG505.Env.C2	PGDM1400	PGDM1401	PGDM1404	PGDM1406	PGDM1409	PGT143	PGT145	PG9	CH01	CAP256.09	VRC01
K121A	3.6	608.7	>3	NA	>17	>353	>302	0.6	0.2	9.0	0.642
V127A	>613	>1072	>3	NA	>17	>353	>302	12.9	17.3	>226	1.535
N156K	144.0	>1072	>3	NA	>17	>353	>302	44.5	17.3	>226	0.967
N160K	>613	>1072	>3	NA	>17	>353	>302	>309	17.3	>226	0.987
N160A	>613	>1072	>3	NA	>17	>353	>302	>309	17.3	>226	0.223
M161A	2.5	15.9	>3	NA	>17	22.2	60.4	1.2	0.6	0.2	0.553
T162A	>613	>1072	>3	NA	>17	>353	>302	>309	17.3	>226	0.674
E164A	0.7	0.8	>3	NA	10.9	0.8	2.7	0.5	0.7	33.7	0.705
L165A	2.4	923.0	>3	NA	>17	152.4	167.1	0.5	0.3	>226	0.421
R166A	>613	>1072	>3	NA	>17	>353	>302	0.8	0.7	>226	1.225
D167A	>613	>1072	>3	NA	>17	>353	>302	0.5	0.3	>226	0.422
K168A	48.3	556.7	>3	NA	>17	155.3	274.8	18.1	4.3	4.3	0.579
K169A	75.9	12.2	>3	NA	>17	3.0	256.2	1.7	2.9	>226	0.942
I307A	0.9	0.7	>3	NA	>17	2.0	0.3	0.7	0.4	9.4	1.043
I420A	1.8	3.2	>3	NA	8.6	3.3	3.8	0.9	1.1	2.4	0.937
I423A	0.6	0.2	>3	NA	16.5	1.2	1.9	1.7	1.7	11.7	0.902

B

Mutation	% Non-Native	% Native-like Closed	% Native-like Total
I420A	100	0	0
L165A	80	19	20
D167A	70	26	30
I307A	58	42	42
I432A	46	36	54
K169A	39	11	61
M161A	38	34	62
T162A	36	53	64
P124A	34	24	66
K121A	30	25	70
R166A	28	56	72
V127A	26	43	74
T123A	21	59	79
WT	19	46	81
E164A	11	40	89
N160A	0	81	100
K168A	0	76	100

C

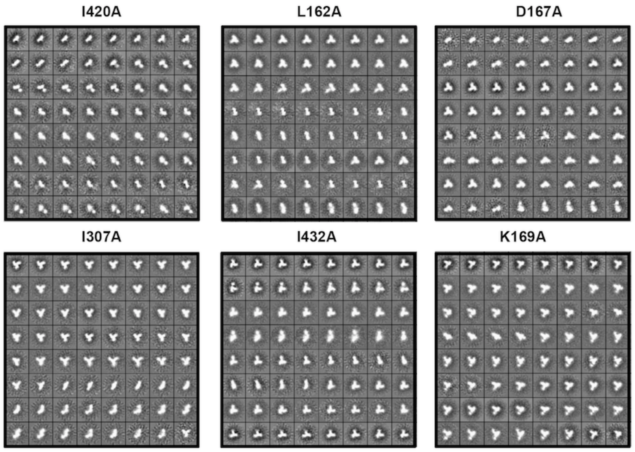

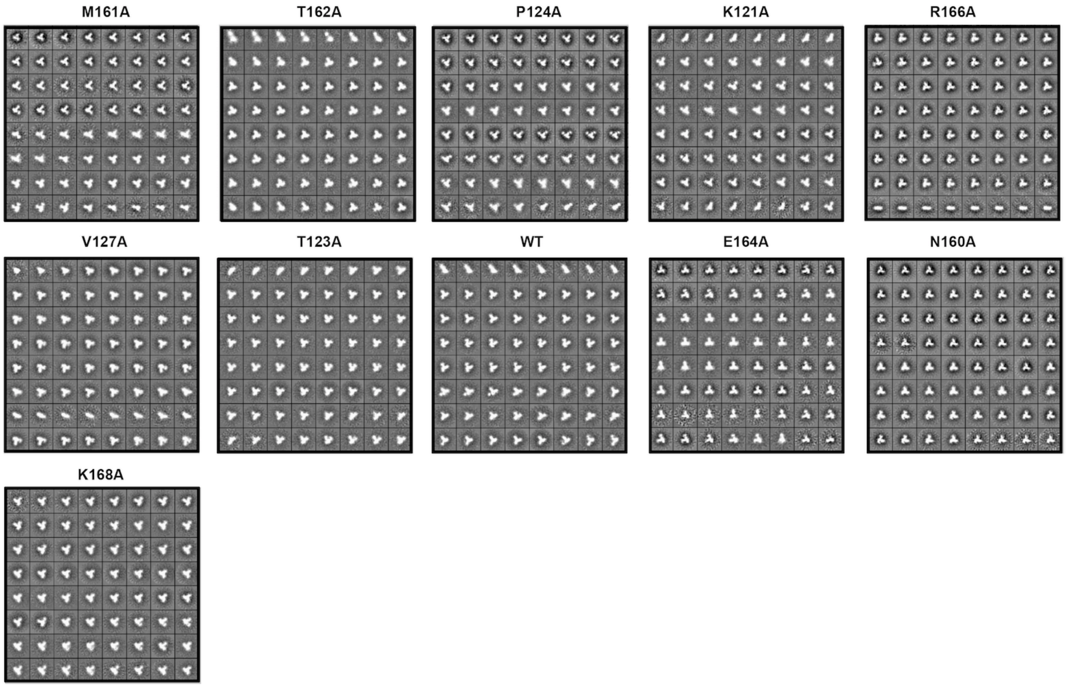

Figure S5. Trimer apex epitope mutations and the effect of point mutations on trimer integrity. Related to Figure 3.

(A) Fold changes in neutralization IC_{50} measured in Figure 2G, relative to the WT BG505 pseudovirus.

(B) Percentage population of open or closed-native like trimers, and non-native trimers (including monomers and dimers) observed in each of the BG505 mutants by negative stain EM, listed in order of lowest to highest % total native-like trimers.

(C) Equivalent point mutations as those in the BG505 pseudoviruses in (A) made in soluble BG505 SOSIP.664 trimers analyzed by negative stain EM. Shown in the same order as listed in (B).

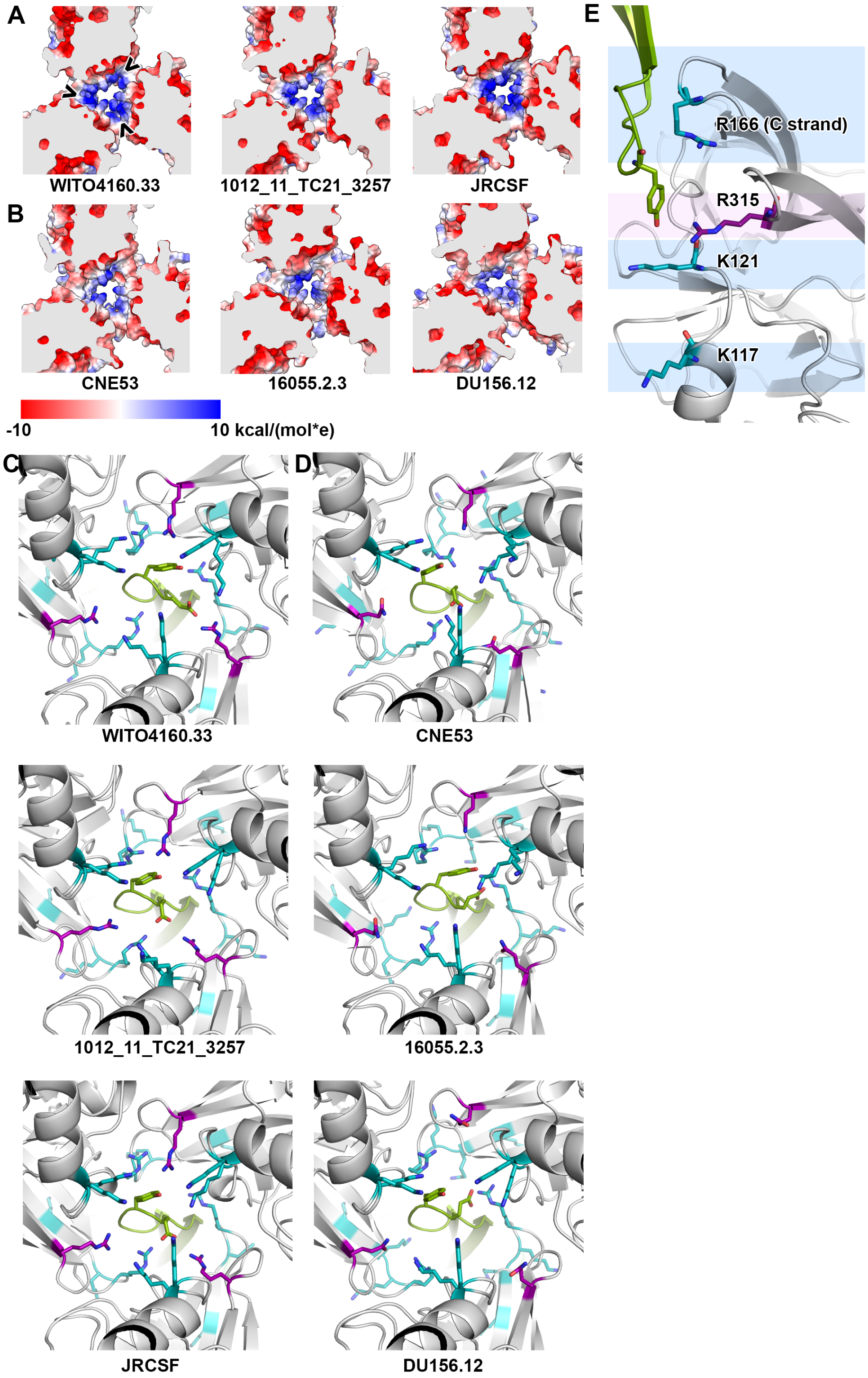

Figure S6. Clade B trimers have an additional electropositive layer in the trimer apex core. Related to Figure 4.

(A) Coulombic potential maps of clade B gp120 trimer homology models relaxed in the presence of PGT145 Fab shows the electrostatic potential at the core of the apex.

(B) Coulombic potential maps of clade C gp120 homology models.

(C) Close up of the regions shown in (A) in the presence of PGT145 HC (green), with positively charged gp120 residues at or close to the PGT145-epitope shown in teal. R315 is shown in purple, and is near the acidic HCDR3 tip side chains (E100h, Y100i, green sticks).

(D) Close up of the clade C apices shown in (B). The clade C trimers have a Q315 residue in place of R315.

(E) The R315 results in an extra electropositive layer (purple), in addition to the conserved C-strand, K121 and K117 layers (blue). Model shown is that of WITO4160.33. The HCDR3 of PGT145 is shown in green.

SUPPLEMENTARY EXPERIMENTAL PROCEDURES

Protein Expression and Purification

Untagged or C-term His_6-tagged BG505 SOSIP.664 trimers were expressed in HEK293S or 293F cells and affinity purified using a 2G12 IgG cross-linked sepharose column as described previously (Julien et al., 2013). Briefly, cells were co-transfected with BG505 SOSIP.664 and furin using a ratio of 4:1, and PEI as the transfection reagent. In some cases, 20 uM of kifunensine was added per L of media at time of transfection. After 5-6 days, the cells were harvested and the supernatant passed over a 2G12 affinity column. Trimers were eluted with 3 M $MgCl_2$ pH 7.4, and dialyzed into 50 mM Tris pH 8, 500 mM NaCl and stored at 4 °C until further use. The affinity-purified trimers were SEC purified a few days prior to structural or biophysical experiments using a HiLoad 26/600 Superdex 200 pg column (GE Healthcare) in 20 mM Tris pH 7.4, 150 mM NaCl (1x TBS), unless stated otherwise.

Antibody IgGs and Fabs were expressed in HEK293F cells with a ratio of 2:1 (HC:LC), using PEI. PGT145, PGDM1400, PGT144 and PGT143 IgGs or Fabs were co-transfected with TPST1 using a ratio of 2:1:1 (HC:LC:TPST1) to ensure tyrosine sulfation. After 5-6 days, cells were harvested and supernatant collected. IgGs were purified using a 5 mL MAb select column (GE Healthcare), and dialyzed into 1x TBS pH 7.4. Fabs were purified using Kappa select column (GE Healthcare). Correctly formed Fabs were separated from LC-LC dimers using a MonoS cation exchange column (GE Healthcare) in 20 mM NaOAc pH 5.6, via a gradient buffer of 20 mM NaOAc pH 5.6, 1M KCl. The correct Fab heterodimer fractions were pooled and further purified by SEC through a Superdex 200 Increase GL 10/30 column (GE Healthcare) in 1x TBS pH 7.4. For PGT143 and PGT144, multiple peaks corresponding to different tyrosine sulfation levels were observed. Protein from each peak was separately purified using size exclusion chromatography with a Superdex 200 10/300 column (GE Healthcare) for crystallization.

Expression and Purification of EnvΔCT-PGT145 Complex

JR-FL EnvΔCT containing the E168K mutant was expressed in 293F cells by co-transfecting the Env gene containing pSVIIIenv vector with Tat at a ratio of 1:3 using 293Fectin. The JR-FL EnvΔCT-PGT145 Fab complex was purified as previously described using PGT145 as the pull-down reagent (Blattner et al., 2014).

Negative Stain EM Data Collection and Processing

All negative stain grids were prepared using 400 Cu mesh carbon coated grids, glow discharged at 20 mAu for 30 seconds prior to usage. All samples were stained as follows: 3 µL of sample per grid, followed by staining with 3 uL NanoW (Nanoprobes) for ~30 sec (for PGT145

bound complexes), or 3 µL 2% uranyl formate (UF, for unliganded trimers) for 45 sec to 1 min. The JR-FL EnvΔCT-PGT145 Fab complex was diluted 1:50 using 1X TBS pH 7.4 without detergent immediately adsorption to grids, and stained using NanoW. All unliganded trimers were diluted to ~0.01 mg/mL with 1X TBS pH 7.4, and stained with 2% UF.

The EnvΔCT-PGT145 Fab complex was imaged on a Tecnai T12 coupled with a Tietz PXL 2k x 2k CCD camera, at a magnification of 52,000x resulting in 2.65 Å/pix images. Images were collected in Leginon (Suloway et al., 2005) using a total dose of ~32 $e^{-}/Å^{2}$ and defocus ranging between 0.5 and 1.0 µm. Particles were picked and stacked using the Appion pipeline (Lander et al., 2009). Reference-free 2D class averages were generated using sxali2d in the Sparx software suite and clustered using Adapt (Frank et al., 1992; Ogura et al., 2003; Ramey et al., 2009).

Unliganded trimer mutants were imaged on a Tecnai T12 microscope coupled with a Tietz TemCam F416 CMOS detector, at a magnification of 52,000x resulting in 2.05 Å/pix on the specimen plane. Images were collected at 1 µm defocus, and particles were stacked as described above. Reference-free 2D classes were generated using MSA/MRA (Ogura et al., 2003) to sort the different trimer forms. Because these trimers were SEC purified over a Superdex 200 Increase 10/300 GL column (GE Healthcare), a large number of the mutants contained a significant portion of particles that corresponded to monomers/dimers. Thus monomer/dimer populations were included in the analysis. In the first round of classification, all classes that had more than one particle in the boxed class average was eliminated. From a second round of 2D classification, reference-free 2D class averages were sub-grouped into three populations; [1] "non-native" that includes monomers, dimers and badly assembled trimers, [2] "native-like closed" and [3] "native-like total" that includes both the closed (population [2]) and "breathing" ("native-like" but open) trimers (Pugach et al., 2015).

CryoEM data collection and processing

BG505 SOSIP.664 trimers produced in 293F cells were pre-complexed with 6-molar excess of 3BNC117 Fab (1 trimer: 6 Fabs) overnight at 4 °C, and purified over a Superose 6 increase column (GE Healthcare). To break the pseudo-symmetry of PGT145, PGT145 Fab was pre-complexed with a mouse Fab obtained from a commercial hybridoma cell line ATCC CRL-1757 (hereon referred to as 1757) that binds the HC of human Fabs (Figures S2A-B). To make the BG505 SOSIP.664-3BNC117-PGT145-1757 complex, PGT145 was combined with 1757 Fab (1:2 molar ratio) and purified over a Superdex 200 increase column (GE Healthcare). The PGT145-1757 complex was then incubated with previously purified BG505 SOSIP-3BNC117 complex, and SEC purified over a Superose 6 increase column in 1x TBS pH 7.4 (GE Healthcare). BG505 SOSIP.664-3BNC117 was concentrated to ~1 mg/mL and supplemented with 0.005%

DDM just prior to freezing. BG505 SOSIP.664-3BNC117-PGT145-1757 was concentrated to ~0.4 mg/mL and supplemented with 0.02% amphipole A8-35 (Anatrace) prior to freezing. The 3 µL of the sample was adsorbed onto 5 second Ar/O_2 plasma cleaned CF-2/2-4C C-Flat holey grids, blotted, then frozen by manual plunging into liquid ethane. Data were collected on an FEI Titan Krios electron microscope operating at 300 KeV coupled with a K2 Summit direct electron detector camera (Gatan) in counting mode in Leginon, at a magnification of 22,500x resulting in a pixel size of 1.31 Å/pixel, using a total dose of ~32 e^-/Å^2. For the BG505 SOSIP.664-3BNC117 complex, a total of 1431 images were collected using a nominal defocus range of 1.2~3 µm. For the BG505 SOSIP.664-3BNC117-PGT145-1757 complex, a total of 1946 images were collected using a nominal defocus range of 1.22-2.5 µm. Data were processed as previously described (Lee et al., 2015) with using RELION 1.4b1, with the exception of the Fab constant domain-masking step in refining the BG505 SOSIP.664-3BNC117 complex. The BG505 SOSIP.664-3BNC117 complex was refined using 22,625 particles with C3 symmetry imposed, to ~4.4 Å resolution at a Fourier shell correlation (FSC) cut-off of 0.143. 1757 was found to be specific for the constant region of the Fab HC, near the protein G binding site (Derrick and Wigley, 1994) (Figure S2F) allowing for confidence in the PGT145 Fab orientation. The BG505 SOSIP.664-3BNC117-PGT145-1757, and BG505 SOSIP.664-3BNC117-PGT145 classes from 3D sorting were combined, resulting in a total of 65,060 particles that were refined without imposing symmetry to 4.7 Å resolution (FSC=0.143). A soft edge mask masking out 1757 Fab and PGT145 Fab constant domains was applied for one additional iteration of focused refinement, resulting in the ~4.3 Å resolution model (FSC=0.143) (Figure S2D).

Model building and Refinement into the CryoEM Maps

The crystal structures of BG505 SOSIP.664 (4TVP) and 3BNC117 Fab (4JPV) were used as templates to generate an initial atomic model using the Modeller plug-in in UCSF Chimera (Pettersen et al., 2004; Webb and Sali, 2016). Each domain was independently docked into the EM density map as a rigid body. The resulting The model was iteratively fixed and refined in Coot (Emsley et al., 2010) and RosettaRelax (DiMaio et al., 2009) employing Ramachandran constraints. Final models were chosen based on a combination of the Rosetta energy score, MolProbity and clash scores (Chen et al., 2010), and EMRinger score (Barad et al., 2015). Glycans were modeled into the finalized protein model as previously described (Lee et al., 2015), with all glycans being modeled as oligomannose. The protein structure in the BG505-3BNC117 model was used as an initial model to refine the PGT145-bound structure. Three copies of the BG505 gp140-3BNC117 subunit of the complex and the Fab variable region of PGT145 were rigid body docked into the BG505-PGT145 EM map and refined as described above. The sulfated tyrosines in the PGT145 Fab X-ray structure were replaced with regular tyrosines because

Rosetta fails to recognize sulfated tyrosines. The complete BG505-3BNC117-PGT145 complex was refined and followed by glycan modeling as was done for the BG505-3BNC117 complex.

Homology Modeling and Refinement

Homology models of various gp120s from clade B and C viruses were generated using SWISS-MODEL (Biasini et al., 2014), with 5CEZ (Garces et al., 2015) as the initial model, as this is the highest resolution Env structure in the PDB. Three copies of the gp120s and PGT145 Fab were docked into a 10 Å low-pass filtered model of the BG505 SOSIP.664-PGT145 complex. The docked pieces were relaxed by RosettaRelax to generate 159 models per gp120. The lowest energy models were analyzed. The electrostatic potential maps were generated in Chimera (Pettersen et al., 2004).

Crystallization

PGT143 and PGT144 Fabs containing different tyrosine sulfation levels were separated by MonoS ion exchange chromatography (GE Healthcare) and concentrated to 4-24 mg/mL. Fab samples were screened for crystallization using the 384 conditions of the JCSG Core Suite (Qiagen) at both 277 and 293 K using the TSRI/IAVI/JCSG robotic Crystalmation system (Rigaku) as described previously (McLellan et al., 2011). After approximately 3 days at 20°C, crystals of PGT143 Fab with no tyrosine sulfation formed in 65% (v/v) 2-methyl-2,4-pentanediol and 0.1 M Tris, pH 8.0 (JCSG Core Suite 4, well C11). Tyrosine sulfated PGT143 did not crystallize in any of the screened conditions. Only PGT144 Fab with one sulfated tyrosine crystallized, and the best diffracting crystals were obtained from crystals grown in JCSG Core Suite, well F8.

X-ray Data collection

Crystals were cryo-protected with glycerol (PGT143: 25%, PGT144: 20%) prior to flash freezing in liquid nitrogen. Data collection was performed at cryogenic temperature (100 K) at beamline 23-ID of the Argonne Photon Source (APS), using a beam wavelength of 1.033 Å. The diffraction data The optimized, hexagonal PGT143 Fab crystals diffracted to 2.4 Å and the diffraction data were indexed, processed and scaled with HKL-2000 (Otwinowski and Minor, 1997) to a completeness of 95.1% with an overall R_{sym} of 11% (49% in the high resolution shell). The optimized PGT144 Fab crystals diffracted to 2.9 Å and the diffraction data were indexed, processed and scaled with XDS to an overall completeness of 96.6% and R_{sym} of 16% (Kabsch, 2010).

Crystal Structure Refinement

Both structures were determined by molecular replacement using Phaser (McCoy et al., 2007) with PGT145 Fab as an initial model (3U1S). Model building was carried out using Coot-0.7 (Emsley et al., 2010) and refinement was implemented with Phenix (Adams et al., 2010). Final R_{cryst}/R_{free} values for PGT143 and PGT144 Fabs are 22.9%/26.7% and 24.1%/28.2%, respectively.

Surface Plasmon Resonance

SPR analysis of PGT145 Fab binding to His-tagged BG505 SOSIP trimers was analyzed on a Biacore 3000 instrument at 25°C. Glycan knockout mutants were expressed in 293F cells unless otherwise indicated. All trimers were purified on a 2G12-affinity column, and immobilized on the chip by His-tag capture, as previously described (Yasmeen et al., 2014). In brief, anti-histidine antibody (GE Healthcare) was amide-coupled to the dextran of a CM5 chip up to a level of 15000 RU. Trimers were captured to yield R_L = 520 RU (s.d. < 1.3 %). Throughout, HBS-EP (10 mM HEPES pH 7.4, 150 mM NaCl, 3 mM EDTA, 0.002 % P20 surfactant) was used as running buffer. The maximum flow rate (50 µL/min) was used during analyte binding to minimize mass-transport limitation, the near absence of which was confirmed by global fits of mass transfer coefficients. Association was monitored for 5 min and dissociation for 10 min, the Fab being titrated from 1000 to 2 nM in consecutive cycles. After each cycle, the capture-antibody surface was regenerated by an injection of 10 mM glycine pH 2 for 120 s at a flow rate of 30 µL/min. The signals from both 0-analyte injections and the parallel control channel were subtracted. A Langmuir model (Biavaluation, GE Healthcare) was fitted globally to the binding data to derive the kinetic parameters. S_m values were based on the highest analyte concentrations, yielding the highest T values for R_{max}. All reported binding parameters were significant (T > 10).

To study interference or enhancement of CD4 and PGT145 binding, sequential binding analyses were also performed. The association and dissociation phases of binding to trimers immobilized as above were monitored for 200 s each. PGT145 IgG and CD4-IgG2 were injected at 500 nM, sequentially (at times 0 and 200 s in a single cycle, both at flow rates of 30 µL/min), as described (Derking et al., 2015). A second injection of PGT145 resulted in a residual binding of 2%, relative to the first PGT145 injection; correspondingly, for CD4-IgG2 after a first injection of the CD4-IgG2, the residual binding was 14%, showing that the first injection nearly saturated the respective binding sites. The relative residual binding of PGT145 after CD4-IgG2 injection, or vice versa, was calculated analogously in %, as the binding of analyte 2 after pre-binding of analyte 1, relative to the binding of analyte 2 on its own.

Neutralization Assays

Amino acid substitution mutations in the antibody and HIV envelope encoding plasmids

were incorporated by QuikChange site-directed mutagenesis kit (Stratagene) according to the manufacturer's instructions. For pseudovirus production, we cotransfected HEK293T or 293S cells with an Env encoding and an Env-deficient backbone (pSG3DEnv) plasmids with the transfection reagent Fugene 6 (1:2 ratio) (Promega). Pseudoviruses were harvested 48–72 hr post-transfection, filtered and titrated for use in neutralization assays. Neutralization was measured in TZM-bl target cells, as described previously (Andrabi et al., 2015).

Glycan Array Assays

mAbs were screened on a custom high-mannose array, consisting of 9 mannosides and 1 control sialylated N-glycan. The 10 amine-linked glycans were covalently immobilized onto custom NHS-ester dendron functionalized glass microscope slides (G3 and G4, ZBiotech) using a MicroGridII robotic array printer (Digilab Global) equipped with Stealth SMP4B microarray pins (Telechem). Compounds were diluted to 100 µM and spotted in replicates of 6 and, following 1 h of humidification, were washed in blocking buffer (50 mM ethanolamine in 50 mM borate buffer, pH 9.2) to remove any unbound compound and quench remaining NHS-ester residues. Slides were stored at -20°C prior to use. To assess mAb binding, the antibodies were pre-mixed with the detection antibody (anti-human-IgG R-PE, Jackson Immuno) at a ratio of 2:1, 30 ug/mL and 15 µg/mL, respectively. Following 15 min, the pre-complexed antibodies were applied directly to the slide surface and allowed to incubate for 1 h and then washed. Arrays were washed using three exchanges of 0.05% Tween-20 in 1x PBS, three exchanges of 1x PBS and finally, three exchanges of deionized water. Washed arrays were dried by centrifugation and scanned for R-PE signal on a confocal microarray scanner (Innoscan 1100AL, Innopsys). The resultant images were analyzed using Mapix (Innopsys) and mean signal minus background values for each spotted compound were calculated and plotted using MS Excel.

Identification of Key Residues by Regression Analysis

TZM-bl neutralization assay derived IC_{50} values of PGT145 from a total of 106 strains was obtained from Sok *et al.* (Sok et al., 2014). An additional dataset of IC_{50} values from a total of 65 strains against 293T produced pseudoviruses was obtained from the neutralization assay performed here (Figure 2A), and from Walker *et al.* (Walker et al., 2011) for which the Env sequences were available. In these two datasets, 37 strains overlapped. Because BG505 was not a part of either panel, the PGT145 IC_{50} against BG505 was obtained from the data shown in Fig. 3E. Subsequently, IC_{50} from 135 strains was employed for downstream analysis. For those strains that were included in both datasets, IC_{50} was obtained by averaging between two datasets. Strains with IC_{50} > 10 µg/mL were classified as escape. Multiple sequence alignment was performed by MUSCLE using default parameters (Edgar, 2004). The residues of interest

were extracted. By comparing the amino acid identity of each residue of interest in each strain to that of BG505, a numeric value was assigned based on an adjusted BLOSUM62 matrix (Henikoff and Henikoff, 1992). For each substitution relative to a given amino acid at BG505, the numeric value was computed by subtracting the substitution score from the self-substitution score. For example, at a given residue, if a strain had the same amino acid identity as that of BG505, a value of 0 would be assigned for that particular residue in that strain. If the amino acid identity were different from that of BG505, a negative value would be assigned. This negative value would represent the conservativeness of the substitution, with less conservative being more negative. As a result, the amino-acid sequence for each strain was converted to a list of integers that has the same length as the amino acid sequence string of interest. Combining these sequences in the integer representation generated a matrix. A logistic regression model with L1 regularization was then fit to the matrix with the escape phenotype as the targets. Logistic regression was performed using "linear_model.LogisticRegressionCV" in scikit-learn (Pedregosa et al., 2011) in python. Each residue of interest would be assigned a coefficient. A larger magnitude of coefficient a residue implied more influence it has on the escape phenotype. The absolute value of the coefficient was reported. The global Env sequence conservation information was derived from an alignment of 25,946 sequences across multiple clades from the Los Alamos National Laboratory HIV database.

SUPPLEMENTAL REFERENCES

Adams, P.D., Afonine, P.V., Bunkoczi, G., Chen, V.B., Davis, I.W., Echols, N., Headd, J.J., Hung, L.W., Kapral, G.J., Grosse-Kunstleve, R.W., *et al.* (2010). PHENIX: a comprehensive Python-based system for macromolecular structure solution. Acta Crystallogr., Sect: D Biol. Crystallogr. *66*, 213-221.

Andrabi, R., Voss, J.E., Liang, C.H., Briney, B., McCoy, L.E., Wu, C.Y., Wong, C.H., Poignard, P., and Burton, D.R. (2015). Identification of common features in prototype broadly neutralizing antibodies to HIV envelope V2 apex to facilitate vaccine design. Immunity *43*, 959-973.

Barad, B.A., Echols, N., Wang, R.Y., Cheng, Y., DiMaio, F., Adams, P.D., and Fraser, J.S. (2015). EMRinger: side chain-directed model and map validation for 3D cryo-electron microscopy. Nature Methods *12*, 943-946.

Biasini, M., Bienert, S., Waterhouse, A., Arnold, K., Studer, G., Schmidt, T., Kiefer, F., Gallo Cassarino, T., Bertoni, M., Bordoli, L., and Schwede, T. (2014). SWISS-MODEL: modelling protein tertiary and quaternary structure using evolutionary information. Nucleic acids research *42*, W252-258.

Blattner, C., Lee, J.H., Sliepen, K., Derking, R., Falkowska, E., de la Pena, A.T., Cupo, A., Julien, J.P., van Gils, M., Lee, P.S., *et al.* (2014). Structural delineation of a quaternary, cleavage-dependent epitope at the gp41-gp120 interface on intact HIV-1 Env trimers. Immunity *40*, 669-680.

Chen, V.B., Arendall, W.B., 3rd, Headd, J.J., Keedy, D.A., Immormino, R.M., Kapral, G.J., Murray, L.W., Richardson, J.S., and Richardson, D.C. (2010). MolProbity: all-atom structure validation for macromolecular crystallography. Acta Crystallogr., Sect: D Biol. Crystallogr. *66*, 12-21.

Derking, R., Ozorowski, G., Sliepen, K., Yasmeen, A., Cupo, A., Torres, J.L., Julien, J.P., Lee, J.H., van Montfort, T., de Taeye, S.W., *et al.* (2015). Comprehensive antigenic map of a cleaved soluble HIV-1 envelope trimer. PLoS Pathog. *11*, e1004767.

Derrick, J.P., and Wigley, D.B. (1994). The third IgG-binding domain from streptococcal protein G. An analysis by X-ray crystallography of the structure alone and in a complex with Fab. J. Mol. Biol. *243*, 906-918.

DiMaio, F., Tyka, M.D., Baker, M.L., Chiu, W., and Baker, D. (2009). Refinement of protein structures into low-resolution density maps using rosetta. J. Mol. Biol. *392*, 181-190.

Edgar, R.C. (2004). MUSCLE: multiple sequence alignment with high accuracy and high throughput. Nucleic Acids Res. *32*, 1792-1797.

Emsley, P., Lohkamp, B., Scott, W.G., and Cowtan, K. (2010). Features and development of Coot. Acta Crystallogr., Sect: D Biol. Crystallogr. *66*, 486-501.

Frank, J., Penczek, P., and Liu, W. (1992). Alignment, classification, and three-dimensional reconstruction of single particles embedded in ice. Scanning Microsc. Suppl. *6*, 11-20; discussion 20-12.

Garces, F., Lee, J.H., de Val, N., Torrents de la Pena, A., Kong, L., Puchades, C., Hua, Y., Stanfield, R.L., Burton, D.R., Moore, J.P.*, et al.* (2015). Affinity maturation of a potent family of HIV antibodies Is primarily focused on accommodating or avoiding glycans. Immunity *43*, 1053-1063.

Henikoff, S., and Henikoff, J.G. (1992). Amino acid substitution matrices from protein blocks. Proc. Natl. Acad. Sci. USA *89*, 10915-10919.

Julien, J.P., Cupo, A., Sok, D., Stanfield, R.L., Lyumkis, D., Deller, M.C., Klasse, P.J., Burton, D.R., Sanders, R.W., Moore, J.P.*, et al.* (2013). Crystal structure of a soluble cleaved HIV-1 envelope trimer. Science *342*, 1477-1483.

Kabsch, W. (2010). Xds. Acta Crystallogr., Sect: D Biol. Crystallogr. *66*, 125-132.

Lander, G.C., Stagg, S.M., Voss, N.R., Cheng, A., Fellmann, D., Pulokas, J., Yoshioka, C., Irving, C., Mulder, A., Lau, P.W.*, et al.* (2009). Appion: an integrated, database-driven pipeline to facilitate EM image processing. J. Struct. Biol. *166*, 95-102.

Lee, J.H., de Val, N., Lyumkis, D., and Ward, A.B. (2015). Model building and refinement of a natively glycosylated HIV-1 Env protein by high-resolution cryoelectron microscopy. Structure *23*, 1943-1951.

McCoy, A.J., Grosse-Kunstleve, R.W., Adams, P.D., Winn, M.D., Storoni, L.C., and Read, R.J. (2007). Phaser crystallographic software. J. Appl. Crystallogr. *40*, 658-674.

McLellan, J.S., Pancera, M., Carrico, C., Gorman, J., Julien, J.P., Khayat, R., Louder, R., Pejchal, R., Sastry, M., Dai, K., *et al.* (2011). Structure of HIV-1 gp120 V1/V2 domain with broadly neutralizing antibody PG9. Nature *480*, 336-343.

Ogura, T., Iwasaki, K., and Sato, C. (2003). Topology representing network enables highly accurate classification of protein images taken by cryo electron-microscope without masking. J. Struct. Biol. *143*, 185-200.

Otwinowski, Z., and Minor, W. (1997). Processing of X-ray diffraction data collected in oscillation mode. Methods Enzymol. *276A*, 307-326.

Pedregosa, F., Varoquaux, G., Gramfort, A., Michel, V., Thirion, B., Grisel, O., Blondel, M., Prettenhofer, P., Weiss, R., Dubourg, V., *et al.* (2011). Scikit-learn: machine learning in Python. J. Mach. Learn. Res. *12*, 2825-2830.

Pettersen, E.F., Goddard, T.D., Huang, C.C., Couch, G.S., Greenblatt, D.M., Meng, E.C., and Ferrin, T.E. (2004). UCSF Chimera--a visualization system for exploratory research and analysis. J. Comput. Chem. *25*, 1605-1612.

Pugach, P., Ozorowski, G., Cupo, A., Ringe, R., Yasmeen, A., de Val, N., Derking, R., Kim, H.J., Korzun, J., Golabek, M., *et al.* (2015). A native-like SOSIP.664 trimer based on an HIV-1 subtype B Env gene. J. Virol. *89*, 3380-3395.

Ramey, V.H., Wang, H.W., and Nogales, E. (2009). Ab initio reconstruction of helical samples with heterogeneity, disorder and coexisting symmetries. J. Struct. Biol. *167*, 97-105.

Sok, D., van Gils, M.J., Pauthner, M., Julien, J.P., Saye-Francisco, K.L., Hsueh, J., Briney, B., Lee, J.H., Le, K.M., Lee, P.S., *et al.* (2014). Recombinant HIV envelope trimer selects for quaternary-dependent antibodies targeting the trimer apex. Proc. Natl. Acad. Sci. USA *111*, 17624-17629.

Suloway, C., Pulokas, J., Fellmann, D., Cheng, A., Guerra, F., Quispe, J., Stagg, S., Potter, C.S., and Carragher, B. (2005). Automated molecular microscopy: the new Leginon system. J. Struct. Biol. *151*, 41-60.

Walker, L.M., Huber, M., Doores, K.J., Falkowska, E., Pejchal, R., Julien, J.P., Wang, S.K., Ramos, A., Chan-Hui, P.Y., Moyle, M.*, et al.* (2011). Broad neutralization coverage of HIV by multiple highly potent antibodies. Nature *477*, 466-470.

Webb, B., and Sali, A. (2016). Comparative protein structure modeling using MODELLER. Curr. Protoc. Bioinformatics *54*, 5 6 1-5 6 37.

Yasmeen, A., Ringe, R., Derking, R., Cupo, A., Julien, J.P., Burton, D.R., Ward, A.B., Wilson, I.A., Sanders, R.W., Moore, J.P., and Klasse, P.J. (2014). Differential binding of neutralizing and non-neutralizing antibodies to native-like soluble HIV-1 Env trimers, uncleaved Env proteins, and monomeric subunits. Retrovirology *11*, 41.
